# Synthesis and Antileukemia Activity Evaluation of Benzophenanthridine Alkaloid Derivatives

**DOI:** 10.3390/molecules27123934

**Published:** 2022-06-19

**Authors:** Yaling Tang, Xinglian Xu, Jiang Li, Lulu Deng, Shuzhen Mu

**Affiliations:** 1 College of Pharmacy, Guizhou University, Guiyang 550025, China; gs.yltang19@gzu.edu.cn; 2 State Key Laboratory of Functions and Applications of Medicinal Plants, Guizhou Medical University, Guiyang 550014, China; xinglian_xu2022@163.com (X.X.); jiangli@gzcnp.cn (J.L.); 3 The Key Laboratory of Chemistry for Natural Products of Guizhou Province, Chinese Academy of Sciences, Guiyang 550014, China

**Keywords:** *Zanthoxylum nitidum*, benzophenanthridine alkaloid derivatives, synthesis, antileukemia activity, cell cycle and apoptosis

## Abstract

Thirty-three benzophenanthridine alkaloid derivatives (**1a**–**1u** and **2a**–**2l**) were synthesized, and their cytotoxic activities against two leukemia cell lines (Jurkat Clone E6-1 and THP-1) were evaluated in vitro using a Cell Counting Kit-8 (CCK-8) assay. Nine of these derivatives (**1i**–**l**, **2a,** and **2i**–**l**) with IC_50_ values in the range of 0.18–7.94 μM showed significant inhibitory effects on the proliferation of both cancer cell lines. Analysis of the primary structure–activity relationships revealed that different substituent groups at the C-6 position might have an effect on the antileukemia activity of the corresponding compounds. In addition, the groups at the C-7 and C-8 positions could influence the antileukemia activity. Among these compounds, **2j** showed the strongest in vitro antiproliferative activity against Jurkat Clone E6-1 and THP-1 cells with good IC_50_ values (0.52 ± 0.03 μM and 0.48 ± 0.03 μM, respectively), slightly induced apoptosis, and arrested the cell-cycle, all of which suggests that compound **2j** may represent a potentially useful start point to undergo further optimization toward a lead compound.

## 1. Introduction

Leukemia is a broad term for a group of blood cell cancers that begin in stem cells found in the bone marrow. Leukemia occurs most often in adults older than 55, but it is also the most common cancer in children younger than 15; in particular, the incidence rates of leukemia are the highest in early childhood and later adulthood [1,2]. Patients with leukemia usually have serious complications, such as autoimmune cytopenia [3], bleeding [4], electrolyte imbalance, and hyperuricemia [5]; therefore, leukemia seriously threatens human health and quality of life.

Currently, chemotherapy and hematopoietic stem-cell bone marrow transplantation are still the main treatments for leukemia. However, bone marrow transplantation involves a complicated process that requires antigen compatibility between the donor and recipient. Although these methods can lead to remission in most patients, the recurrence rate is very high, and the long-term survival rate is low. Notably, high-dose combination chemotherapy can cause patients to develop drug resistance and serious side-effects, such as bone marrow suppression, gastrointestinal reactions, and cardiotoxicity [6,7,8,9].

Among the many available drugs to treat leukemia, imatinib and all-*trans*-retinoic acid (ATRA) plus arsenic trioxide (ATO) are widely used worldwide. Although there has been great success with reducing the symptoms of patients with leukemia after treatment with these drugs, the side-effects and early mortality they can cause remain significant, which are major barriers to treating leukemia patients [10,11]. In recent years, a number of new treatments for leukemia emerged. For example, CEP-701, which is an FLT3 inhibitor, was assessed in leukemia, with the hope that it represents the development of an important new molecularly targeted therapy for this disease [12,13]. Therefore, whether to improve the treatment and long-term survival rates of patients with leukemia, or to find new treatments for leukemia, it is extremely important to research and develop effective new drugs.

Natural products, as an important source of drugs and drug lead compounds, have the advantages of unique mechanisms, remarkable results, low toxicity, and few side-effects. Many well-known natural products with various applications, such as artemisinin, paclitaxel, and vinblastine, come from a wide variety of Chinese herbal medicines found abundantly in China. Therefore, drug candidates for treating leukemia could be obtained through the structural optimization of natural lead compounds. *Z. nitidum* is an important Chinese herbal medicine that possesses various antitumor active ingredients. Benzophenanthridine alkaloids are some of the most important active ingredients abundantly found in this plant. At present, this type of alkaloid has been found to have a variety of biological activities with antibacterial [14,15,16], analgesic, anti-inflammatory [17], antiviral [18], anti-phytopathogenic [19] and antitumor [20,21,22,23,24,25] effects. However, there are few reports on the antileukemia activity of benzophenanthridine alkaloids.

**Figure 1:**
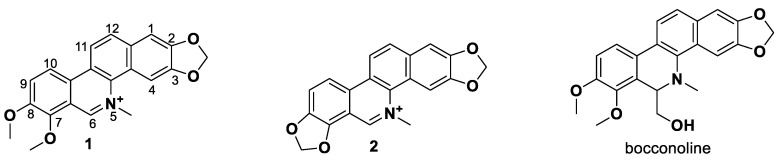
The structures of chelerythrine (**1**), sanguinarine (**2**), and bocconoline.

Our previous studies indicated that certain benzophenanthridine alkaloids showed strong inhibitory effects on leukemia cell lines [26,27]. To continue our research, two active benzophenanthridine alkaloids, chelerythrine (**1**) and sanguinarine (**2**) (Figure 1), which were found in high abundance, were selected as the starting compounds for structural modification to obtain antileukemia drug candidates with better activity. Therefore, thirty-three benzophenanthridine alkaloid derivatives (**1a**–**1u** and **2a**–**2l**) were synthesized, and their antileukemia activities against two leukemia cell lines (Jurkat Clone E6-1 and THP-1) were evaluated in vitro (According to the preliminary screening results in Table S2 in the Supplementary Materials, we selected these two leukemia cells for activity test). Among them, nine derivatives (**1i**–**l**, **2a** and **2i**–**l**) showed significant inhibitory effects on the proliferation of Jurkat Clone E6-1 and THP-1 cells. In particular, compound **2j** displayed the strongest inhibition against Jurkat Clone E6-1 and THP-1 cells, with IC_50_ values of 0.52 ± 0.03 μM and 0.48 ± 0.03 μM, respectively. Furthermore, the influence of compound **2j** on the cell-cycle and apoptosis in both leukemia cell lines was tested.

Herein, we report the synthesis and antileukemia activity evaluation of a series of novel benzophenanthridine alkaloid derivatives of chelerythrine (**1**) and sanguinarine (**2**) (**1a**–**1u** and **2a**–**2l**). Their cytotoxic activities and initial structure–activity relationships (SARs) are also reported.

## 2. Results and Discussion

### 2.1. Design and Synthesis of the Benzophenanthridine Alkaloid Derivatives

**Figure 2:**
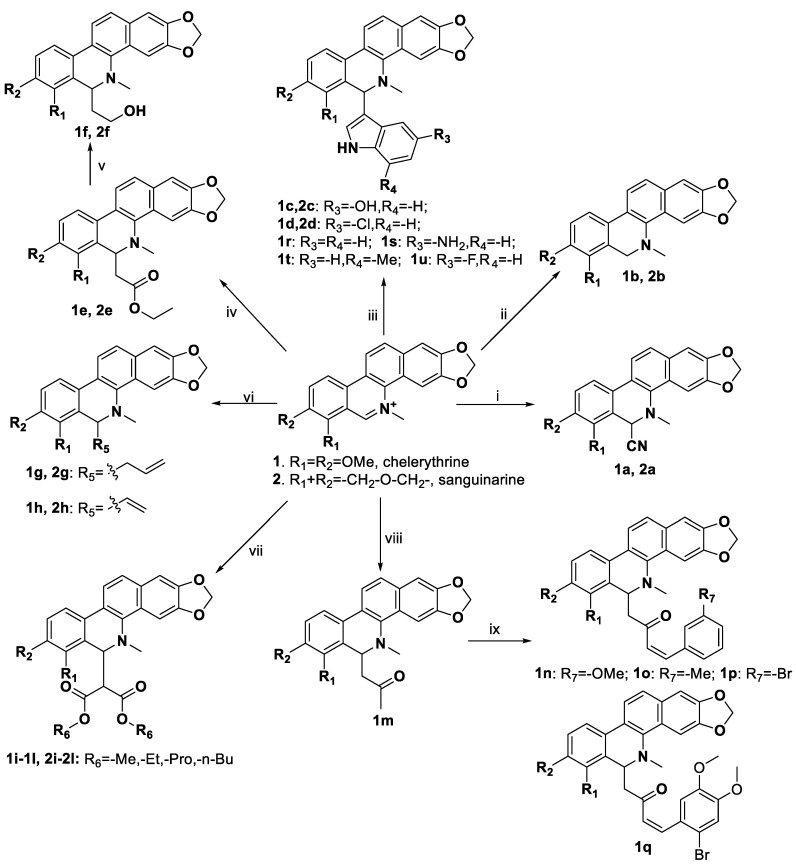
Reagents and reaction conditions: (**i**) TMSCN, DMAP, DCM, reflux, 14 h; (**ii**) NaBH_4,_ MeOH, r.t., 0.5 h; (**iii**) CH_3_CN, r.t., 3–8 h; (**iv**) ethyl trimethylsilylacetate, CsF, CH_3_CN, r.t., 4–5 h; (**v**) LiAlH_4_, THF, 5 °C, 0.5 h; (**vi**) CH_3_CN, r.t., 3–4 h; (**vii**) CH_3_CN, r.t., 5–14 h; (**viii**) CH_3_COCH_3_, 20% Na_2_CO_3_, reflux, 24 h; (**ix**) PhCOOH, piperidine, toluene, reflux, 24 h.

In our previous studies, bocconoline (Figure 1), a benzophenanthridine alkaloid isolated from *Z. nitidum,* showed good antiproliferative effects on leukemia cells and low toxicity [27]. It differs structurally from other benzophenanthridine alkaloids due to the hydroxymethyl group at the C-6 position. Therefore, it was speculated that this substitution might play a crucial role in reducing the toxicity of this compound. In addition, through literature investigation, we found that the introduction of appropriate groups (malonic esters, dialkyl phosphites, nitro alkanes, or indoles) at the C-6 position could enhance its activities [21]. Hence, in order to discover more benzophenanthridine alkaloid derivatives with good antileukemia activity and low toxicity, two natural benzophenanthridine alkaloids with good antileukemia activity, chelerythrine (**1**) and sanguinarine (**2**), were chosen as starting points, and a series of their derivatives, **1a**–**1u** and **2a**–**2l**, were synthesized by changing the substituent at the C-6 position (The ^1^H- and ^13^C-NMR, HR-ESI-MS and HPLC spectra of all compounds S5–S103, S106–S138 are shown in the Supplementary Materials). The synthetic routes for the target compounds are shown in Figure 2. Briefly, structural modification of chelerythrine (**1**) and sanguinarine (**2**) mainly involved changing the substituent at the C-6 position by nucleophilic addition, including the introduction of cyano [28], indole, malonic ester [21], ester [29], allyl [30], and acetonyl units. To obtain compounds **1b** and **2b**, reduction of the C=N double bond at the C-6 position was achieved by treatment with NaBH_4_ [14]. The ethyl acetate units in compounds **1e** and **2e** were converted to hydroxyethyls in compounds **1f** and **2f** with LiAlH_4_ [31]. Additionally, compounds **1n**–**q** were synthesized via the Claisen–Schmidt reaction [32].

### 2.2. In Vitro Antileukemia Activity and SAR Analysis

#### 2.2.1. Inhibitory Effects on Leukemia Cell Proliferation

The cytotoxic activities of the 33 synthesized derivatives tested at 20 μM were evaluated in two leukemia cell lines (Jurkat Clone E6-1 and THP-1) using a Cell Counting Kit-8 (CCK-8) assay with doxorubicin hydrochloride (DOX) as a positive control. The results of the preliminary bioassay are listed in Table 1.

As shown in Table 1, the in vitro activity data revealed that nine derivatives (**1i**–**l**, **2a,** and **2i**–**l**) showed significant inhibitory effects on these leukemia cell lines with IC_50_ values ranging from 0.5 to 8.0 μM and from 0.1 to 6.0 μM, respectively. Notably, compounds **2a** and **2j** exhibited excellent activities in both cell lines with good IC_50_ values of 0.53 ± 0.05 μM and 0.52 ± 0.03 μM for Jurkat Clone E6-1 and 0.18 ± 0.03 μM and 0.48 ± 0.03 μM for THP-1, respectively. From the results of the activity data, it could be seen that compound **2a** showed good activity, but **2a** could not show a good dose dependence in the further study of the cell cycle and apoptosis. However, compound **2j** showed a better dose dependence than **2a**. Therefore, compound **2j** might be a potential antileukemia compound and was chosen for further evaluation.


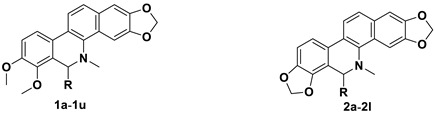


**Table 1:** IC_50_ values of 33 derivatives against leukemia cell lines in vitro ($\bar{x}$ ± SD, *n* = 3).

Compound	R	IC_50_ (μM)	
		Jurkat Clone E6-1	THP-1
**1a**	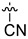	>20	>20
**1b**	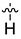	>20	>20
**1c**	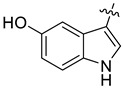	>20	>20
**1d**	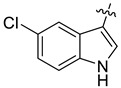	>20	>20
**1e**	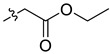	>20	>20
**1f**	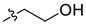	>20	>20
**1g**	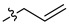	>20	>20
**1h**	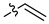	>20	>20
**1i**	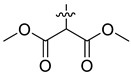	7.94 ± 0.10	5.78 ± 0.23
**1j**	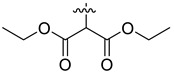	2.61 ± 0.19	1.87 ± 0.02
**1k**	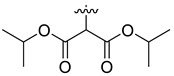	2.48 ± 0.13	4.45 ± 0.34
**1l**	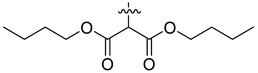	5.64 ± 0.20	5.88 ± 0.07
**1m**	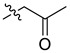	>20	>20
**1n**	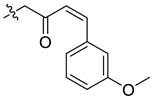	>20	>20
**1o**	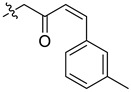	>20	>20
**1p**	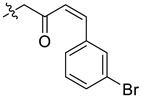	>20	>20
**1q**	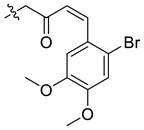	>20	>20
**1r**	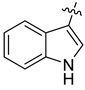	>20	>20
**1s**	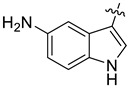	>20	>20
**1t**	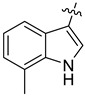	>20	>20
**1u**	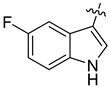	>20	>20
**2a**	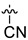	0.53 ± 0.05	0.18 ± 0.03
**2b**	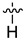	>20	>20
**2c**	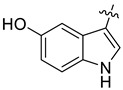	>20	>20
**2d**	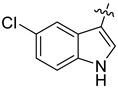	>20	>20
**2e**	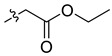	>20	>20
**2f**	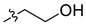	>20	>20
**2g**	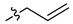	>20	>20
**2h**	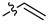	>20	>20
**2i**	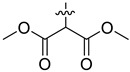	1.30 ± 0.05	1.46 ± 0.06
**2j**	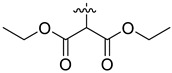	0.52 ± 0.03	0.48 ± 0.03
**2k**	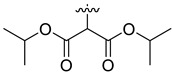	1.23 ± 0.08	1.38 ± 0.04
**2l**	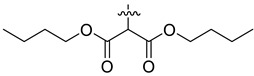	0.91 ± 0.04	1.17 ± 0.13
**1**		5.58 ± 0.13	4.70 ± 0.07
**2**		1.56 ± 0.09	1.60 ± 0.13
Doxorubicin hydrochloride		0.12 ± 0.01	0.10 ± 0.01

#### 2.2.2. Effects of Compound **2j** on Cellular Apoptosis

**Figure 3:**
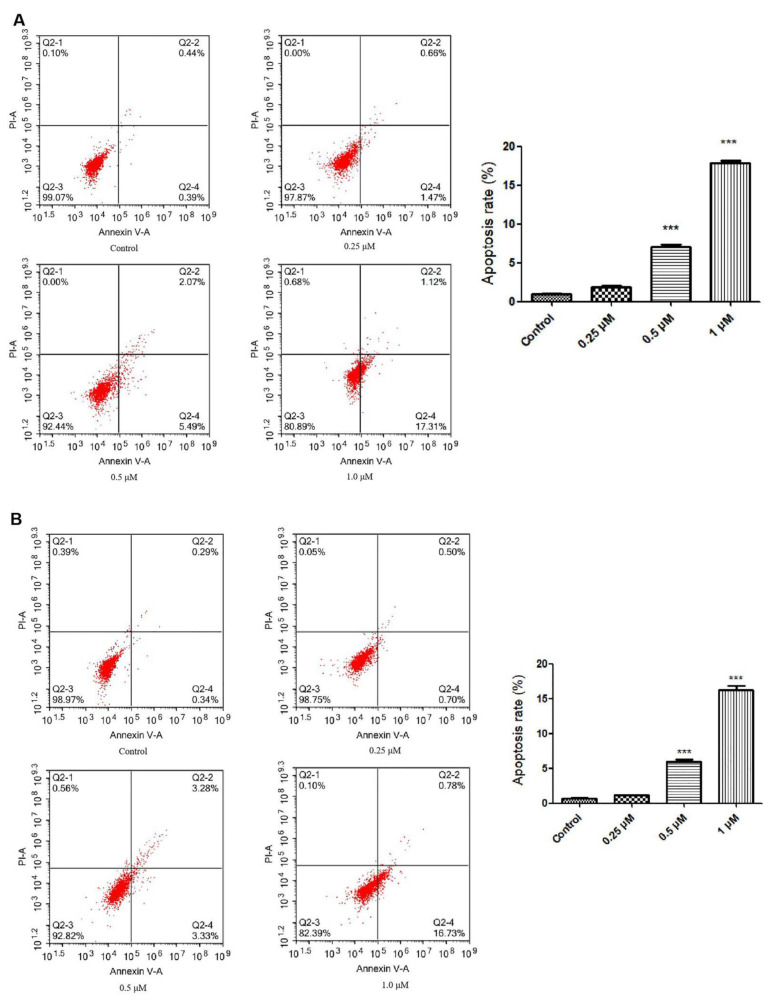
Compound **2j** induced apoptosis in Jurkat Clone E6-1 and THP-1 cell lines. (**A**) Compound **2j** induced apoptosis in Jurkat Clone E6-1 cell line. Jurkat Clone E6-1 cells were treated with 0.25, 0.5, and 1.0 µM of compound **2j** for 48 h, and cells were subsequently stained with Annexin V–FITC/PI and subsequently analyzed by flow cytometry. (**B**) Compound **2j** induced apoptosis in THP-1 cell line. THP-1 cells were treated with 0.25, 0.5, and 1.0 µM of compound **2j** for 48 h, and cells were subsequently stained with Annexin V–FITC/PI and subsequently analyzed by flow cytometry. All data are presented as means ± SD (*n* = 3); *** *p* < 0.001 vs. the control group.

Two leukemia cell lines (Jurkat Clone E6-1 and THP-1) were treated with compound **2j** at concentrations of 0.25, 0.5, and 1.0 μM for 48 h at 37 °C. The apoptosis rates induced by treatment with compound **2j** are shown in Figure 3.

Compared with the control group (0.92% ± 0.09% for Jurkat Clone E6-1 cells and 0.69% ± 0.08% for THP-1 cells), after treating the cells with compound **2j** for 48 h, the rates of apoptosis increased in a dose-dependent manner. When Jurkat Clone E6-1 and THP-1 cells were treated with 0.5 μM and 1.0 μM compound **2j**, their apoptosis rates increased significantly from 7.07% ± 0.43% to 17.84% ± 0.65% and from 6.01% ± 0.52% to 16.23% ± 1.15%, respectively (*p* < 0.001 vs. control group). These results suggested that compound **2j** could slightly induce apoptosis in these two leukemia cell lines.

#### 2.2.3. Effects of Compound **2j** on the Cell-Cycle

**Figure 4:**
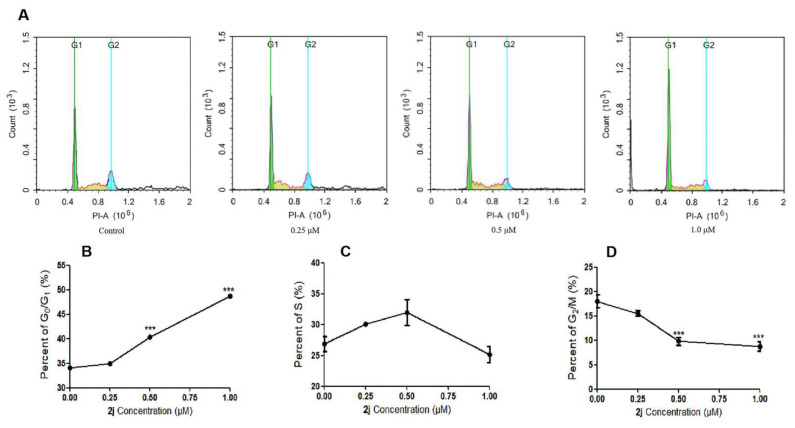

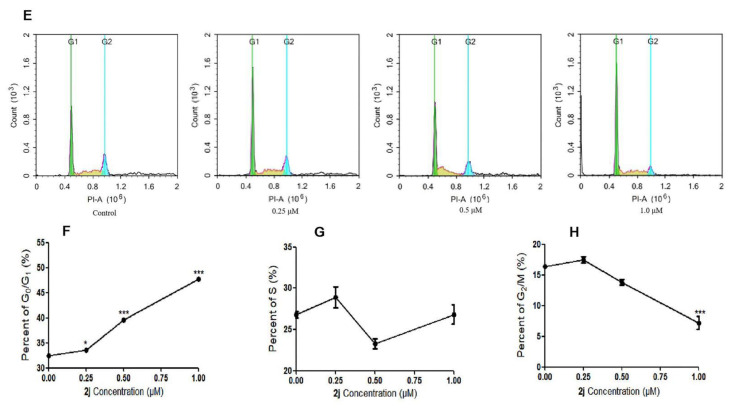
Effects of compound **2j** on Jurkat Clone E6-1 and THP-1 cell-cycle. (**A**) The cell-cycle distribution of Jurkat Clone E6-1 using flow cytometry. (**B**) The percentage of Jurkat Clone E6-1 cells in the G_0_/G_1_ phase. (**C**) The percentage of Jurkat Clone E6-1 cells in the S phase. (**D**) The percentage of Jurkat Clone E6-1 cells in the G_2_/M phase. (**E**) The cell-cycle distribution of THP-1 using flow cytometry. (**F**) The percentage of THP-1 cells in the G_0_/G_1_ phase. (**G**) The percentage of THP-1 cells in the S phase. (**H**) The percentage of THP-1 cells in the G_2_/M phase. All data are presented as means ± SD (*n* = 3); * *p* < 0.05 and *** *p* < 0.001 vs. the control group.

Cell-cycle assays in the Jurkat Clone E6-1 and THP-1 cell lines were performed using flow cytometry, and the results are shown in Figure 4. When these two types of cells were treated with compound **2j** for 48 h at concentrations ranging from 0 μM to 1.0 μM, the numbers of cells in the G_0_/G_1_ phase increased significantly in a dose-dependent manner, which was accompanied by decreases in the G_2_/M populations. However, the percentages of cells in the S phase were not significantly different. These results showed that compound **2j** could induce cell-cycle arrest in the G_0_/G_1_ phase.

#### 2.2.4. SAR Analysis

As shown in Table 1, preliminary SAR studies were undertaken on the basis of the above cytotoxicity evaluation. Among the compounds synthesized, nine derivatives (**1i**–**l**, **2a,** and **2i**–**l**) showed significant inhibitory effects on the proliferation of Jurkat Clone E6-1 and THP-1 cells with IC_50_ values ranging from 0.1 to 8.0 μM. However, the other derivatives displayed weak or no inhibitory activity against the two leukemia cell lines. These results indicated that compounds containing cyano and malonic esters groups at the C-6 position of the benzophenanthridine alkaloid scaffold showed higher cytotoxic activity than the other types of compounds, and compounds with different substituents at the C-6 position exhibited different inhibitory activities. Compounds **2a** and **2i**–**l** showed much stronger cytotoxicity, with IC_50_ values of 0.53 μM, 1.30 μM, 0.52 μM, 1.23 μM, and 0.91 μM (in Jurkat Clone E6-1 cells) and 0.18 μM, 1.46 μM, 0.48 μM, 1.38 μM, and 1.17 μM (in THP-1 cells), respectively, than compounds **1i**–**l**. These results implied that the antileukemia activities of the derivatives substituted with a methylenedioxy moiety at the C-7 and C-8 positions were greater than those of the derivatives substituted with methoxyl groups at the C-7 and C-8 positions. In other words, the antileukemia activities of the sanguinarine derivatives were significantly better than those of the chelerythrine derivatives. Therefore, it could be speculated that suitable nucleophilic groups, such as malonic esters and cyano, might enhance the antileukemia activity. Moreover, the substituents at the C-7 and C-8 positions were key units that affected the inhibitory activity of the compounds against the tested leukemia cell lines.

## 3. Materials and Methods

### 3.1. General Chemistry

Unless otherwise noted, all solvents and reagents were purchased from commercial sources, and some reactions were carried out under inert atmosphere and drying solvents with relevant specifications (extra dry, with molecular sieves, water ≤ 50 ppm (by K.F.) EnergySeal) purchased from commercial sources. All reactions were monitored by thin-layer chromatography (TLC) on silica gel GF_254_ plates (Qingdao Haiyang Chem. Ind. Ltd. P.R. Qingdao, China); spots were visualized with ultraviolet light (UV, Shanghai Jingke Ind. Co., Ltd., Shanghai, China) and 5% H_2_SO_4_ in ethanol. The following abbreviations are used: s = singlet, d = doublet, t = triplet, m = multiplet, and br.s = broad singlet. All first-order splitting patterns were assigned on the basis of appearance. All derivatives were purified by silica gel column chromatography. ^1^H- and ^13^C-NMR data were recorded with an INOVA-600 MHz spectrometer in CDCl_3_, CD_3_OD, acetone-*d_6_*, or DMSO-*d_6_* (Anhui Zesheng Tech. Co., Ltd., Hefei, Anhui, China) at room temperature, and the chemical shifts are shown relative to tetramethylsilane (TMS). High-resolution mass spectra were obtained using a Bruker microTOF-Q mass spectrometer.

### 3.2. Preparation of Raw Materials

Due to the low content of chelerythrine (**1**) and sanguinarine (**2**) in *Z. nitidum*, and it being difficult to enrich, we purchased the *Macleaya cordata* total alkaloids with higher content for enrichment and separation to obtain raw materials. TLC was used to identify the components of **1** and **2**. The gradient elution of petroleum ether/ethyl acetate system (*v*/*v*: 10:1→1:1) was carried out by silica gel column chromatography, and five fractions (Fr.1–Fr.5) were obtained. Fr.2–Fr.4 were separated and purified by repeated silica gel column chromatography (petroleum ether/ethyl acetate: 4/1, 2/1, 1/1; CH_3_Cl/CH_3_OH: 49/1, 20/1, etc.) to obtain these two raw materials. Their structure was further determined by ^1^H- and ^13^C-NMR (The ^1^H- and ^13^C-NMR and HPLC spectra of the two compounds S1–S4, S104–S105 are shown in the Supplementary Materials).

### 3.3. Procedure for Synthesizing Benzophenanthridine Alkaloid Derivatives

#### 3.3.1. Synthesis of Compounds **1a** and **2a**

Trimethylsilyl cyanlde (TMSCN) (200 μL, 0.194 mmol) and 4-dimethylaminopyridine (DMAP) (60 mg, 0.492 mmol) were added to a stirred solution of **1** or **2** (0.144 mmol) in dry dichloromethane (DCM) (10 mL) at room temperature, and the reaction mixture was stirred under reflux for 14 h. After the reaction was complete, the mixture was washed with saturated NaHCO_3_ solution three times and filtered. The filtrate was washed with an aqueous hydrochloric acid solution (0.1 mol/L, 5 × 10 mL), and then the organic layer was collected, dried over anhydrous Na_2_SO_4_, and concentrated under vacuum. The crude products were washed with methanol and filtered, and then dried under vacuum to obtain target compounds **1a** and **2a**.

Compound **1a**: light-yellow powder; yield: 65.1%; ^1^H-NMR (600 MHz, CDCl_3_) δ 7.74 (d, *J* = 8.6 Hz, 1H), 7.68 (s, 1H), 7.61 (d, *J* = 8.6 Hz, 1H), 7.58 (d, *J* = 8.5 Hz, 1H), 7.16 (s, 1H), 7.10 (d, *J* = 8.5 Hz, 1H), 6.09 (s, 2H), 5.67 (s, 1H), 4.03 (s, 3H), 3.98 (s, 3H), 2.65 (s, 3H); ^13^C-NMR (151 MHz, CDCl_3_) δ 152.37, 148.53, 147.97, 146.08, 138.61, 131.31, 126.66, 125.15, 125.05, 122.94, 120.72, 119.86, 119.37, 118.34, 113.50, 104.48, 101.22, 100.60, 61.29, 56.00, 48.58, 41.56. HR-ESI-MS (*m*/*z*) calculated for C_22_H_19_O_4_N_2_ [M + H]^+^ 375.1331, found 375.1339.

Compound **2a**: khaki powder; yield: 61.2%; ^1^H-NMR (600 MHz, CDCl_3_) δ 7.72 (d, J = 8.5 Hz, 1H), 7.68 (s, 1H), 7.58 (d, *J* = 8.5 Hz, 1H), 7.39 (d, *J* = 8.1 Hz, 1H), 7.15 (s, 1H), 6.98 (d, *J* = 8.2 Hz, 1H), 6.14 (dd, *J* = 15.9, 1.6 Hz, 2H), 6.10 (d, *J* = 1.9 Hz, 2H), 5.35 (s, 1H), 2.69 (s, 3H); ^13^C-NMR (151 MHz, CDCl_3_) δ 148.61, 148.02, 147.82, 145.03, 138.48, 131.28, 126.70, 125.92, 125.35, 123.15, 120.08, 117.39, 117.11, 109.41, 107.62, 104.47, 102.20, 101.26, 100.57, 48.75, 41.44. HR-ESI-MS (*m*/*z*) calculated for C_21_H_15_O_4_N_2_ [M + H]^+^ 359.1018, found 359.1026.

#### 3.3.2. Synthesis of Compounds **1b** and **2b**

NaBH_4_ (10 mg, 0.264 mmol) was added to a solution of **1** or **2** (0.052 mmol) in MeOH (5 mL) at room temperature. The reaction mixture was stirred for 0.5 h at the same temperature. After the reaction was complete, acetic acid was added to remove the excess NaBH_4_ and concentrated under vacuum. The residue was dissolved in dry DCM and extracted with saturated aqueous NaCl (3 × 10 mL). The organic layer was collected, dried over anhydrous Na_2_SO_4_, and concentrated under vacuum. Finally, the crude products were purified by silica gel column chromatography (petroleum ether (PE)/ethyl acetate (EA), 10:1) to obtain the target compounds.

Compound **1b**: light-yellow powder; yield: 89.7%; ^1^H-NMR (600 MHz, CDCl_3_) δ 7.73 (d, J = 8.5 Hz, 1H), 7.70 (s, 1H), 7.53 (d, *J* = 8.5 Hz, 1H), 7.50 (d, *J* = 8.4 Hz, 1H), 7.14 (s, 1H), 6.97 (d, *J* = 8.4 Hz, 1H), 6.07 (s, 2H), 4.32 (s, 2H), 3.95 (s, 3H), 3.90 (s, 3H), 2.62 (s, 3H); ^13^C-NMR (151 MHz, CDCl_3_) δ 152.30, 148.09, 147.49, 146.14, 142.75, 130.83, 126.41, 126.30, 126.27, 124.28, 123.80, 120.15, 118.70, 111.00, 104.37, 101.04, 100.75, 61.12, 55.84, 48.76, 41.32. HR-ESI-MS (*m*/*z*) calculated for C_21_H_20_O_4_N [M + H]^+^ 350.1381, found 350.1387.

Compound **2b**: white powder; yield: 94.7%; ^1^H-NMR (600 MHz, CDCl_3_) δ 7.71 (d, J = 8.0 Hz, 2H), 7.50 (d, J = 8.5 Hz, 1H), 7.32 (d, J = 8.1 Hz, 1H), 7.13 (s, 1H), 6.88 (d, J = 8.1 Hz, 1H), 6.07 (d, J = 7.7 Hz, 4H), 4.23 (s, 2H), 2.65 (s, 3H); ^13^C-NMR (151 MHz, CDCl_3_) δ 148.17, 147.54, 147.13, 144.66, 142.54, 130.84, 127.29, 126.55, 124.43, 123.97, 120.38, 116.22, 113.65, 107.21, 104.37, 101.35, 101.07, 100.77, 48.47, 41.59. HR-ESI-MS (*m*/*z*) calculated for C_20_H_16_O_4_N [M + H]^+^ 334.1069, found 334.1074.

#### 3.3.3. Synthesis of Compounds **1c**, **1d**, **1r**–**u,** and **2c**, **2d**

The indole compounds (two equivalents) were added to a solution of **1** or **2** (0.052 mmol) in CH_3_CN (10 mL) at room temperature. Each reaction mixture was stirred at the same temperature until the reaction was complete and then concentrated under vacuum. After that, the crude products were purified by silica gel column chromatography to obtain the target compounds.

Compound **1c**: The crude product was purified by silica gel column chromatography (PE/EA, 1.5:1) to obtain the target compound **1c**: white powder; yield: 43.5%; ^1^H-NMR (600 MHz, acetone-*d*_6_) δ 9.39 (s, 1H), 7.82–7.75 (m, 2H), 7.71 (d, *J* = 8.5 Hz, 1H), 7.66 (s, 1H), 7.62 (d, *J* = 2.4 Hz, 1H), 7.41 (d, *J* = 8.5 Hz, 1H), 7.17 (d, *J* = 8.6 Hz, 1H), 7.05 (s, 1H), 7.01 (d, *J* = 8.5 Hz, 1H), 6.63 (dd, *J* = 8.6, 2.4 Hz, 1H), 6.18 (q, *J* = 1.2 Hz, 1H), 6.05 (d, *J* = 1.0 Hz, 1H), 5.99 (d, *J* = 1.0 Hz, 1H), 5.96 (d, *J* = 1.1 Hz, 1H), 3.96 (s, 3H), 3.77 (s, 3H), 2.88 (s, 3H); ^13^C-NMR (151 MHz, Acetone-d_6_) δ 152.46, 150.59, 147.87, 147.38, 146.41, 141.07, 131.77, 131.00, 128.23, 128.14, 127.44, 125.62, 124.32, 123.92, 123.27, 119.77, 118.74, 115.88, 111.65, 111.41, 111.28, 104.30, 103.85, 101.06, 100.71, 60.13, 55.23, 54.52, 41.61. HR-ESI-MS (*m*/*z*) calculated for C_29_H_25_O_5_N_2_ [M + H]^+^ 481.1755, found 481.1758.

Compound **1d**: The crude product was purified by silica gel column chromatography (PE/EA, 4:1) to obtain the target compound **1d**: white powder; yield: 41.9%; ^1^H-NMR (600 MHz, DMSO-*d*_6_) δ 10.69 (d, *J* = 2.6 Hz, 1H), 8.05 (d, *J* = 2.1 Hz, 1H), 7.78 (d, *J* = 8.7 Hz, 1H), 7.72 (d, *J* = 8.6 Hz, 1H), 7.51 (s, 1H), 7.42 (d, *J* = 8.5 Hz, 1H), 7.20 (d, *J* = 8.6 Hz, 1H), 7.19–7.16 (m, 2H), 7.00 (dd, *J* = 8.6, 2.1 Hz, 1H), 6.22–6.20 (m, 1H), 6.07 (d, *J* = 6.0 Hz, 2H), 5.86 (d, *J* = 1.1 Hz, 1H), 3.91 (s, 3H), 3.72 (s, 3H), 2.78 (s, 3H); ^13^C-NMR (151 MHz, DMSO-*d*_6_) δ 152.47, 148.16, 147.52, 146.15, 140.73, 135.41, 130.91, 128.18, 127.46, 126.98, 125.42, 125.21, 124.20, 123.83, 123.50, 121.33, 120.29, 119.66, 119.57, 116.19, 113.43, 112.48, 104.53, 101.58, 100.26, 61.04, 56.17, 54.27, 42.32. HR-ESI-MS (*m*/*z*) calculated for C_29_H_24_O_3_N_2_Cl [M + H]^+^ 499.1413, found 499.1419.

Compound **1r**: The crude product was purified by silica gel column chromatography (PE/EA, 4:1) to obtain the target compound **1r**: white solid; yield: 52.5%; ^1^H-NMR (600 MHz, CDCl_3_) δ 8.18 (dd, *J* = 7.8, 1.1 Hz, 1H), 7.71 (d, *J* = 8.5 Hz, 1H), 7.68 (s, 1H), 7.63 (d, *J* = 8.6 Hz, 1H), 7.56 (s, 1H), 7.37 (d, *J* = 8.5 Hz, 1H), 7.20–7.18 (m, 1H), 7.17–7.15 (m, 1H), 7.11 (ddd, *J* = 8.1, 6.9, 1.2 Hz, 1H), 7.05 (d, *J* = 8.5 Hz, 1H), 6.98 (s, 1H), 6.26 (dd, *J* = 2.5, 1.1 Hz, 1H), 6.04 (d, *J* = 1.2 Hz, 1H), 6.00 (d, *J* = 1.4 Hz, 1H), 5.94 (d, *J* = 1.4 Hz, 1H), 3.98 (s, 3H), 3.80 (s, 3H), 2.89 (s, 3H); ^13^C-NMR (151 MHz, CDCl_3_) δ 152.23, 147.76, 147.26, 146.31, 141.13, 136.51, 130.89, 128.20, 127.46, 127.23, 125.85, 124.01, 123.30, 122.99, 121.65, 120.49, 119.64, 119.22, 118.95, 117.60, 111.35, 110.85, 104.08, 101.21, 100.81, 61.19, 55.83, 54.35, 42.32. HR-ESI-MS (*m*/*z*) calculated for C_29_H_23_O_4_N_2_ [M − H]^−^ 463.1646, found 463.1652.

Compound **1s**: The crude product was purified by silica gel column chromatography (PE/EA, 1:1) to obtain the target compound **1s**: brown powder; yield: 40.0%; ^1^H-NMR (600 MHz, DMSO-*d_6_*) δ 9.96 (d, *J* = 2.7 Hz, 1H), 7.80 (d, *J* = 8.5 Hz, 1H), 7.71 (d, *J* = 8.6 Hz, 1H), 7.53 (s, 1H), 7.42 (d, *J* = 8.5 Hz, 1H), 7.25 (d, *J* = 2.2 Hz, 1H), 7.18 (s, 1H), 7.15 (d, *J* = 8.6 Hz, 1H), 6.87 (d, *J* = 8.4 Hz, 1H), 6.42 (dd, *J* = 8.5, 2.2 Hz, 1H), 6.07 (s, 1H), 6.03 (s, 1H), 5.87 (d, *J* = 2.5 Hz, 1H), 5.74 (s, 1H), 3.90 (s, 3H), 3.67 (s, 3H), 2.77 (s, 3H); ^13^C-NMR (151 MHz, DMSO-*d*_6_) δ 152.40, 147.98, 147.44, 145.99, 141.23, 141.03, 130.88, 130.68, 128.27, 128.15, 127.20, 125.35, 124.30, 123.58, 123.41, 120.25, 119.33, 114.72, 112.28, 112.18, 111.83, 104.44, 103.52, 101.46, 100.71, 60.89, 56.12, 54.49, 42.54. HR-ESI-MS (*m*/*z*) calculated for C_29_H_26_O_4_N_3_ [M + H]^+^ 480.1914, found 480.1918.

Compound **1t**: The crude product was purified by silica gel column chromatography (PE/EA, 3:1) to obtain the target compound **1t**: white powder; yield: 36.4%; ^1^H-NMR (600 MHz, CDCl_3_) δ 8.00 (d, *J* = 7.9 Hz, 1H), 7.73–7.66 (m, 2H), 7.63 (d, *J* = 8.5 Hz, 1H), 7.33 (d, *J* = 8.5 Hz, 1H), 7.19 (s, 1H), 7.15–7.04 (m, 2H), 6.95 (s, 1H), 6.87 (d, *J* = 7.1 Hz, 1H), 6.06 (dd, *J* = 2.5, 1.2 Hz, 1H), 6.01 (d, *J* = 1.1 Hz, 1H), 5.99 (s, 1H), 5.92 (d, *J* = 1.4 Hz, 1H), 3.99 (s, 3H), 3.77 (s, 3H), 2.88 (s, 3H), 2.15 (s, 3H); ^13^C-NMR (151 MHz, CDCl_3_) δ 152.24, 147.79, 147.26, 146.31, 141.15, 136.01, 130.87, 128.34, 127.45, 126.62, 125.84, 124.04, 123.28, 122.83, 122.12, 119.96, 119.64, 119.35, 118.89, 118.08, 117.78, 111.33, 104.10, 101.19, 100.81, 61.19, 55.86, 54.36, 42.32, 16.22. HR-ESI-MS (*m*/*z*) calculated for C_30_H_27_O_4_N_2_ [M + H]^+^ 479.1959, found 479.1065.

Compound **1u**: The crude product was purified by silica gel column chromatography (PE/EA, 4:1) to obtain the target compound **1u**: white powder; yield: 43.3%; ^1^H-NMR (600 MHz, DMSO-*d*_6_) δ 10.59 (s, 1H), 7.78 (d, *J* = 8.7 Hz, 1H), 7.75–7.68 (m, 2H), 7.54 (s, 1H), 7.42 (d, *J* = 8.6 Hz, 1H), 7.17 (d, *J* = 8.4 Hz, 3H), 6.84 (td, *J* = 9.2, 2.6 Hz, 1H), 6.22 (d, *J* = 2.5 Hz, 1H), 6.07 (d, *J* = 11.6 Hz, 2H), 5.84 (s, 1H), 3.91 (s, 3H), 3.72 (s, 3H), 2.78 (s, 3H); ^13^C-NMR (151 MHz, DMSO-*d*_6_) δ 156.20, 152.46, 148.12, 147.51, 146.13, 140.80, 133.60, 130.91, 127.53, 127.01, 125.69, 125.24, 124.18, 123.77, 120.28, 119.56, 116.47, 112.82, 112.42, 109.61, 109.43, 104.90, 104.53, 101.56, 100.33, 61.02, 56.15, 54.35, 42.30. HR-ESI-MS (*m*/*z*) calculated for C_29_H_24_O_4_N_2_F [M + H]^+^ 483.1710, found 483.1715.

Compound **2c**: The crude product was purified by silica gel column chromatography (PE/EA, 1:1) to obtain the target compound **2c**: khaki powder; yield: 35.8%; ^1^H-NMR (600 MHz, CDCl_3_) δ 7.69 (d, *J* = 7.9 Hz, 2H), 7.52 (d, J = 2.5 Hz, 1H), 7.50 (s, 1H), 7.40 (dd, *J* = 9.7, 8.3 Hz, 2H), 7.02 (d, *J* = 9.2 Hz, 2H), 6.94 (d, *J* = 8.1 Hz, 1H), 6.70 (dd, *J* = 8.6, 2.5 Hz, 1H), 6.35–6.30 (m, 1H), 6.06 (dd, *J* = 9.4, 1.5 Hz, 2H), 6.01 (d, *J* = 1.4 Hz, 1H), 5.96 (d, *J* = 1.3 Hz, 1H), 5.73 (d, *J* = 1.1 Hz, 1H), 2.88 (s, 3H); ^13^C-NMR (151 MHz, CDCl_3_) δ 149.22, 147.89, 147.35, 147.07, 144.90, 140.92, 131.86, 130.91, 127.68, 127.53, 126.66, 125.24, 123.97, 123.57, 119.95, 116.58, 111.83, 111.60, 111.54, 107.50, 104.75, 104.16, 102.06, 101.42, 101.14, 100.89, 54.33, 42.70. HR-ESI-MS (*m*/*z*) calculated for C_28_H_21_O_5_N_2_ [M + H]^+^ 465.1441, found 465.1445.

Compound **2d**: The crude product was purified by silica gel column chromatography (PE/EA, 4:1) to obtain the target compound **2d**: white powder; yield: 27.5%; ^1^H-NMR (600 MHz, CDCl_3_) δ 8.09 (s, 1H), 7.70–7.66 (m, 2H), 7.64 (s, 1H), 7.41 (d, *J* = 2.1 Hz, 1H), 7.39 (d, *J* = 2.5 Hz, 1H), 7.06 (d, *J* = 1.5 Hz, 2H), 7.02 (s, 1H), 6.95 (d, *J* = 8.2 Hz, 1H), 6.38 (dd, *J* = 2.6, 1.2 Hz, 1H), 6.09 (q, *J* = 1.5 Hz, 2H), 6.01 (dd, *J* = 18.7, 1.3 Hz, 2H), 5.75 (d, *J* = 1.2 Hz, 1H), 2.89 (s, 3H); ^13^C-NMR (151 MHz, CDCl_3_) δ 148.09, 147.46, 147.14, 144.85, 140.72, 134.82, 130.90, 127.84, 127.40, 126.51, 125.08, 124.11, 123.95, 123.68, 122.11, 119.91, 119.63, 116.71, 116.37, 115.47, 111.92, 107.62, 104.15, 101.46, 100.97, 54.18, 42.70, 14.21. HR-ESI-MS (*m*/*z*) calculated for C_28_H_20_O_4_N_2_Cl [M + H]^+^ 483.1102, found 483.1106.

#### 3.3.4. Synthesis of Compounds **1e** and **2e**

Ethyl trimethylsilyl acetate (24 mg, 0.164 mmol) and CsF (23 mg, 0.151 mmol) were added to a stirred solution of **1** or **2** (0.052 mmol) in CH_3_CN (15 mL) at room temperature. Each reaction mixture was stirred for 4–5 h at the same temperature until the reaction was complete and then concentrated under vacuum. After that, the crude products were purified by silica gel column chromatography to obtain the target products.

Compound **1e**: The crude product was purified by silica gel column chromatography (PE/EA, 4:1) to obtain the target compound **1e**: white solid; yield: 52.0%; ^1^H-NMR (600 MHz, CDCl_3_) δ 7.73 (d, *J* = 8.6 Hz, 1H), 7.59–7.55 (m, 2H), 7.50 (d, *J* = 8.4 Hz, 1H), 7.12 (s, 1H), 6.99 (d, *J* = 8.5 Hz, 1H), 6.06 (s, 2H), 5.03 (dd, *J* = 11.1, 4.4 Hz, 1H), 4.22–4.13 (m, 2H), 3.99 (s, 3H), 3.95 (s, 3H), 2.67 (s, 3H), 2.42–2.29 (m, 2H), 1.21 (t, J = 7.2 Hz, 3H); ^13^C-NMR (151 MHz, CDCl_3_) δ 171.70, 152.11, 147.96, 147.51, 145.76, 139.40, 131.07, 127.96, 127.56, 124.92, 123.80, 123.09, 119.76, 118.81, 111.61, 104.30, 100.99, 100.94, 61.04, 60.27, 55.83, 55.10, 42.88, 39.19, 14.23. HR-ESI-MS (*m*/*z*) calculated for C_25_H_25_O_6_N Na [M + Na]^+^ 458.1569, found 458.1574.

Compound **2e**: The crude product was purified by silica gel column chromatography (PE/EA, 6:1) to obtain the target compound **2e**: white powder; yield: 47.5%; ^1^H-NMR (600 MHz, CDCl_3_) δ 7.71 (d, *J* = 8.6 Hz, 1H), 7.57 (s, 1H), 7.50 (d, *J* = 8.5 Hz, 1H), 7.37 (d, *J* = 8.2 Hz, 1H), 7.12 (s, 1H), 6.89 (d, *J* = 8.1 Hz, 1H), 6.13–6.01 (m, 4H), 4.85 (dd, *J* = 8.5, 6.9 Hz, 1H), 4.18 (m, *J* = 10.8, 7.2 Hz, 2H), 2.68 (s, 3H), 2.41 (d, *J* = 7.8 Hz, 2H), 1.23 (t, *J* = 7.2 Hz, 3H); ^13^C-NMR (151 MHz, CDCl_3_) δ 171.40, 148.07, 147.57, 147.11, 144.48, 139.27, 131.06, 127.67, 125.81, 124.01, 123.24, 119.99, 116.46, 115.73, 107.68, 104.30, 101.56, 101.02, 100.91, 60.41, 54.81, 43.14, 39.00, 14.25. HR-ESI-MS (*m*/*z*) calculated for C_24_H_21_O_6_N Na [M + Na]^+^ 442.1257, found 442.1261.

#### 3.3.5. Synthesis of Compounds **1f** and **2f**

Compound **1e** or **2e** (0.043 mmol) in dry tetrahydrofuran (THF, 5 mL) was cooled to 5 °C. After 5 min, LiAlH_4_ (0.75 mmol, 300 μL) was slowly added to the mixture under an argon atmosphere, followed by stirring for 0.5 h at 5 °C. After the reaction was complete, it was quenched with a 15% NaOH aqueous solution and extracted with DCM (3 × 5 mL). The combined organic layers were collected, dried over anhydrous Na_2_SO_4_, and concentrated under vacuum. After that, the crude products were purified by silica gel column chromatography to obtain the target compounds.

Compound **1f**: The crude product was purified by silica gel column chromatography (PE/EA, 2:1) to obtain the target compound **1f**: white solid; yield: 73.8%; ^1^H-NMR (600 MHz, CDCl_3_) δ 7.73 (d, *J* = 8.6 Hz, 1H), 7.58 (s, 1H), 7.56 (d, *J* = 8.5 Hz, 1H), 7.52 (d, *J* = 8.5 Hz, 1H), 7.14 (s, 1H), 6.98 (d, *J* = 8.5 Hz, 1H), 6.06 (dd, *J* = 12.0, 1.4 Hz, 2H), 4.68 (dd, *J* = 9.4, 5.4 Hz, 1H), 3.97 (s, 3H), 3.96 (s, 3H), 3.79 (ddd, *J* = 11.2, 9.3, 3.5 Hz, 1H), 3.71 (dt, *J* = 10.9, 4.6 Hz, 1H), 2.70 (s, 3H), 1.81 (dtd, *J* = 14.0, 9.4, 4.5 Hz, 1H), 1.51 (dtd, *J* = 14.1, 5.1, 3.4 Hz, 1H); ^13^C-NMR (151 MHz, CDCl_3_) δ 152.13, 148.47, 147.59, 145.69, 139.20, 131.11, 128.74, 127.06, 124.62, 124.23, 123.80, 119.89, 119.26, 111.33, 104.63, 101.14, 99.95, 61.76, 61.14, 57.14, 55.81, 42.63, 35.47. HR-ESI-MS (*m*/*z*) calculated for C_23_H_24_O_5_N [M + H]^+^ 394.1643, found 394.1649.

Compound **2f**: The crude product was purified by silica gel column chromatography (PE/EA, 4:1) to obtain the target compound **2f**: white solid; yield: 70.5%; ^1^H-NMR (600 MHz, CDCl_3_) δ 7.71 (d, *J* = 8.5 Hz, 1H), 7.57 (s, 1H), 7.51 (d, *J* = 8.5 Hz, 1H), 7.35 (d, *J* = 8.1 Hz, 1H), 7.13 (s, 1H), 6.88 (d, *J* = 8.1 Hz, 1H), 6.07 (dd, *J* = 3.1, 1.5 Hz, 2H), 6.05 (dd, *J* = 3.3, 1.5 Hz, 2H), 4.50 (dd, *J* = 10.0, 4.9 Hz, 1H), 3.87–3.78 (m, 3H), 2.71 (s, 3H), 1.85–1.76 (m, 1H), 1.58 (dq, J = 14.5, 4.8 Hz, 1H); ^13^C-NMR (151 MHz, CDCl_3_) δ 148.52, 147.61, 147.10, 144.34, 139.01, 131.09, 127.18, 125.55, 124.38, 123.90, 120.13, 116.91, 116.71, 107.50, 104.62, 101.47, 101.16, 99.93, 61.58, 56.81, 43.08, 35.20. HR-ESI-MS (*m*/*z*) calculated for C_22_H_20_O_5_N [M + H]^+^ 378.1331, found 378.1336.

#### 3.3.6. Synthesis of Compounds **1g**, **1h**, **2g** and **2h**

RMgBr (1.0 mol/L, 1.5 equivalents) was added to a solution of **1** or **2** (0.052 mmol) in dry THF (10 mL) under an argon atmosphere at room temperature. The reaction mixture was stirred at the same temperature until the reaction was complete and then concentrated under vacuum. After that, the crude products were purified by silica gel column chromatography to obtain the target compounds.

Compound **1g**: white solid; yield: 80.5%; the crude product was purified by silica gel column chromatography (PE/EA, 49:1) to obtain the pure compound **1g**. ^1^H-NMR (600 MHz, CDCl_3_) δ 7.77–7.70 (m, 2H), 7.57 (d, *J* = 8.5 Hz, 1H), 7.50 (d, *J* = 8.5 Hz, 1H), 7.14 (s, 1H), 6.97 (d, *J* = 8.5 Hz, 1H), 6.08–6.07 (m, 2H), 6.06–5.99 (m, 1H), 4.99 (ddd, *J* = 10.2, 2.2, 1.1 Hz, 1H), 4.88 (dq, *J* = 17.2, 1.4 Hz, 1H), 4.52 (dd, *J* = 9.7, 5.3 Hz, 1H), 3.97 (s, 3H), 3.95 (s, 3H), 2.66 (s, 3H), 2.28–2.05 (m, 2H); ^13^C-NMR (151 MHz, CDCl_3_) δ 152.14, 147.95, 147.45, 145.83, 140.06, 136.37, 130.99, 129.74, 127.54, 124.78, 123.68, 123.53, 119.87, 118.84, 115.78, 111.09, 104.32, 100.99, 100.73, 61.02, 58.23, 55.78, 42.71, 38.47. HR-ESI-MS (*m*/*z*) calculated for C_24_H_24_O_4_N [M + H]^+^ 390.1694, found 390.1700.

Compound **1h**: white solid; yield: 78.9%; the crude product was purified by silica gel column chromatography (PE/EA, 47:1) to obtain the target compound **1h**. ^1^H-NMR (600 MHz, CDCl_3_) δ 7.78 (s, 1H), 7.71 (d, *J* = 8.5 Hz, 1H), 7.57 (d, *J* = 8.5 Hz, 1H), 7.48 (d, *J* = 8.5 Hz, 1H), 7.12 (s, 1H), 7.00 (d, *J* = 8.6 Hz, 1H), 6.07 (s, 2H), 5.83 (ddd, *J* = 17.2, 10.4, 4.5 Hz, 1H), 5.13 (dt, *J* = 4.2, 2.0 Hz, 1H), 4.94–4.80 (m, 2H), 3.96 (s, 3H), 3.95 (s, 3H), 2.69 (s, 3H); ^13^C-NMR (151 MHz, CDCl_3_) δ 152.16, 148.08, 147.45, 146.31, 140.61, 138.12, 130.89, 127.79, 127.23, 125.18, 123.78, 123.65, 119.87, 118.93, 115.15, 111.38, 104.41, 101.02, 100.78, 60.97, 59.32, 55.80, 42.38. HR-ESI-MS (*m*/*z*) calculated for C_23_H_22_O_4_N [M + H]^+^ 376.1539, found 376.1543.

Compound **2g**: white solid; yield: 80.1%; the crude product was purified by silica gel column chromatography (PE/EA, 49:1) to obtain the target compound **2g**. ^1^H-NMR (600 MHz, CDCl_3_) δ 7.72 (d, *J* = 8.2 Hz, 2H), 7.50 (d, *J* = 8.5 Hz, 1H), 7.36 (d, *J* = 8.1 Hz, 1H), 7.13 (s, 1H), 6.88 (d, *J* = 8.1 Hz, 1H), 6.10–6.03 (m, 4H), 5.98 (ddt, *J* = 17.2, 10.2, 7.0 Hz, 1H), 5.05–4.98 (m, 1H), 4.92 (dq, *J* = 17.2, 1.6 Hz, 1H), 4.30 (dd, *J* = 9.1, 6.0 Hz, 1H), 2.67 (s, 3H), 2.20 (ddt, *J* = 50.5, 14.3, 7.4 Hz, 2H); ^13^C-NMR (151 MHz, CDCl_3_) δ 148.04, 147.50, 146.95, 144.61, 139.99, 135.72, 130.96, 127.65, 125.74, 123.73, 123.72, 120.10, 117.46, 116.44, 116.27, 107.24, 104.31, 101.32, 101.01, 100.98, 58.13, 43.03, 38.32. HR-ESI-MS (*m*/*z*) calculated for C_23_H_20_O_4_N [M + H]^+^ 374.1384, found 374.1387.

Compound **2h**: white solid; yield: 78.6%; the crude product was purified by silica gel column chromatography (PE/EA, 49:1) to obtain the target compound **2h**. ^1^H-NMR (600 MHz, CDCl_3_) δ 7.78 (s, 1H), 7.70 (d, *J* = 8.6 Hz, 1H), 7.48 (d, *J* = 8.5 Hz, 1H), 7.37 (d, *J* = 8.1 Hz, 1H), 7.13 (s, 1H), 6.90 (d, *J* = 8.1 Hz, 1H), 6.16–6.03 (m, 4H), 5.91–5.76 (m, 1H), 5.02–4.91 (m, 1H), 4.93–4.86 (m, 2H), 2.72 (s, 3H); ^13^C-NMR (151 MHz, CDCl_3_) δ 148.18, 147.51, 147.02, 145.08, 140.41, 137.12, 130.90, 127.38, 126.07, 123.89, 123.86, 120.10, 116.52, 115.57, 115.29, 107.52, 104.40, 101.47, 101.05, 100.79, 59.34, 42.77. HR-ESI-MS (*m*/*z*) calculated for C_22_H_18_O_4_N [M + H]^+^ 360.1225, found 360.1230.

#### 3.3.7. Synthesis of Compounds **1i**–**l** and **2i**–**l**

Malonate diester compounds (1.5 equivalents) were added to a solution of **1** or **2** (0.115 mmol) in CH_3_CN (20 mL) at room temperature. The reaction mixture was stirred for 5–14 h at the same temperature until the reaction was complete and then concentrated under vacuum. After that, the crude products were purified by silica gel column chromatography (PE/EA, 4:1) to obtain the target products.

Compound **1i**: white solid; yield: 36.3%; ^1^H-NMR (600 MHz, CDCl_3_) δ 7.76 (d, *J* = 8.6 Hz, 1H), 7.57 (d, *J* = 8.5 Hz, 1H), 7.51 (d, *J* = 8.5 Hz, 1H), 7.44 (s, 1H), 7.13 (s, 1H), 7.02 (d, *J* = 8.5 Hz, 1H), 6.06 (dd, *J* = 8.6, 1.4 Hz, 2H), 5.24 (d, *J* = 10.8 Hz, 1H), 3.94 (s, 3H), 3.94 (s, 3H), 3.66 (s, 3H), 3.59 (s, 3H), 3.40 (d, *J* = 10.9 Hz, 1H), 2.71 (s, 3H); ^13^C-NMR (151 MHz, CDCl_3_) δ 168.39, 167.22, 151.93, 148.10, 147.56, 146.81, 138.45, 131.07, 127.11, 125.08, 124.31, 124.02, 123.26, 119.81, 118.97, 112.45, 104.45, 101.06, 100.60, 61.01, 57.56, 55.91, 55.28, 52.43, 52.10, 42.22. HR-ESI-MS (*m*/*z*) calculated for C_26_H_26_O_8_N [M + H]^+^ 480.1643, found 480.1653.

Compound **1j**: white solid; yield: 30.9%; ^1^H-NMR (600 MHz, CDCl_3_) δ 7.76 (d, *J* = 8.5 Hz, 1H), 7.56 (d, *J* = 8.5 Hz, 1H), 7.50 (d, *J* = 8.5 Hz, 1H), 7.46 (s, 1H), 7.12 (d, *J* = 2.2 Hz, 1H), 7.01 (d, *J* = 8.5 Hz, 1H), 6.05 (s, 2H), 5.25 (d, *J* = 10.8 Hz, 1H), 4.25–4.11 (m, 2H), 4.05–3.96 (m, 2H), 3.94 (s, 3H), 3.93 (s, 3H), 3.37 (d, *J* = 10.8 Hz, 1H), 2.72 (s, 3H), 1.12 (t, *J* = 7.1 Hz, 3H), 1.04 (t, *J* = 7.2 Hz, 3H); ^13^C-NMR (151 MHz, CDCl_3_) δ 168.03, 167.03, 151.92, 148.01, 147.50, 146.87, 138.62, 131.05, 127.17, 125.14, 124.53, 123.92, 123.37, 119.81, 118.85, 112.15, 104.41, 101.04, 100.76, 61.25, 61.01, 60.96, 57.35, 55.82, 55.33, 42.11, 13.96, 13.64. HR-ESI-MS (*m*/*z*) calculated for C_28_H_29_O_8_N Na [M + Na]^+^ 530.1776, found 530.1785.

Compound **1k**: white solid; yield: 30.9%; ^1^H-NMR (600 MHz, CDCl_3_) δ 7.76 (d, *J* = 8.5 Hz, 1H), 7.55 (d, *J* = 8.5 Hz, 1H), 7.52–7.45 (m, 2H), 7.12 (s, 1H), 6.99 (d, *J* = 8.5 Hz, 1H), 6.04 (s, 2H), 5.27 (d, *J* = 10.9 Hz, 1H), 5.11 (h, *J* = 6.3 Hz, 1H), 4.84 (p, *J* = 6.3 Hz, 1H), 3.94 (s, 3H), 3.92 (s, 3H), 3.32 (d, *J* = 10.9 Hz, 1H), 2.71 (s, 3H), 1.24 (d, *J* = 6.2 Hz, 3H), 1.11 (dd, *J* = 9.0, 6.3 Hz, 6H), 0.95 (d, *J* = 6.4 Hz, 3H); ^13^C-NMR (151 MHz, CDCl_3_) δ 167.65, 166.42, 151.96, 147.98, 147.48, 146.92, 138.71, 131.04, 127.22, 125.24, 124.50, 123.82, 123.52, 119.82, 118.76, 112.00, 104.38, 101.00, 100.90, 68.59, 68.40, 60.85, 56.91, 55.76, 55.48, 41.95, 21.68, 21.62, 21.57, 21.08. HR-ESI-MS (*m*/*z*) calculated for C_30_H_33_O_8_N Na [M + Na]^+^ 558.2089, found 558.2098.

Compound **1l**: white solid; yield: 38.6%; ^1^H-NMR (600 MHz, CDCl_3_) δ 7.76 (d, *J* = 8.6 Hz, 1H), 7.55 (d, *J* = 8.5 Hz, 1H), 7.50 (d, *J* = 8.5 Hz, 1H), 7.46 (s, 1H), 7.12 (s, 1H), 7.00 (d, *J* = 8.6 Hz, 1H), 6.05 (dd, *J* = 8.1, 1.4 Hz, 2H), 5.25 (d, *J* = 10.8 Hz, 1H), 4.17 (t, *J* = 6.7 Hz, 2H), 4.09 (qt, *J* = 10.7, 6.8 Hz, 2H), 3.94 (s, 3H), 3.92 (s, 3H), 3.39 (d, *J* = 1.9 Hz, 1H), 2.71 (s, 3H), 1.47–1.35 (m, 4H), 1.27–1.11 (m, 4H), 0.85 (dt, *J* = 12.9, 7.4 Hz, 6H); ^13^C-NMR (151 MHz, CDCl_3_) δ 168.09, 167.05, 151.95, 148.02, 147.50, 146.82, 138.65, 131.03, 127.19, 125.14, 124.54, 123.90, 123.41, 119.80, 118.84, 112.06, 104.38, 101.02, 100.79, 65.40, 64.97, 60.95, 57.28, 55.74, 55.31, 42.06, 30.45, 30.21, 19.03, 18.90, 13.70, 13.67. HR-ESI-MS (*m*/*z*) calculated for C_32_H_37_O_8_N Na [M + Na]^+^ 586.2406, found 586.2411.

Compound **2i**: light-orange solid; yield: 35.9%; ^1^H-NMR (600 MHz, CDCl_3_) δ 7.73 (d, *J* = 8.6 Hz, 1H), 7.52 (d, *J* = 8.5 Hz, 1H), 7.42 (s, 1H), 7.37 (d, *J* = 8.3 Hz, 1H), 7.13 (s, 1H), 6.92 (d, *J* = 8.1 Hz, 1H), 6.09–6.01 (m, 4H), 5.12 (d, *J* = 11.1 Hz, 1H), 3.70 (s, 3H), 3.61 (s, 3H), 3.47 (d, *J* = 11.1 Hz, 1H), 2.69 (s, 3H); ^13^C-NMR (151 MHz, CDCl_3_) δ 167.98, 167.01, 148.16, 147.62, 147.13, 145.35, 138.40, 131.08, 124.48, 124.27, 123.99, 123.34, 120.00, 117.03, 112.39, 108.33, 104.43, 101.51, 101.08, 100.57, 57.33, 55.16, 52.40, 52.31, 42.38. HR-ESI-MS (*m*/*z*) calculated for C_25_H_21_O_8_N Na [M + Na]^+^ 486.1150, found 486.1159.

Compound **2j**: white solid; yield: 33.8%; ^1^H-NMR (600 MHz, CDCl_3_) δ 7.74 (d, J = 8.6 Hz, 1H), 7.51 (d, *J* = 8.5 Hz, 1H), 7.45 (s, 1H), 7.36 (d, *J* = 8.1 Hz, 1H), 7.12 (s, 1H), 6.91 (d, J = 8.1 Hz, 1H), 6.09–5.97 (m, 4H), 5.13 (d, *J* = 11.1 Hz, 1H), 4.21 (ddq, *J* = 40.9, 10.7, 7.1 Hz, 2H), 4.11–3.90 (m, 2H), 3.43 (d, *J* = 11.1 Hz, 1H), 2.69 (s, 3H), 1.16 (t, *J* = 7.1 Hz, 3H), 1.11 (t, *J* = 7.1 Hz, 3H); ^13^C-NMR (151 MHz, CDCl_3_) δ 167.58, 166.80, 148.08, 147.57, 147.12, 145.40, 138.57, 131.07, 127.26, 126.10, 124.20, 123.44, 120.01, 116.94, 112.57, 108.18, 104.41, 101.44, 101.07, 100.72, 61.34, 61.29, 57.14, 55.33, 42.28, 13.99, 13.77. HR-ESI-MS (*m*/*z*) calculated for C_27_H_25_O_8_N Na [M + Na]^+^ 514.1461, found 514.1472.

Compound **2k**: white solid; yield: 25.6%; ^1^H-NMR (600 MHz, CDCl_3_) δ 7.74 (d, *J* = 8.6 Hz, 1H), 7.51 (d, *J* = 8.5 Hz, 1H), 7.46 (s, 1H), 7.35 (d, *J* = 8.1 Hz, 1H), 7.12 (s, 1H), 6.90 (d, *J* = 8.1 Hz, 1H), 6.12–5.76 (m, 4H), 5.17–5.06 (m, 2H), 4.91–4.76 (m, 1H), 3.38 (d, *J* = 11.1 Hz, 1H), 2.69 (s, 3H), 1.29 (d, *J* = 6.3 Hz, 3H), 1.16 (d, *J* = 6.3 Hz, 3H), 1.13 (d, *J* = 6.2 Hz, 3H), 1.00 (d, *J* = 6.2 Hz, 3H); ^13^C-NMR (151 MHz, CDCl_3_) δ 167.03, 166.35, 148.04, 147.55, 147.14, 145.47, 138.65, 131.06, 127.26, 126.18, 124.11, 123.53, 119.99, 116.87, 112.58, 108.04, 104.37, 101.36, 101.03, 100.87, 68.80, 68.70, 56.71, 55.59, 42.14, 21.76, 21.61, 21.47, 21.23. HR-ESI-MS (*m*/*z*) calculated for C_29_H_29_O_8_N Na [M + Na]^+^ 542.1781, found 542.1785.

Compound **2l**: white solid; yield: 28.3%; ^1^H-NMR (600 MHz, CDCl_3_) δ 7.74 (d, *J* = 8.6 Hz, 1H), 7.51 (d, *J* = 8.5 Hz, 1H), 7.45 (s, 1H), 7.36 (d, *J* = 8.1 Hz, 1H), 7.12 (s, 1H), 6.91 (d, *J* = 8.1 Hz, 1H), 6.11–5.99 (m, 4H), 5.12 (d, *J* = 11.1 Hz, 1H), 4.18 (dt, *J* = 10.9, 6.7 Hz, 1H), 4.10 (dt, *J* = 10.7, 6.8 Hz, 1H), 4.03–3.90 (m, 2H), 3.43 (d, *J* = 3.2 Hz, 1H), 2.69 (s, 3H), 1.55–1.38 (m, 4H), 1.30–1.23 (m, 2H), 1.22–1.14 (m, 2H), 0.89–0.84 (m, 6H); ^13^C-NMR (151 MHz, CDCl_3_) δ 167.59, 166.85, 148.08, 147.56, 147.16, 145.39, 138.59, 131.05, 127.26, 126.09, 124.18, 123.44, 119.98, 116.94, 112.58, 108.15, 104.37, 101.45, 101.05, 100.74, 65.23, 65.19, 57.07, 55.34, 42.24, 30.47, 30.26, 18.98, 18.87, 13.66, 13.63. HR-ESI-MS (*m*/*z*) calculated for C_31_H_33_O_8_N Na [M + Na]^+^ 570.2095, found 570.2098.

#### 3.3.8. Synthesis of Compound **1m**

To a stirred solution of **1** (100 mg, 0.287 mmol) in acetone (100 mL) was added a 20% solution of Na_2_CO_3_ in water at room temperature. The reaction mixture was stirred under reflux for 24 h. After the reaction was complete, the mixture was concentrated under vacuum. The residue was dissolved in DCM and extracted with saturated aqueous NaCl (3 × 10 mL). The combined organic layers were collected, dried over anhydrous Na_2_SO_4_, and concentrated under vacuum. After that, the crude product was purified by silica gel column chromatography (PE/EA, 4:1) to obtain the target compound.

Compound **1m**: white solid; yield: 90.2%; ^1^H-NMR (600 MHz, CDCl3) δ 7.73 (d, *J* = 8.6 Hz, 1H), 7.57 (d, *J* = 8.5 Hz, 1H), 7.54 (s, 1H), 7.51 (d, *J* = 8.5 Hz, 1H), 7.13 (s, 1H), 6.98 (d, *J* = 8.5 Hz, 1H), 6.11–6.04 (m, 2H), 5.07 (dd, *J* = 11.2, 3.7 Hz, 1H), 3.98 (s, 3H), 3.95 (s, 3H), 2.66 (s, 3H), 2.60 (dd, *J* = 15.0, 11.2 Hz, 1H), 2.28 (dd, *J* = 15.0, 3.7 Hz, 1H), 2.09 (s, 3H); ^13^C-NMR (151 MHz, CDCl3) δ 194.64, 145.84, 142.33, 141.82, 140.00, 134.55, 127.31, 124.79, 124.05, 121.81, 120.98, 120.48, 117.40, 116.55, 110.13, 103.85, 100.94, 100.57, 65.75, 61.19, 60.40, 53.31, 49.78, 39.52. HR-ESI-MS (*m*/*z*) calculated for C_24_H_23_O_5_N Na [M + Na]^+^ 428.1465, found 428.1468.

#### 3.3.9. Synthesis of Compounds **1n**–**q**

Aromatic aldehydes (3 equivalents), benzoic acid (30 mg, 0.246 mmol), and piperidine (300 μL) were added to a stirred solution of **1m** (30 mg, 0.074 mmol) in toluene (10 mL) at room temperature. The reaction mixture was stirred under reflux until the reaction was complete and then concentrated under vacuum. After that, the crude products were purified by silica gel column chromatography to obtain the target compounds.

Compound **1n**: The crude product was purified by silica gel column chromatography (PE/EA, 7:1) to obtain the target compound **1n**: yellow solid; yield: 62.0%; ^1^H-NMR (600 MHz, CDCl_3_) δ 7.79 (d, *J* = 8.6 Hz, 1H), 7.61 (d, *J* = 8.5 Hz, 1H), 7.53 (d, *J* = 8.5 Hz, 1H), 7.36 (s, 1H), 7.19 (t, *J* = 7.8 Hz, 1H), 7.01 (d, *J* = 7.8 Hz, 2H), 6.92–6.86 (m, 2H), 6.70–6.65 (m, 1H), 6.63 (t, *J* = 2.0 Hz, 1H), 6.48 (d, *J* = 16.2 Hz, 1H), 5.90 (d, *J* = 1.5 Hz, 1H), 5.63 (d, *J* = 1.6 Hz, 1H), 5.14 (dd, *J* = 11.4, 4.1 Hz, 1H), 4.02 (s, 3H), 3.96 (s, 3H), 3.80 (s, 3H), 2.98 (dd, *J* = 13.4, 11.4 Hz, 1H), 2.63 (s, 3H), 2.41 (dd, *J* = 13.5, 4.1 Hz, 1H); ^13^C-NMR (151 MHz, CDCl_3_) δ 199.99, 159.58, 152.24, 148.01, 147.41, 145.64, 142.97, 139.42, 135.85, 131.05, 129.42, 128.51, 127.68, 127.15, 124.86, 123.84, 123.37, 121.06, 119.79, 118.87, 116.16, 112.57, 111.60, 103.97, 101.50, 100.93, 61.13, 56.81, 55.85, 55.23, 42.94, 42.74. HR-ESI-MS (*m*/*z*) calculated for C_32_H_29_O_6_N Na [M + Na]^+^ 546.1876, found 546.1887.

Compound **1o**: The crude product was purified by silica gel column chromatography (PE/EA, 8:1) to obtain the target compound **1o**: yellow liquid; yield: 58.6%; ^1^H-NMR (600 MHz, CDCl_3_) δ 7.80 (d, *J* = 8.5 Hz, 1H), 7.61 (d, *J* = 8.5 Hz, 1H), 7.55 (d, *J* = 8.5 Hz, 1H), 7.36 (s, 1H), 7.18 (t, *J* = 7.5 Hz, 1H), 7.14 (d, *J* = 7.6 Hz, 1H), 7.05 (s, 1H), 7.01 (d, *J* = 8.5 Hz, 1H), 6.96 (d, *J* = 7.6 Hz, 1H), 6.90 (d, *J* = 16.3 Hz, 1H), 6.77 (s, 1H), 6.47 (d, *J* = 16.3 Hz, 1H), 5.88 (d, *J* = 1.6 Hz, 1H), 5.54 (d, *J* = 1.6 Hz, 1H), 5.14 (dd, *J* = 11.4, 4.0 Hz, 1H), 4.02 (s,3H), 3.97 (s, 3H), 2.97 (dd, *J* = 13.4, 11.4 Hz, 1H), 2.63 (s, 3H), 2.39 (dd, *J* = 13.4, 4.1 Hz, 1H), 2.32 (s, 3H); ^13^C-NMR (151 MHz, CDCl_3_) δ 200.01, 152.25, 148.00, 147.40, 145.65, 143.28, 139.47, 138.08, 134.43, 131.05, 130.76, 128.96, 128.59, 128.37, 127.35, 127.17, 125.25, 124.87, 123.85, 123.40, 119.82, 118.88, 111.59, 103.87, 101.56, 100.88, 61.14, 56.83, 55.85, 42.97, 42.72, 21.16. HR-ESI-MS (*m*/*z*) calculated for C_32_H_29_O_5_N Na [M + Na]^+^ 530.1925, found 530.1938.

Compound **1p**: The crude product was purified by silica gel column chromatography (PE/EA, 8:1) to obtain the target compound **1p**: yellow liquid; yield: 47.2%; ^1^H-NMR (600 MHz, CDCl_3_) δ 7.80 (d, *J* = 8.6 Hz, 1H), 7.61 (d, *J* = 8.5 Hz, 1H), 7.55 (d, *J* = 8.6 Hz, 1H), 7.44 (ddd, *J* = 8.0, 2.0, 1.0 Hz, 1H), 7.32 (s, 1H), 7.16 (d, *J* = 7.8 Hz, 1H), 7.08 (d, *J* = 1.8 Hz, 1H), 7.06 (s, 1H), 7.02 (dd, *J* = 8.8, 2.0 Hz, 2H), 6.76 (d, *J* = 16.3 Hz, 1H), 6.43 (d, *J* = 16.3 Hz, 1H), 5.91 (d, *J* = 1.6 Hz, 1H), 5.68 (d, *J* = 1.6 Hz, 1H), 5.12 (dd, *J* = 11.4, 4.3 Hz, 1H), 4.02 (s, 3H), 3.97 (s, 3H), 2.98 (dd, *J* = 13.2, 11.4 Hz, 1H), 2.62 (s, 3H), 2.39 (dd, *J* = 13.3, 4.2 Hz, 1H); ^13^C-NMR (151 MHz, CDCl_3_) δ 199.84, 152.27, 148.02, 147.35, 145.63, 141.19, 139.30, 136.59, 132.61, 131.09, 130.80, 129.91, 128.62, 128.37, 127.00, 126.62, 124.78, 123.96, 123.42, 122.61, 119.86, 118.94, 111.65, 104.11, 101.35, 101.00, 61.15, 57.03, 55.85, 42.90, 42.71. HR-ESI-MS (*m*/*z*) calculated for C_31_H_27_O_5_NBr [M + Na]^+^ 572.1057, found 572.1067.

Compound **1q**: The crude product was purified by silica gel column chromatography (PE/EA, 6:1) to obtain the target compound **1q**: yellow solid; yield: 29.9%; ^1^H-NMR (600 MHz, CDCl_3_) δ 7.77 (d, *J* = 8.5 Hz, 1H), 7.60 (d, *J* = 8.5 Hz, 1H), 7.50 (d, *J* = 8.4 Hz, 1H), 7.33 (s, 1H), 7.17–7.12 (m, 1H), 7.01 (d, *J* = 8.5 Hz, 1H), 6.95 (s, 1H), 6.93 (s, 1H), 6.85 (s, 1H), 6.38 (d, J = 16.2 Hz, 1H), 5.89 (d, *J* = 1.6 Hz, 1H), 5.72 (d, *J* = 1.6 Hz, 1H), 5.15 (dd, *J* = 11.3, 4.2 Hz, 1H), 4.03 (s, 3H), 3.97 (s, 3H), 3.91 (s, 3H), 3.87 (s, 3H), 3.06 (dd, *J* = 13.3, 11.3 Hz, 1H), 2.63 (s, 3H), 2.42 (dd, *J* = 13.4, 4.2 Hz, 1H); ^13^C-NMR (151 MHz, CDCl_3_) δ 200.08, 152.23, 151.09, 148.21, 147.70, 147.10, 145.69, 141.30, 139.29, 131.23, 128.44, 127.80, 127.03, 126.26, 124.93, 124.18, 123.42, 119.77, 118.88, 117.79, 115.28, 111.63, 109.18, 104.10, 101.47, 100.96, 61.15, 56.96, 56.32, 55.87, 42.76, 31.45, 30.21; HR-ESI-MS (*m*/*z*) calculated for C_33_H_30_O_7_NBr Na [M + Na]^+^ 654.1085, found 654.1098.

### 3.4. Biological Evaluations

#### 3.4.1. Cytotoxic Bioassay

According to the literature [33,34,35,36,37,38], the CCK-8 assay was used to determine cell (National Collection of Authenticated Cell Cultures, Shanghai, China) viability, and the cell survival rate was calculated according to optical density (OD) measurements. Single-cell suspensions were prepared with RPMI-1640 culture medium containing 10% fetal bovine serum (Gibco, CA, USA). Then, each well in a 96-well plate (Corning Life Sciences (Wujiang) Co., Ltd. Wujiang, China) was inoculated with 100 μL of this medium containing approximately 5 × 10^4^ cells/mL for 24 h of culture at 37 °C with 5% CO_2_. Then, solutions of the test compounds were added in each well. Eight different concentrations of the test compounds were employed. Each treatment consisted of three wells, with three parallel replicates.

After that, to the cell suspensions, 10 μL of CCK-8 stock solution (MedChemExpress, Shanghai, China) was directly added after 48 h of cell cultivation at 37 °C, followed by further cultivation for 1 to 4 h in the dark operation, real-time observation. The OD value of each well was measured and recorded at 450 nm using a microplate reader (Multiskan MK3, Thermo, Suzhou Science Instrument Co., Ltd. Suzhou, Jiangsu, China) to generate cell growth curves. The IC_50_ values of the compounds were calculated using GraphPad Prism 8 software (version 8.0.2, GraphPad Software Inc., Santiago, MN, USA), and the experimental results are expressed as the means ± SD.

#### 3.4.2. Cell Apoptosis Assay

The Annexin V–FITC/PI double-staining was used to detect cell apoptosis (DOJINDO, Kumamoto-ken, Japan). Compound-treated cells were trypsinized, washed twice with PBS, and transferred to microcentrifuge tubes for centrifugation at 1000 rpm for 5 min at room temperature. The cell suspension density was adjusted to 1 × 10^6^/mL with 1× Annexin V binding solution. Then, Annexin V–FITC (5 μL) and PI (5 μL) solutions were added to 100 μL of the cell suspension. The cells were incubated at room temperature for 15 min in the dark, and then 400 μL of 1× Annexin V binding solution was added. Finally, the cells were analyzed by flow cytometry (ACEN, NovoCyte, ACEA Biosciences Inc., Santiago, MN, USA).

#### 3.4.3. Cell-Cycle Assay

After the cells were treated with compound **2j**, they were trypsinized, prepared as a single-cell suspension (1 × 10^6^/mL), and transferred to microcentrifuge tubes for centrifugation at 1500 rpm for 5 min at room temperature; to prevent cell clumping, the cells were fixed by adding ice-cold 70% ethanol (1 mL) and blocking for 15 min at 4 °C. Then, the cells were centrifuged at 1500 rpm for 5 min, and 500 μL of PI solution was added (50 μg/mL PI, 100 μg/mL RNase A, 0.05% Triton X-100) for 40 min of incubation at 37 °C. The cells were centrifuged at 1500 rpm for 5 min, and 1 mL of PBS (HyClone, Logan, UT, USA) was added. After another centrifugation at 1500 rpm for 5 min, the cells were resuspended in 500 μL PBS and analyzed by flow cytometry.

## 4. Conclusions

In summary, 33 derivatives of chelerythrine and sanguinarine were designed and synthesized by using suitable nucleophilic substances for addition reactions, and their antileukemia activities against the Jurkat Clone E6-1 and THP-1 cell lines were evaluated for the first time. By analyzing these derivatives, some initial SARs were revealed. For example, the presence of cyano and malonic esters groups at the C-6 position of the benzophenanthridine skeleton resulted in stronger antileukemia activity, whereas the introduction of hydroxyethyl, acetonyl, or other groups at this position led to decreased activity. Moreover, compounds containing methylenedioxy moieties at the C-7 and C-8 positions had better antileukemia activity. Thus, when methylenedioxy groups were at the C-7 and C-8-positions, the introduction of cyano or malonic esters groups at the C-6 position could result in the best antileukemia activity.

Further studies indicated that compound **2j** induced apoptosis in both Jurkat Clone E6-1 and THP-1 cells in a dose-dependent manner, and these results were consistent with those from the CCK-8 assay. The inhibitory effects of compound **2j** might be related to cell-cycle changes, and these data were consistent with the apoptosis detection results. These findings became clearer after treatment with 1.0 μM **2j** for 48 h. In conclusion, compound **2j** induced apoptosis in Jurkat Clone E6-1 and THP-1 cells and arrested these cells in the G_0_/G_1_ phase, possibly by disrupting the cell-cycle, reducing DNA synthesis, and inducing apoptosis. These mechanisms led to inhibition of the proliferation and growth of leukemia cells.

Among all of the prepared compounds, compound **2j** showed satisfactory activity against Jurkat Clone E6-1 and THP-1 cells, and it could be considered for further investigation and optimization. However, since we selected transformed leukemia cells, this might have some drawbacks. In order to explore whether the cytotoxicity observed is specific to the leukemia lines, we will continue to test the compounds on a non-transformed cell type in future experiments. Lastly, our research suggested that compound **2j** might be a potentially useful starting point for further optimization to become a new lead compound, providing a rich and diverse material basis for the development of innovative antileukemia drugs.

## Data Availability

The data presented in this study are available in the Supplementary Materials.

## Supplementary Materials

The following supporting information can be downloaded at: https://www.mdpi.com/article/10.3390/molecules27123934/s1, Figures S1–S138: ^1^H-NMR, ^13^C-NMR, and HR-ESI-MS spectra for compounds **1a**–**1u** and **2a**–**2l;** Table S1: The mobile phase system, injection volume, and detection wavelength of compounds; Table S2: Preliminary screening test results.
