# Supplementary material for: Synthesis and Antileukemia Activity Evaluation of Benzophenanthridine Alkaloid Derivatives
Source: Molecules. 2022 Jun 19;27(12):3934. doi: 10.3390/molecules27123934 (PMC9227418; doi:10.3390/molecules27123934)
Supplement: Supplementary file 1 [file molecules-27-03934-s001.zip › molecules-1712948-supplementary.pdf]

molecules

Supplementary Material

Synthesis and Antileukemia Activity Evaluation of Benzophenanthridine Alkaloid Derivatives

Yaling Tang [1], Xinglian Xu [2,3], Jiang Li [2,3], Lulu Deng [2,3,*] and Shuzhen Mu [2,3,*]

1 College of Pharmacy, Guizhou University, Guiyang 550025, China; gs.yltang@gzu.edu.cn
2 State Key Laboratory of Functions and Applications of Medicinal Plants, Guizhou Medical University, Guiyang 550014, China; xinglian_xu2022@163.com (X.X.); jiangli@gzcnp.cn (J.L.)
3 The Key Laboratory of Chemistry for Natural Products of Guizhou Province, Chinese Academy of Sciences, Guiyang 550014, China
* Correspondence: luludeng@gzcnp.cn (L.D.); muzi0558@126.com (S.M.)

1. **Table of contents...Page No.**

2. ^{1}H-NMR and ^{13}C-NMR spectra for compounds **1** and **2** 2
3. ^{1}H-NMR, ^{13}C-NMR, and HR-ESI-MS spectra for compounds **1a**–**1u** and **2a**–**2l**.. 4
4. The HPLC chromatograms of compounds **1**, **2**, **1a**–**1u,** and **2a**–**2l** 54
5. The preliminary screening results... 66

^{1}H-NMR and ^{13}C-NMR spectra for compounds 1 and 2

Compound **1**

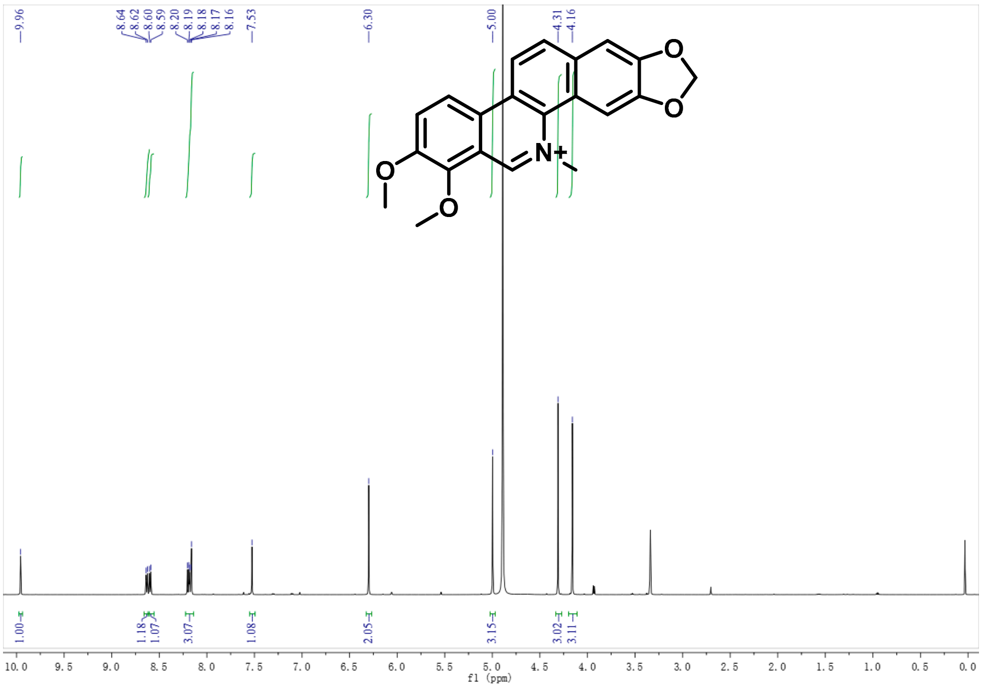

Figure S1. ^{1}H-NMR spectrum of **1** (600 MHz, CD_3OD).

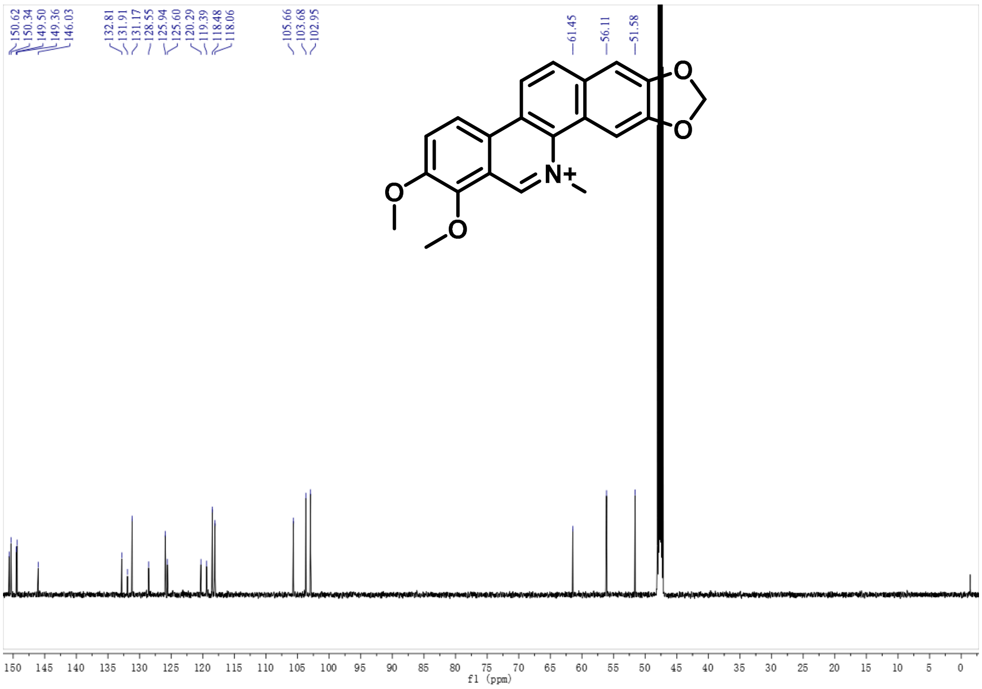

Figure S2. ^{13}C-NMR spectrum of **1** (150 MHz, CD_3OD).

Compound **2**

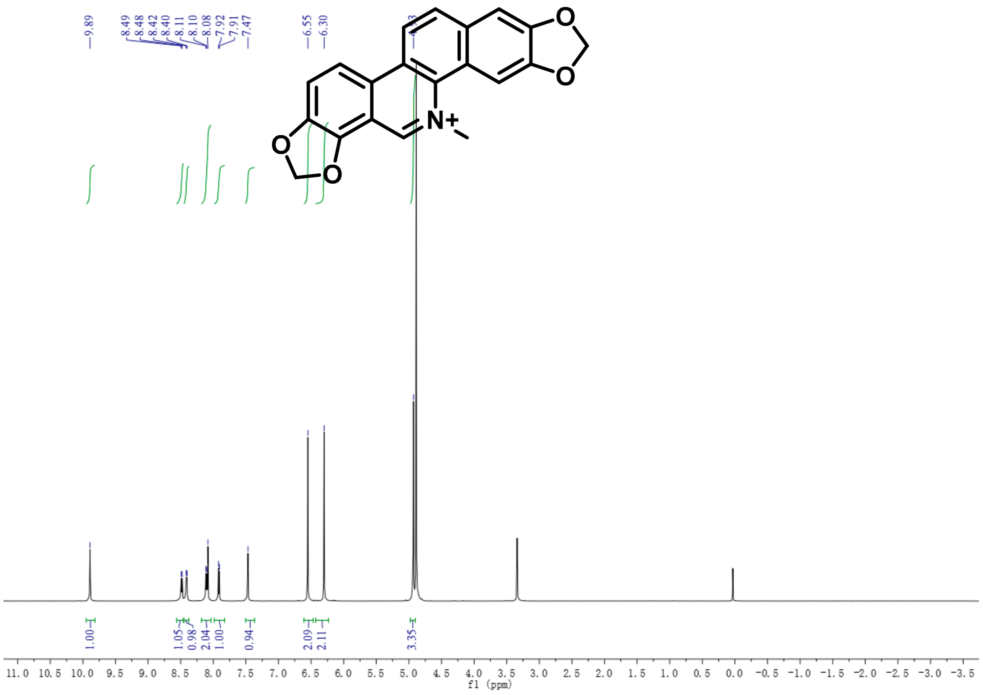

Figure S3. ^{1}H-NMR spectrum of **2** (600 MHz, CD_3OD).

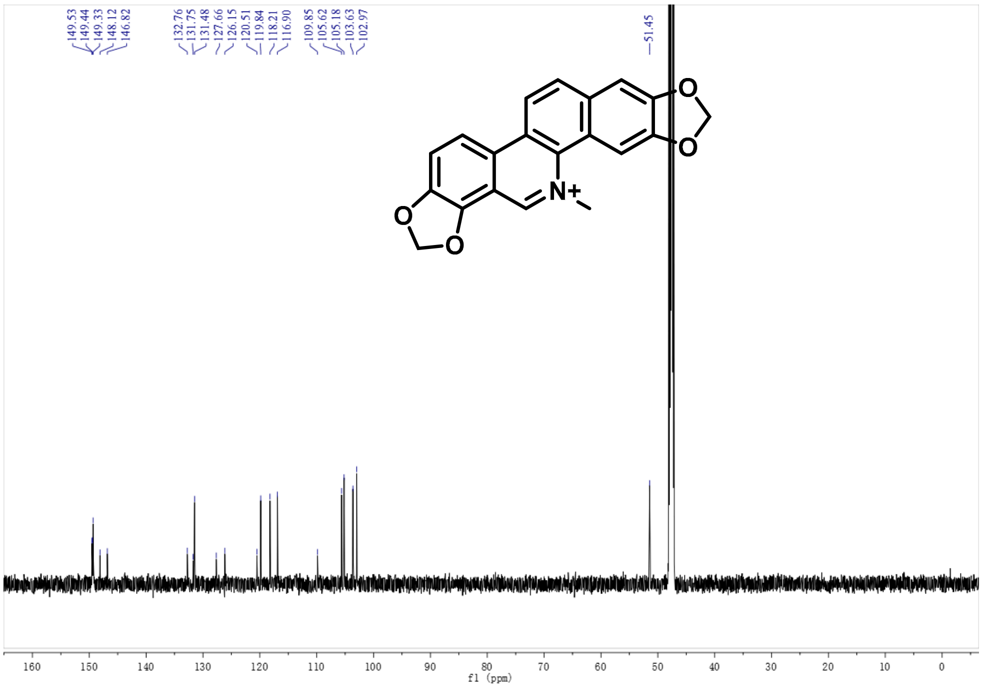

Figure S4. ^{13}C-NMR spectrum of **2** (150 MHz, CD_3OD).

^{1}H-NMR, ^{13}C-NMR, and HR-ESI-MS spectra for compounds 1a–1u and 2a–2l

Compound **1a**

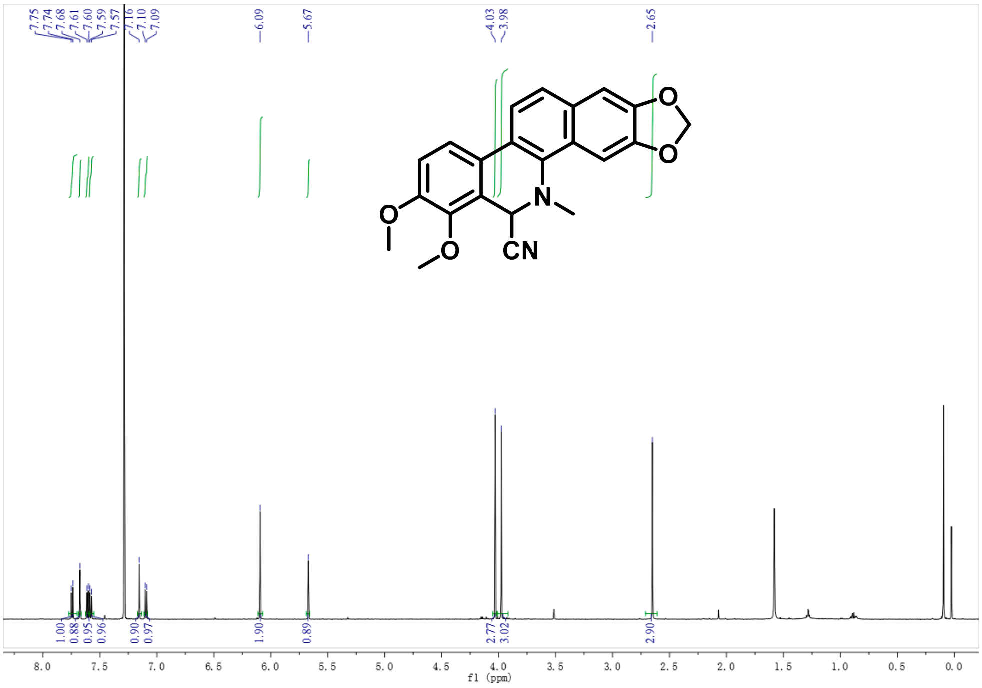

Figure S5. ^{1}H-NMR spectrum of **1a** (600 MHz, $CDCl_3$).

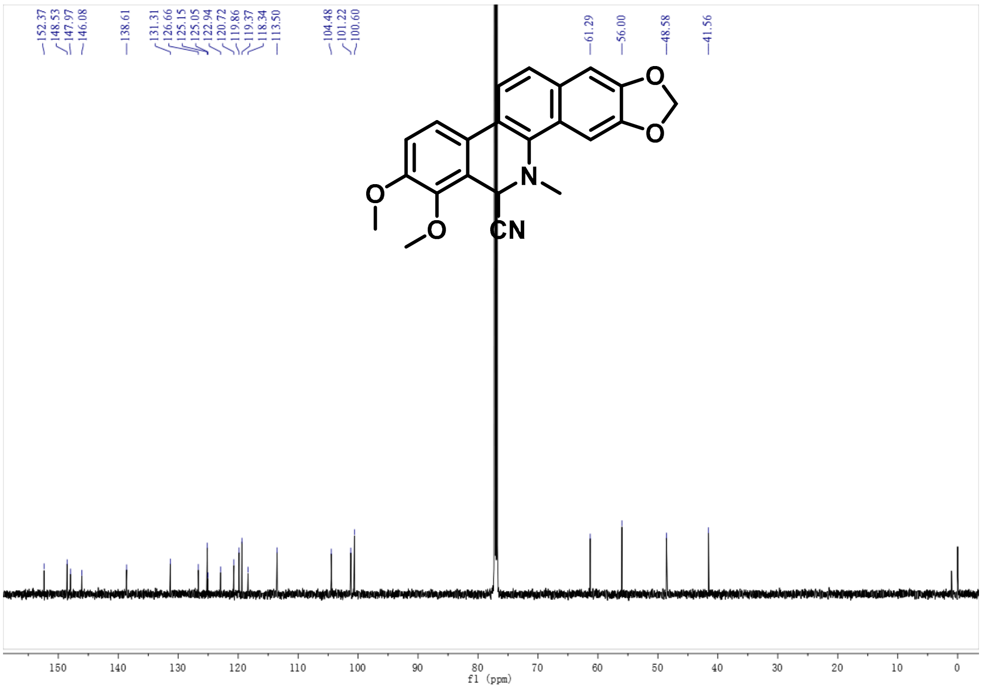

Figure S6. ^{13}C-NMR spectrum of **1a** (150 MHz, $CDCl_3$).

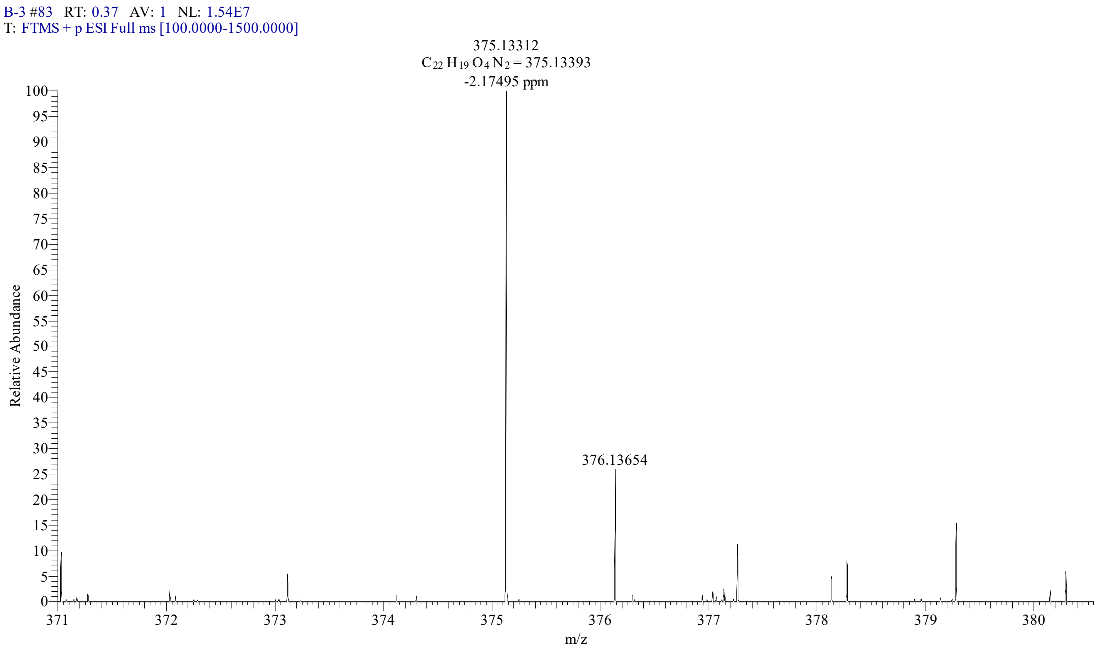

Figure S7. HR-ESI-MS spectrum of **1a.**

Compound **1b**

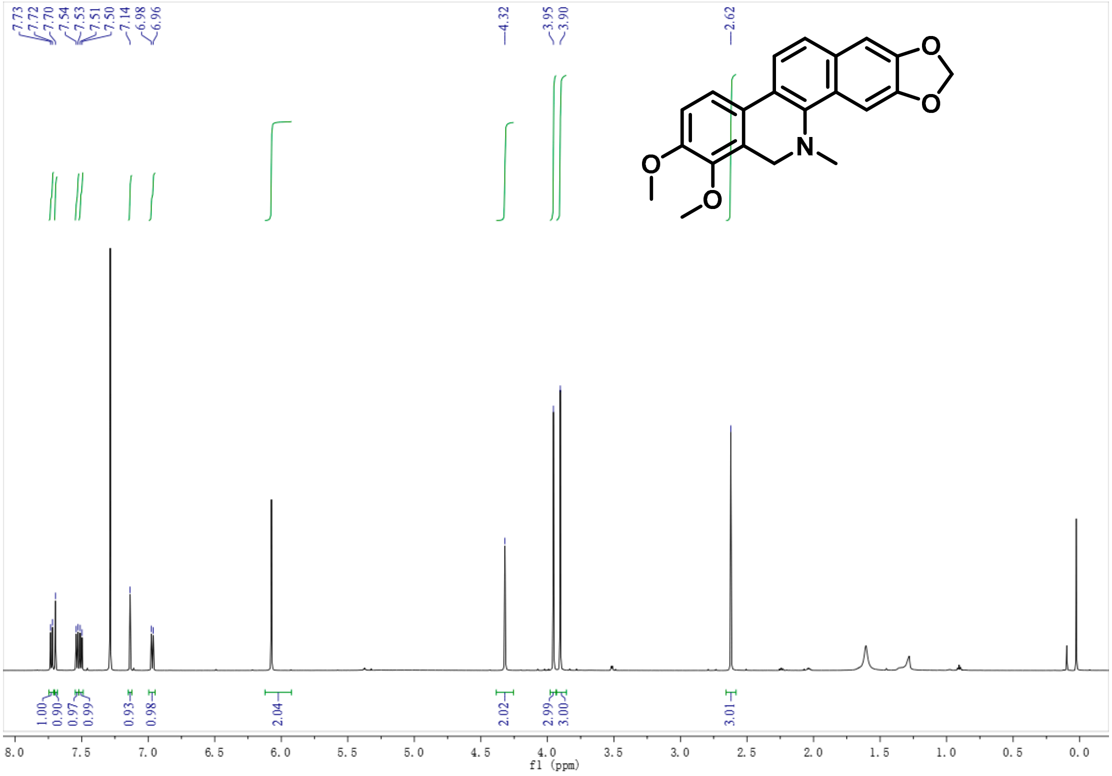

Figure S8. ^{1}H-NMR spectrum of **1b** (600 MHz, $CDCl_3$).

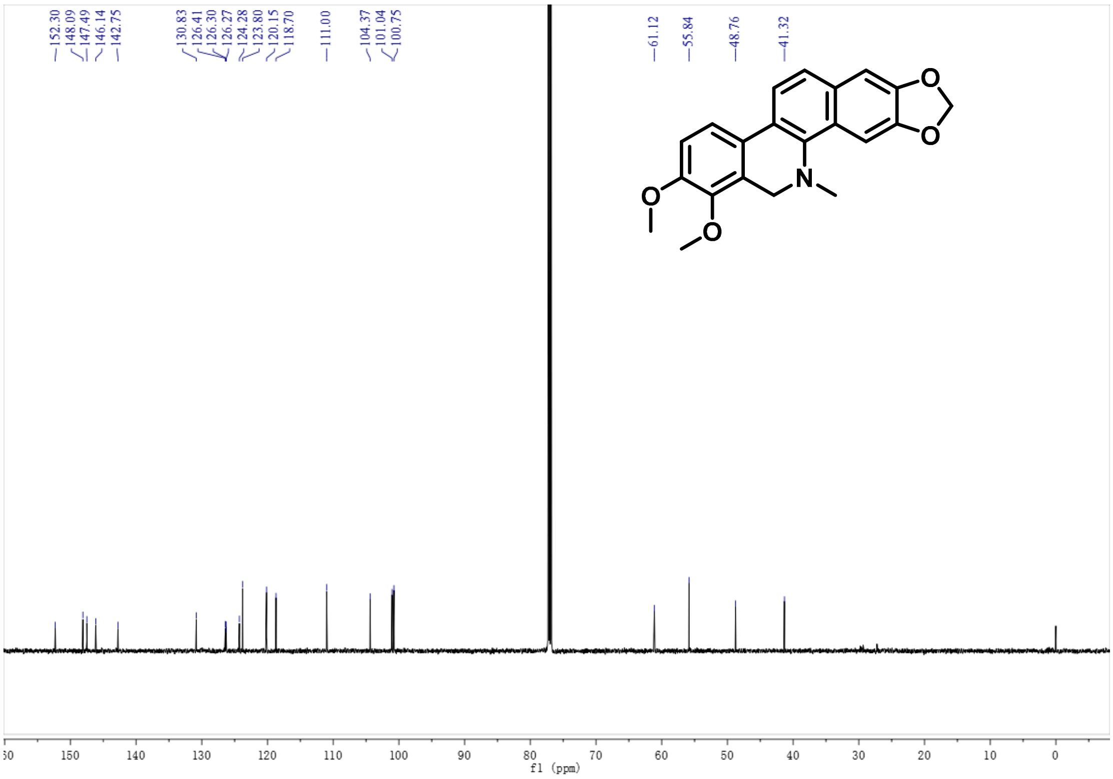

Figure S9. ^{13}C-NMR spectrum of **1b** (150 MHz, $CDCl_3$).

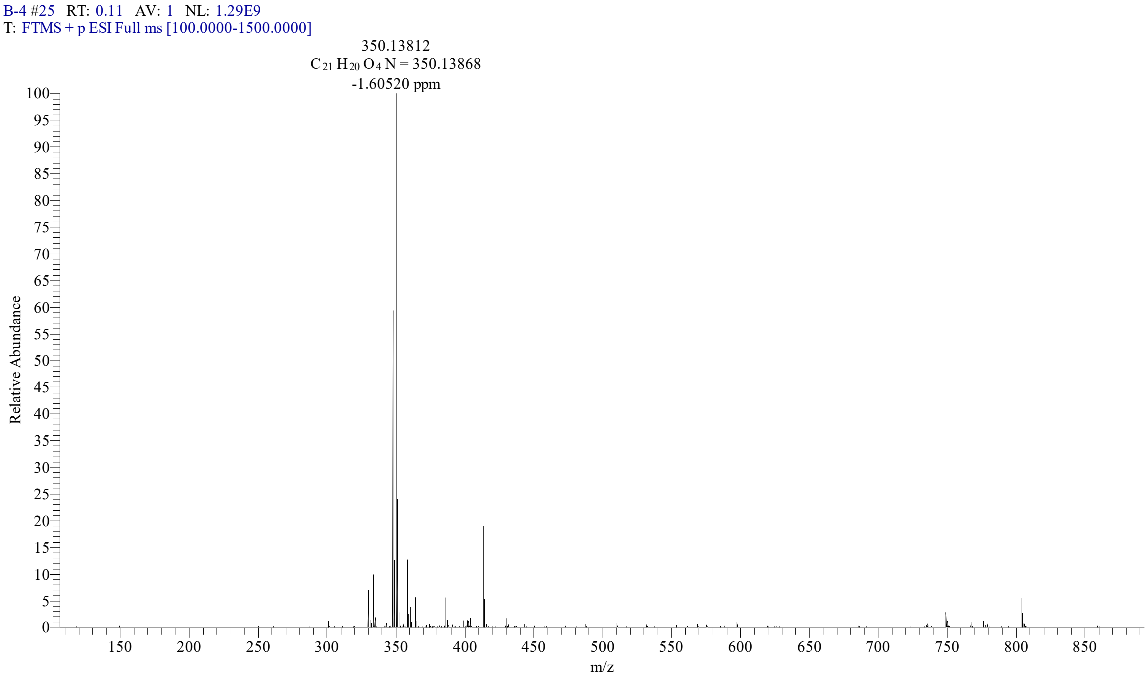

Figure S10. HR-ESI-MS spectrum of **1b.**

Compound **1c**

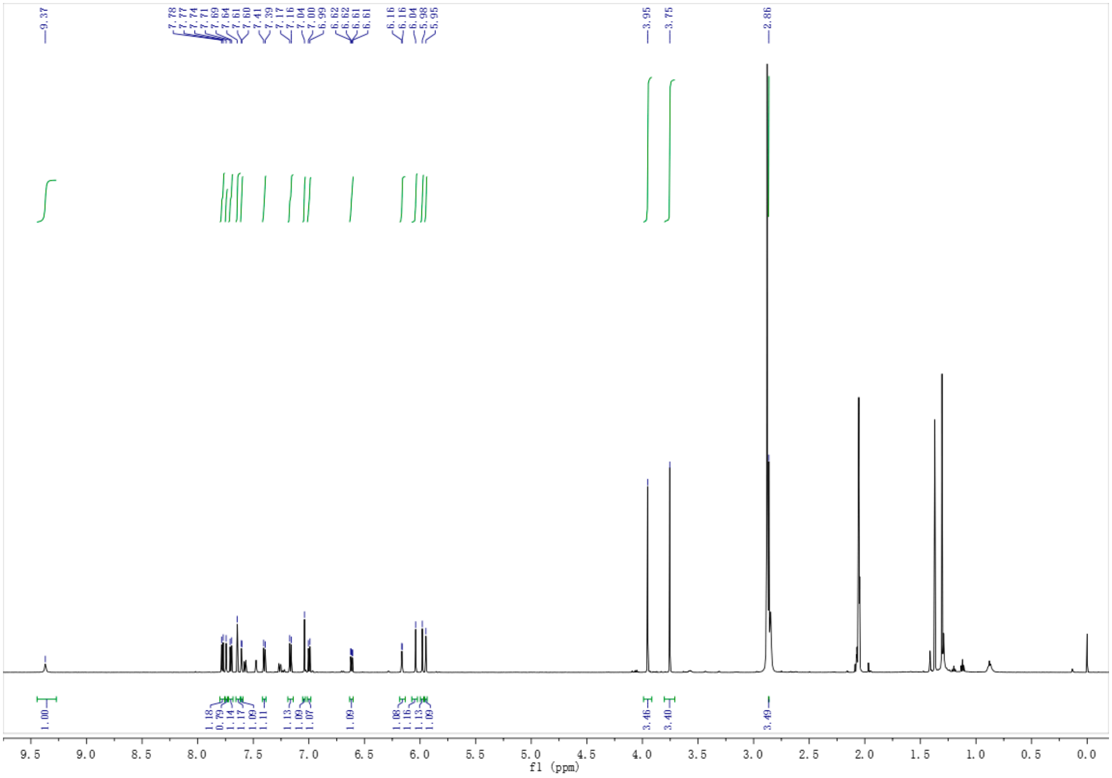

Figure S11. ^{1}H-NMR spectrum of **1c** (600 MHz, acetone-d_6).

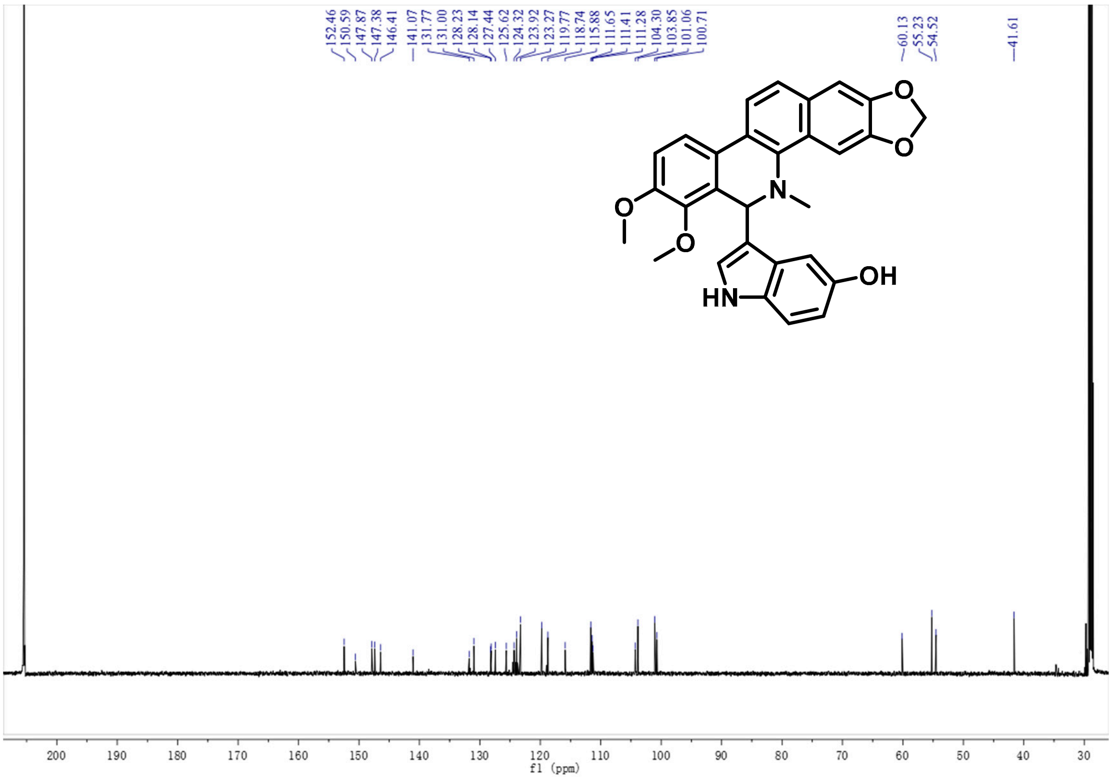

Figure S12. ^{13}C-NMR spectrum of **1c** (150 MHz, acetone-d_6).

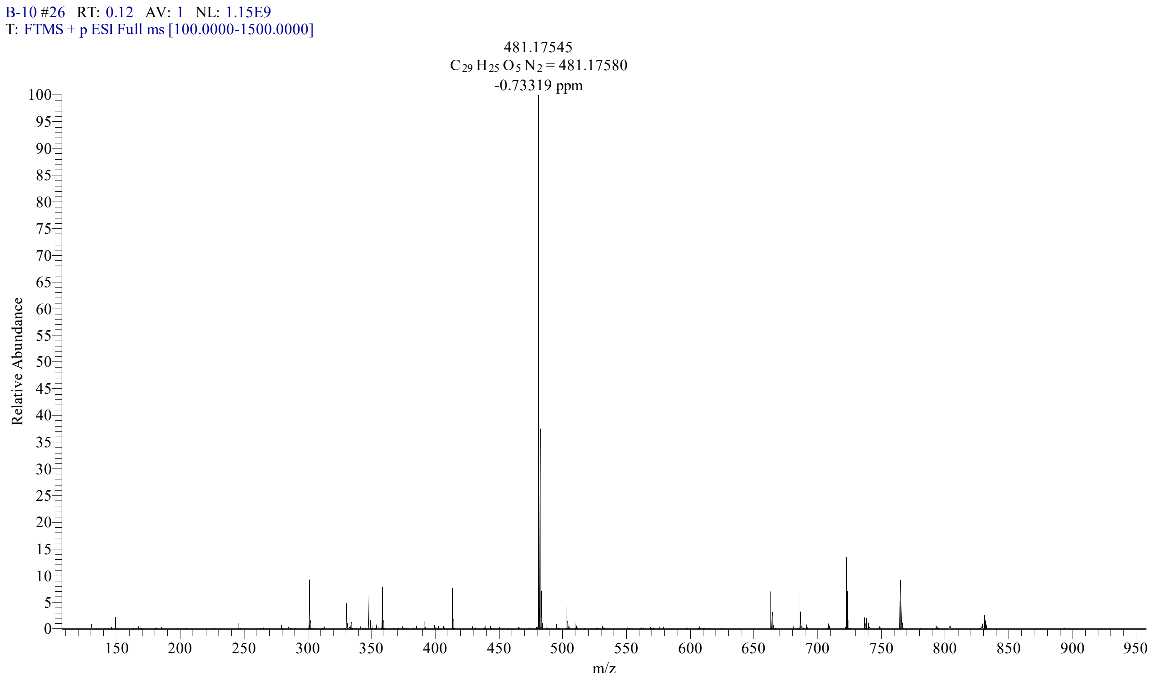

Figure S13. HR-ESI-MS spectrum of **1c.**

Compound **1d**

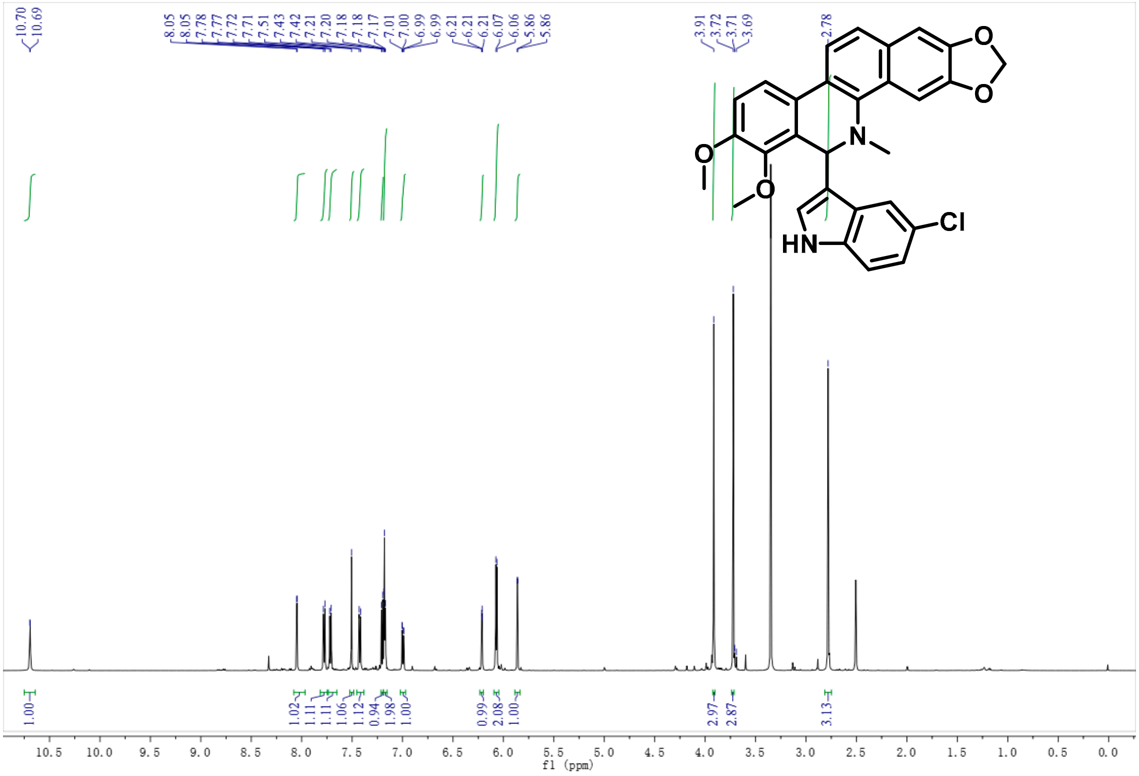

Figure S14. ^{1}H-NMR spectrum of **1d** (600 MHz, DMSO-d_6).

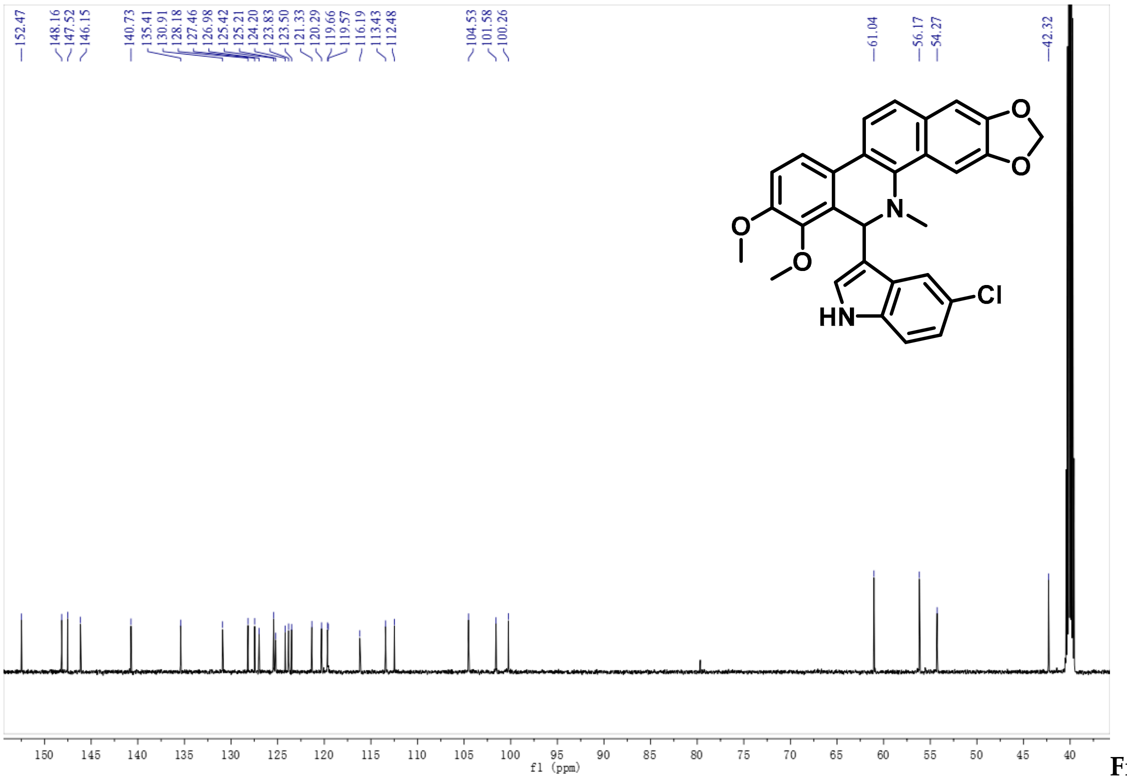

Figure S15. ¹³C-NMR spectrum of **1d** (150 MHz, DMSO-d_6).

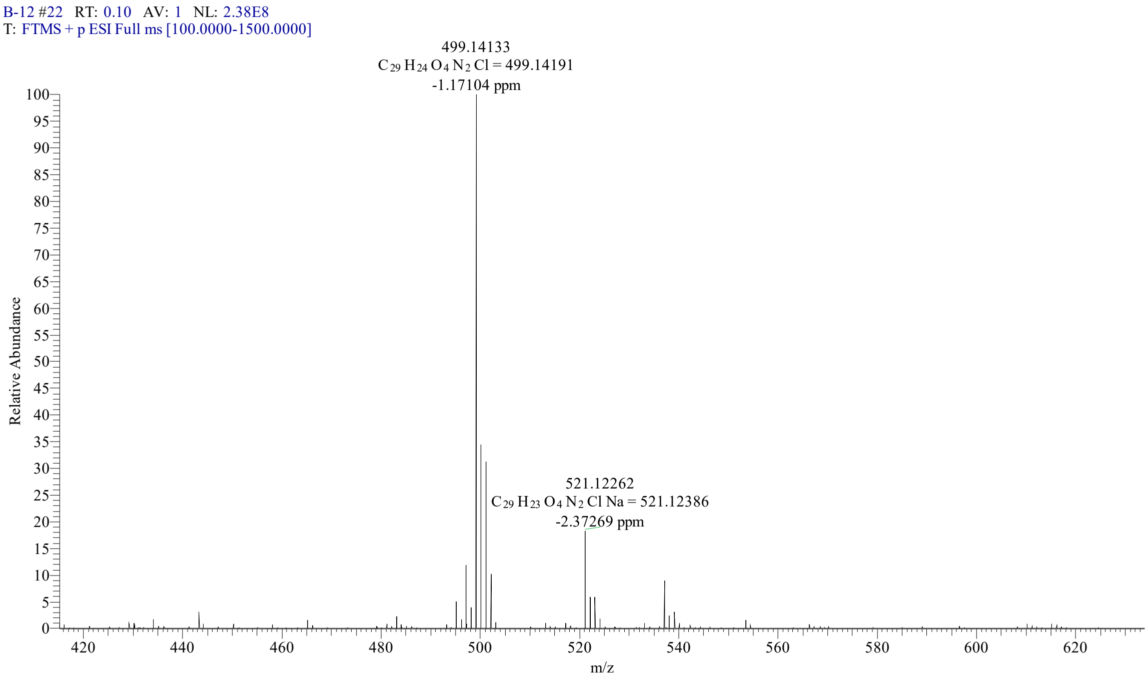

Figure S16. HR-ESI-MS spectrum of **1d.**

Compound **1e**

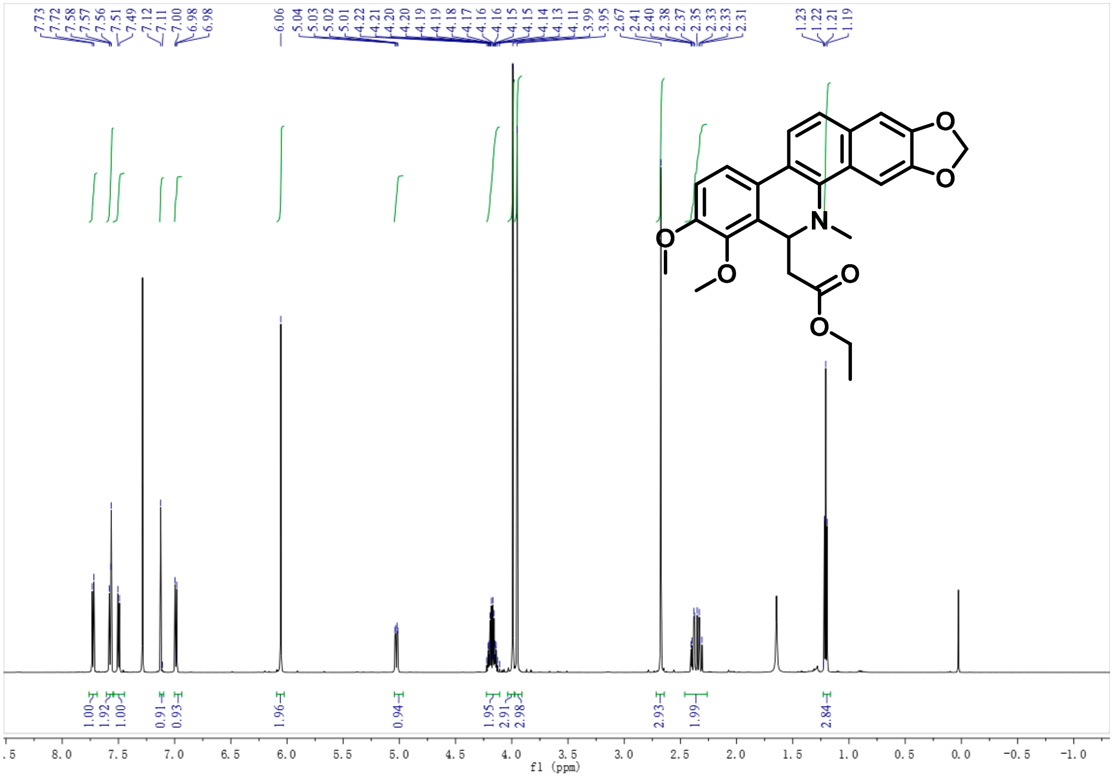

Figure S17. ^{1}H-NMR spectrum of **1e** (600 MHz,$CDCl_3$).

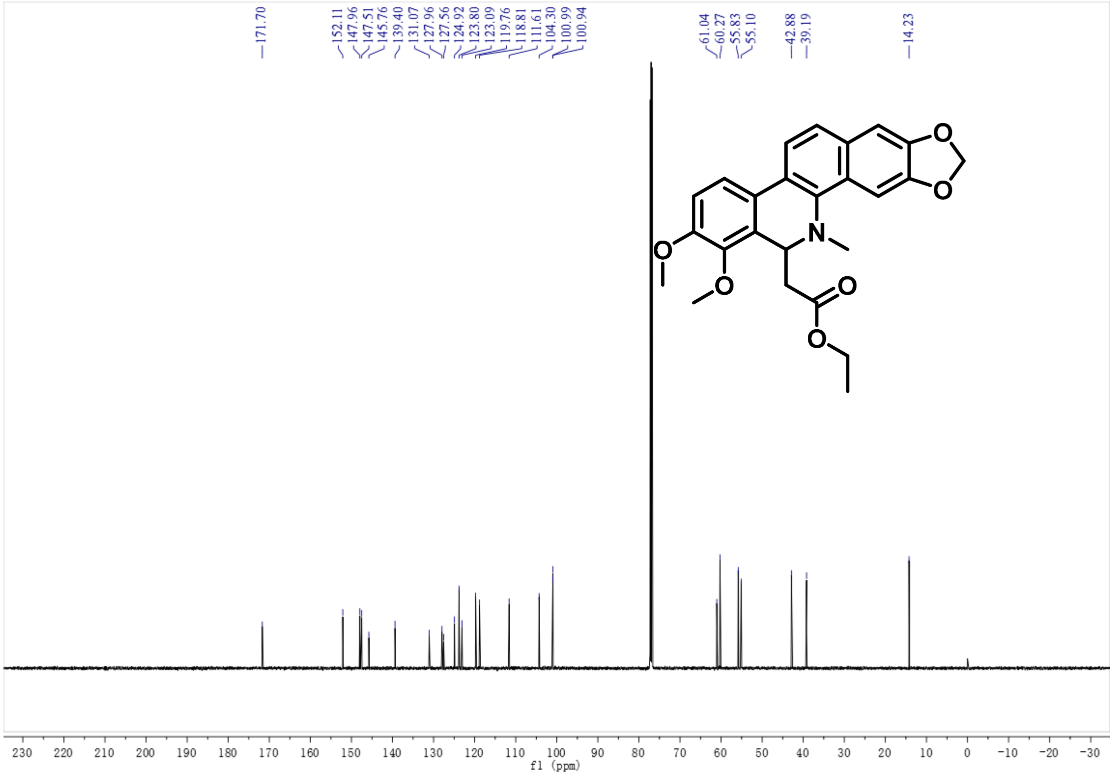

Figure S18. ^{13}C-NMR spectrum of **1e** (150 MHz, $CDCl_3$).

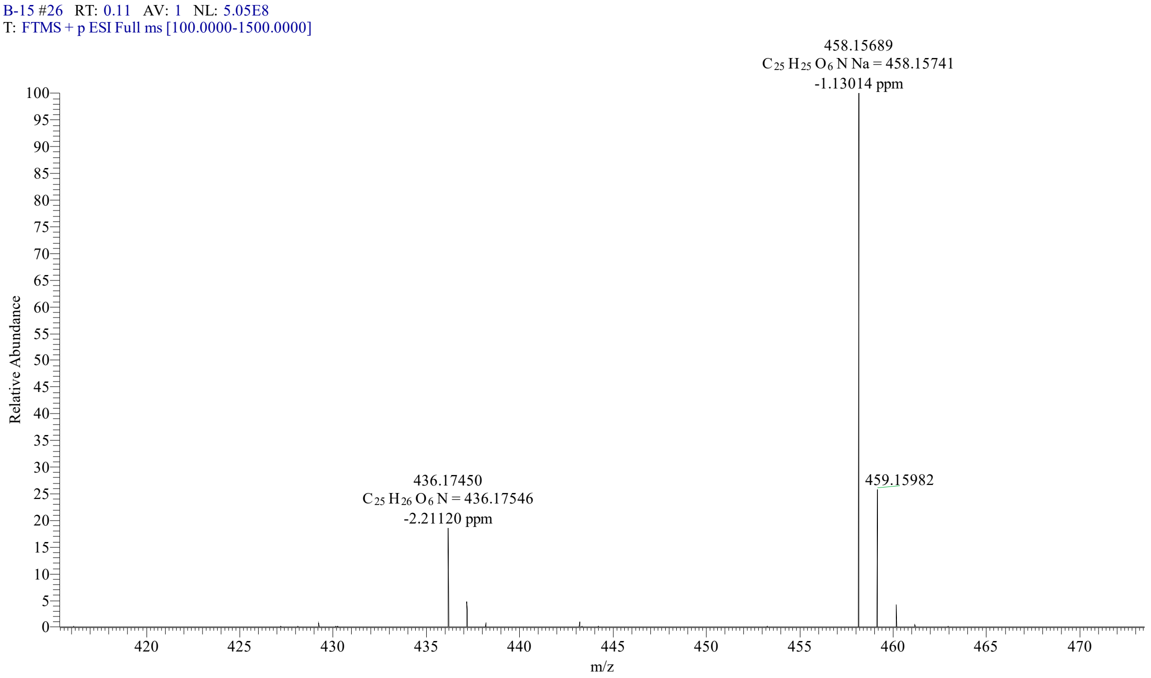

Figure S19. HR-ESI-MS spectrum of **1e.**

Compound **1f**

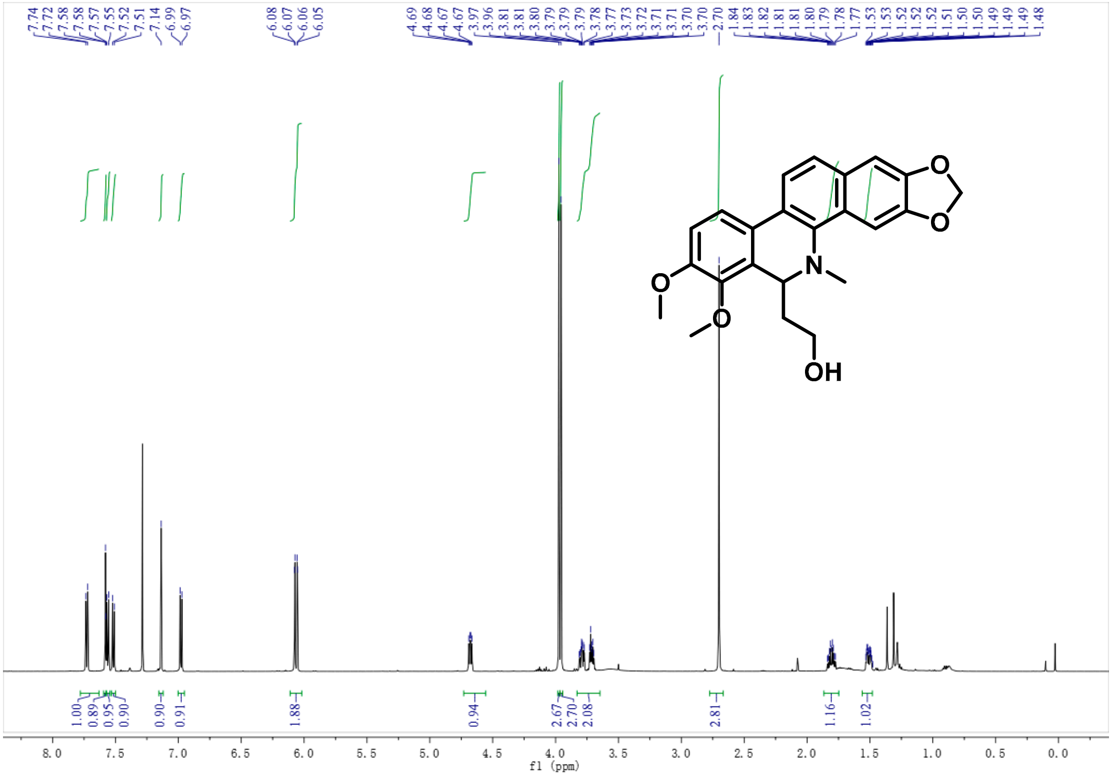

Figure S20. ^{1}H-NMR spectrum of **1f** (600 MHz, $CDCl_3$).

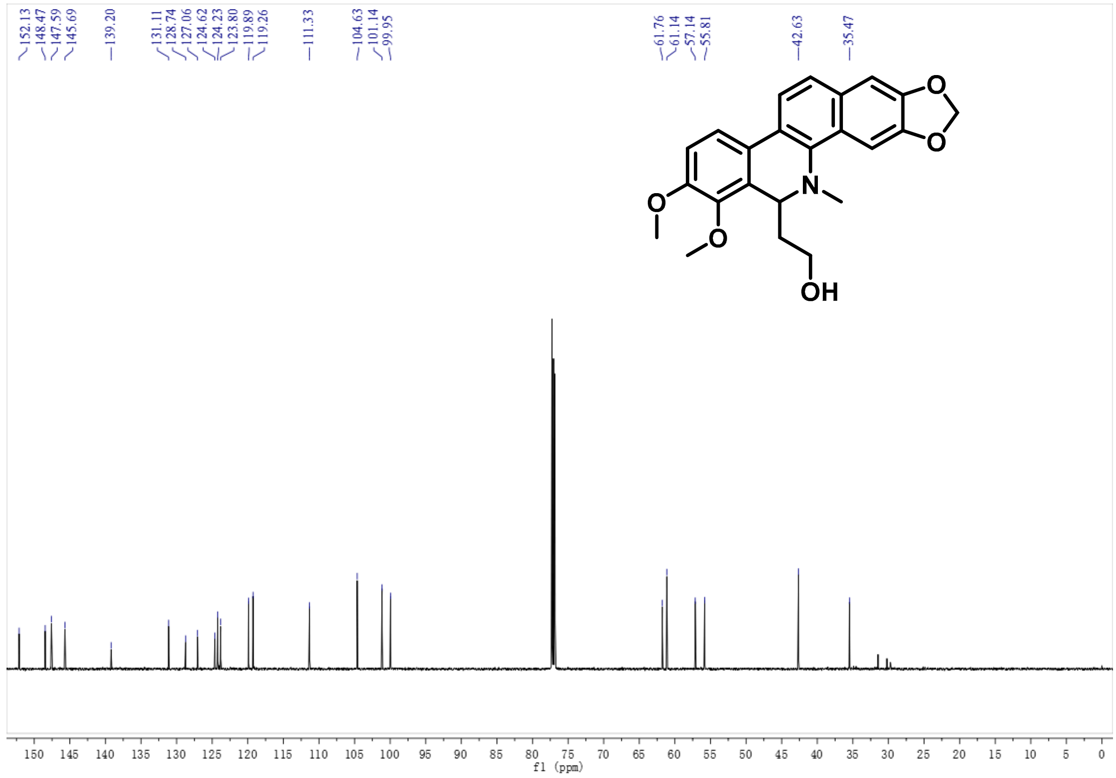

Figure S21. ^{13}C-NMR spectrum of **1f** (150 MHz, $CDCl_3$).

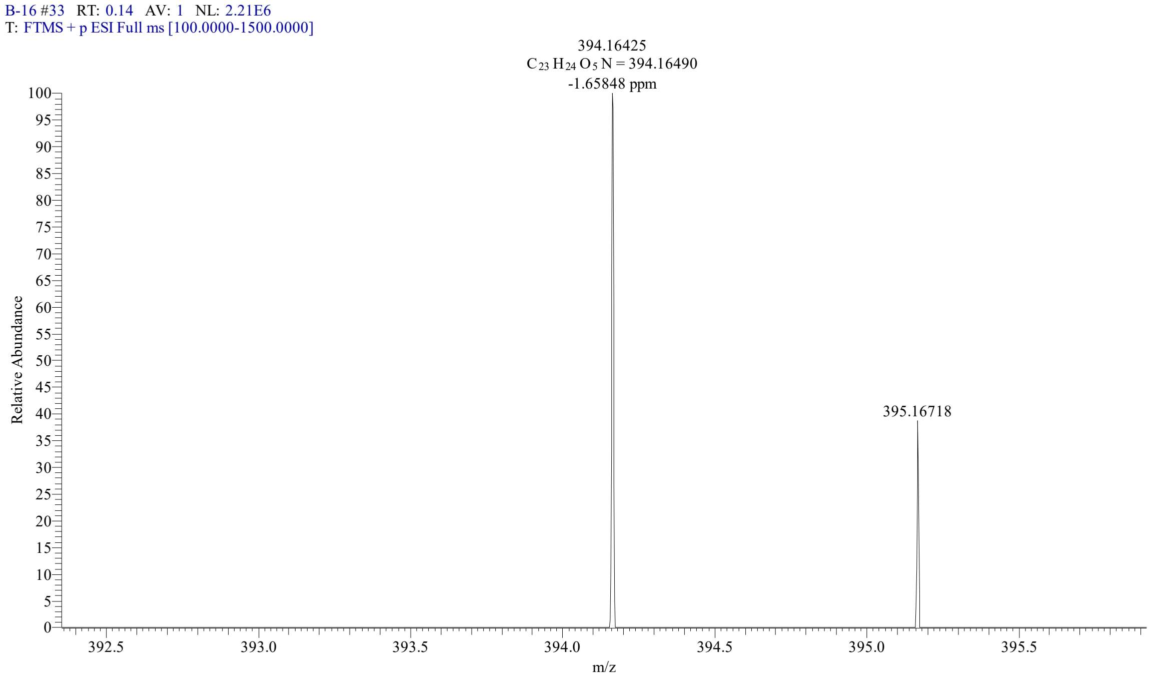

Figure S22. HR-ESI-MS spectrum of **1f.**

Compound **1g**

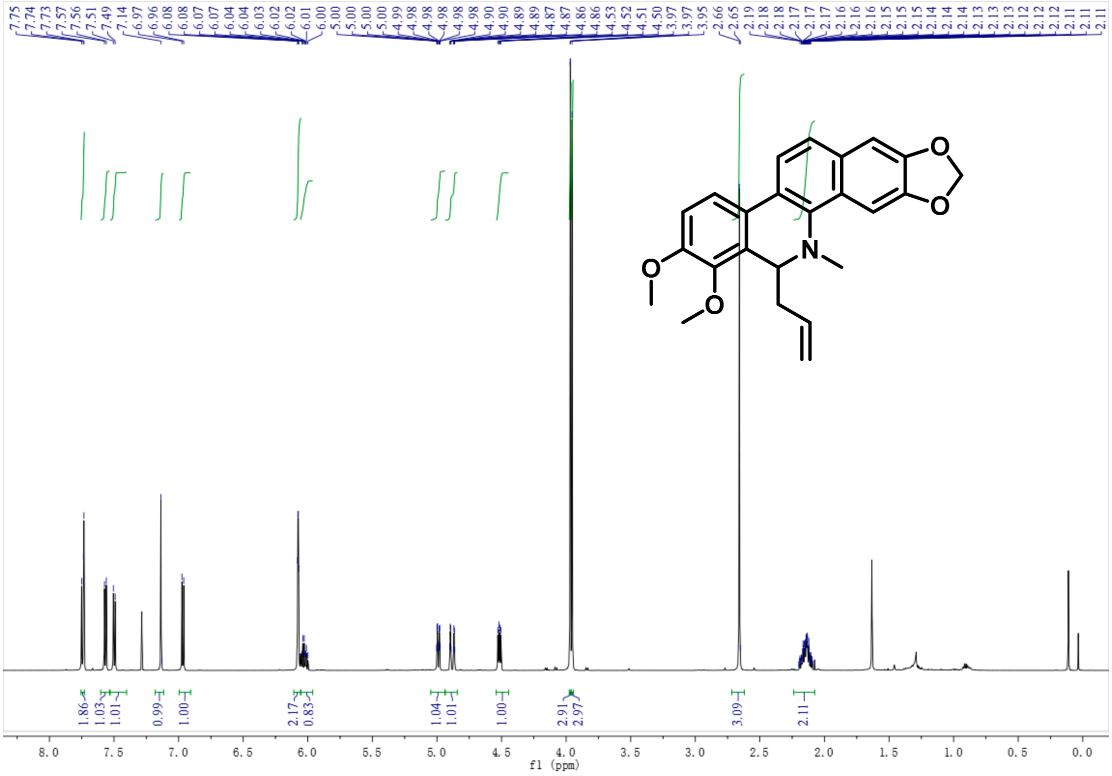

Figure S23. ^{1}H-NMR spectrum of **1g** (600 MHz, $CDCl_3$).

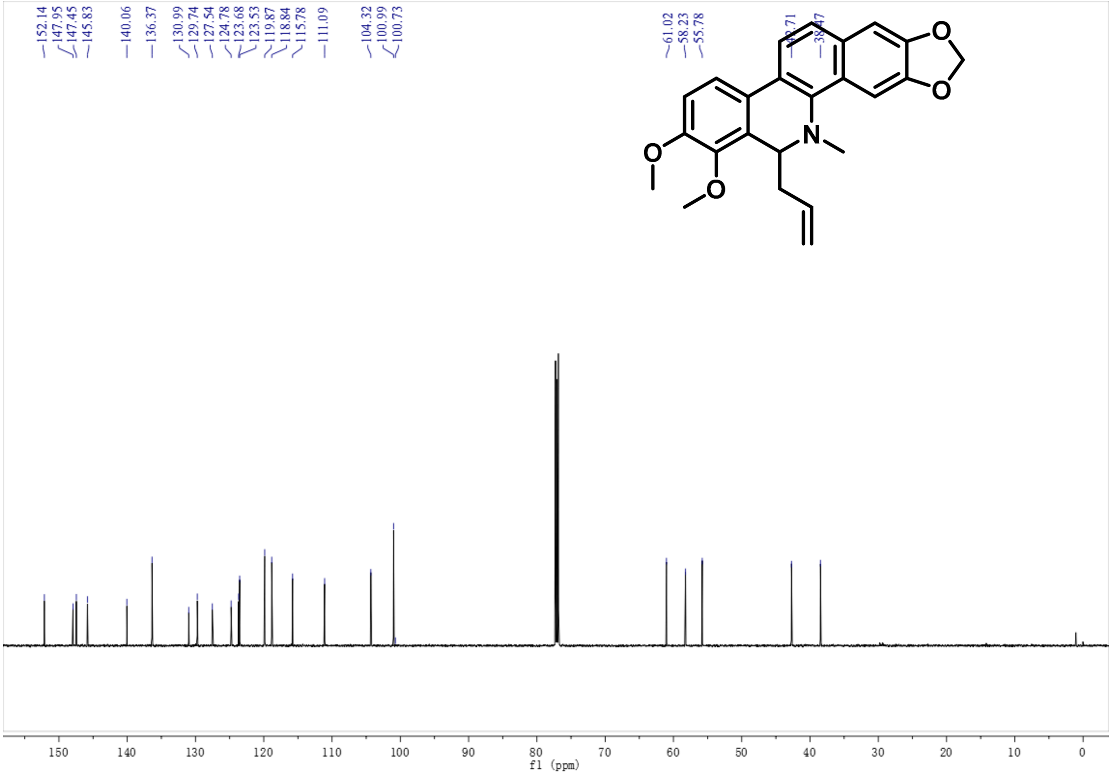

Figure S24. ^{13}C-NMR spectrum of **1g** (150 MHz, $CDCl_3$).

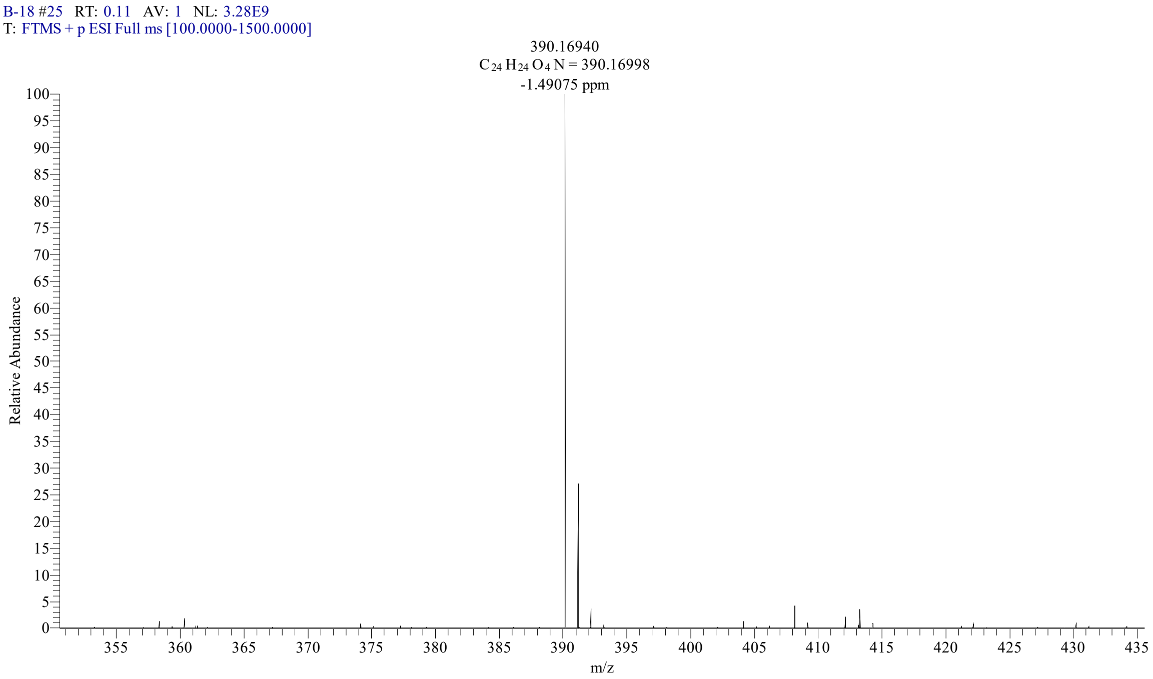

Figure S25. HR-ESI-MS spectrum of **1g.**

Compound **1h**

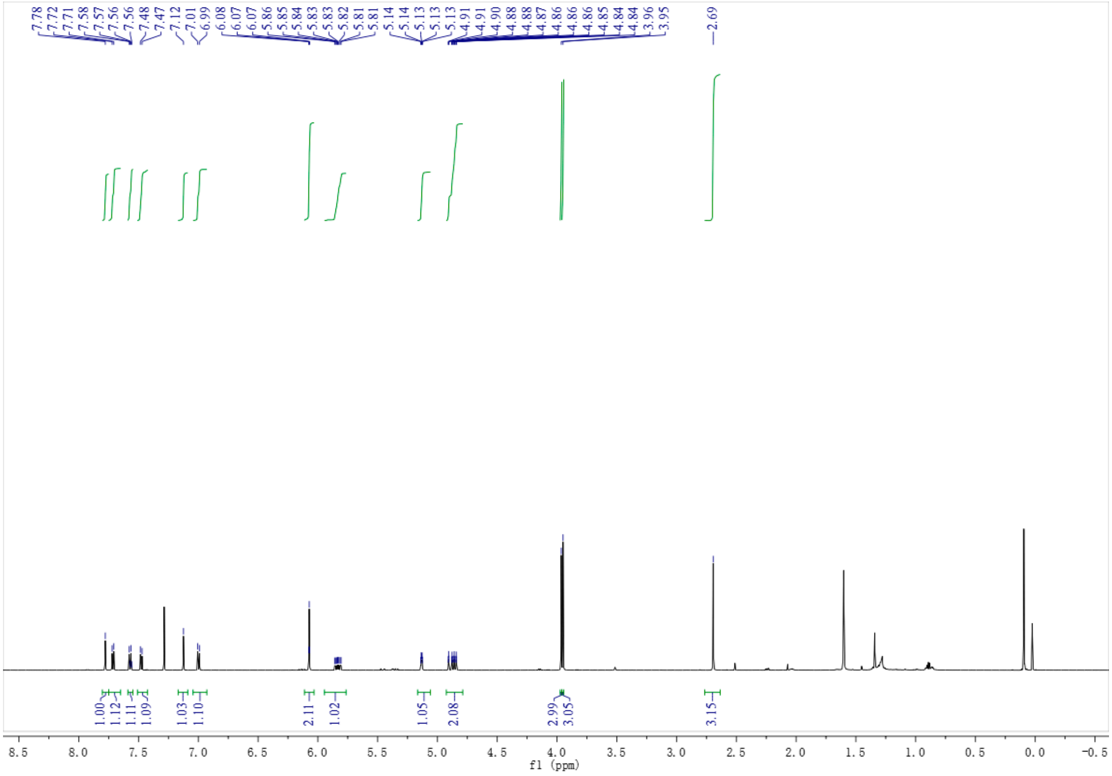

Figure S26. ^{1}H-NMR spectrum of **1h** (600 MHz, $CDCl_3$).

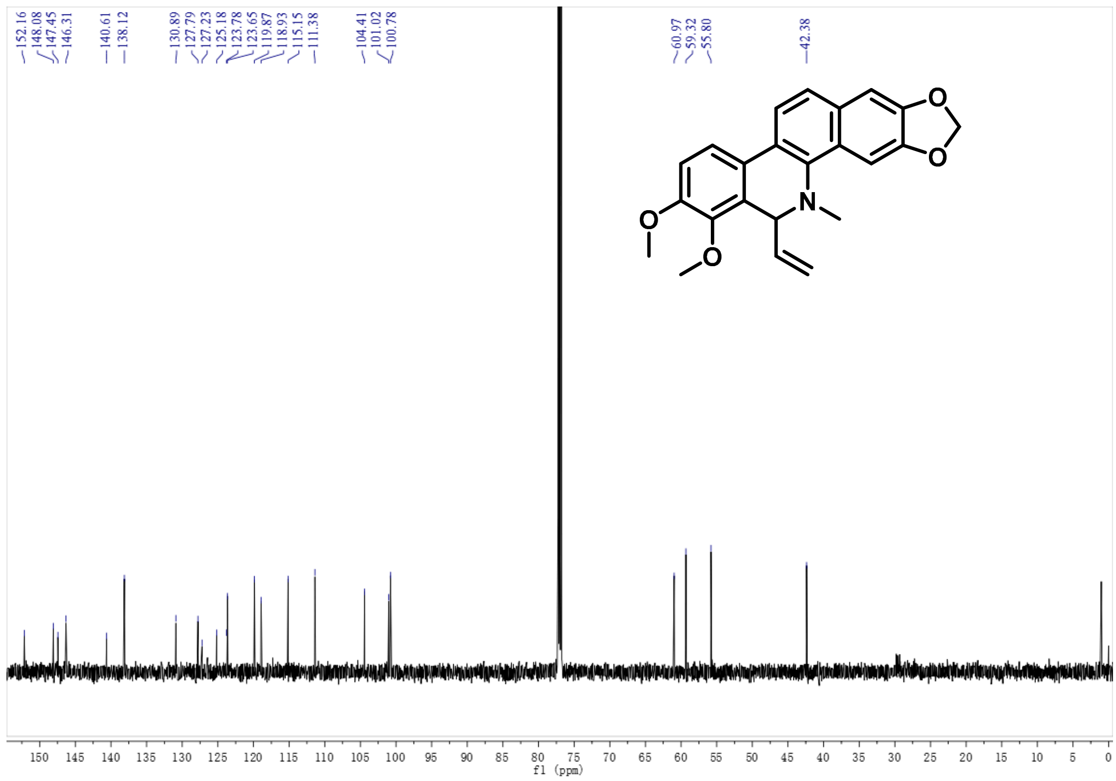

Figure S27. ^{13}C-NMR spectrum of **1h** (150 MHz, $CDCl_3$).

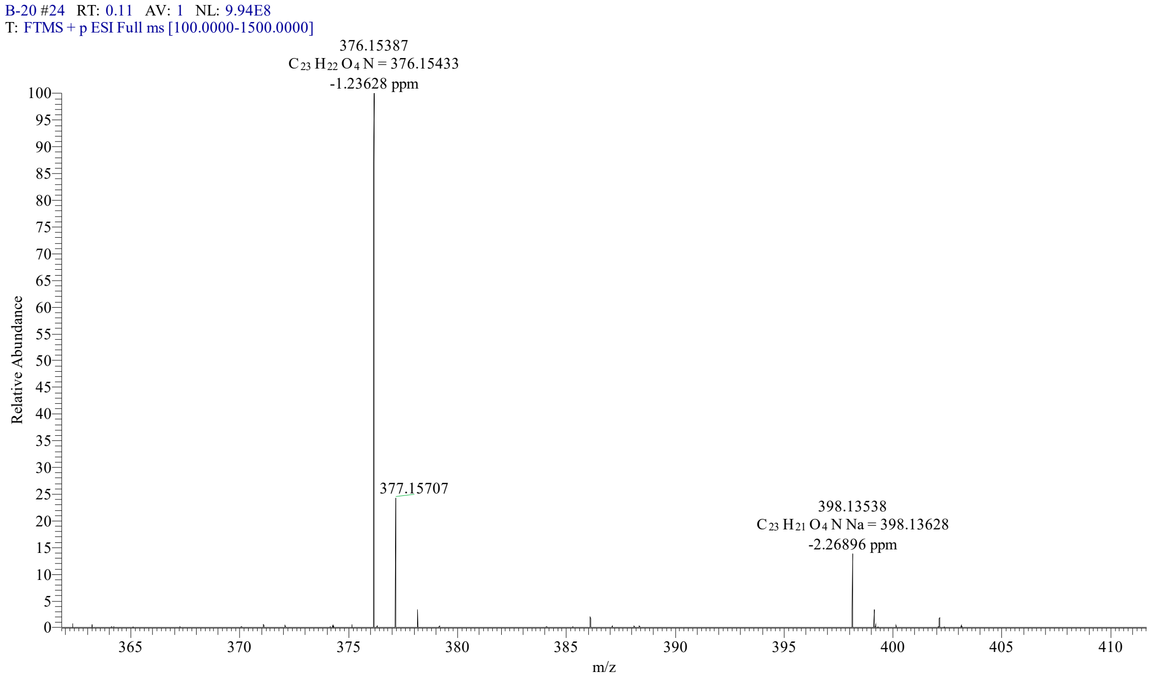

Figure S28. HR-ESI-MS spectrum of **1h.**

Compound **1i**

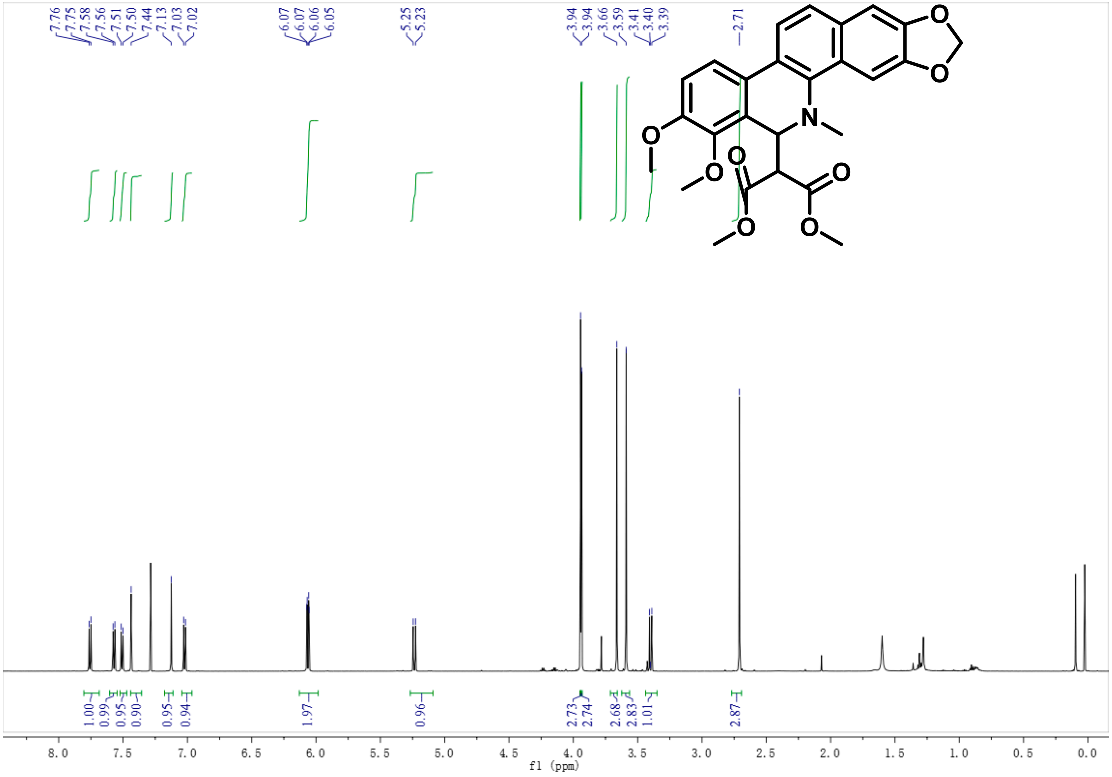

Figure S29. ^{1}H-NMR spectrum of **1i** (600 MHz, $CDCl_3$).

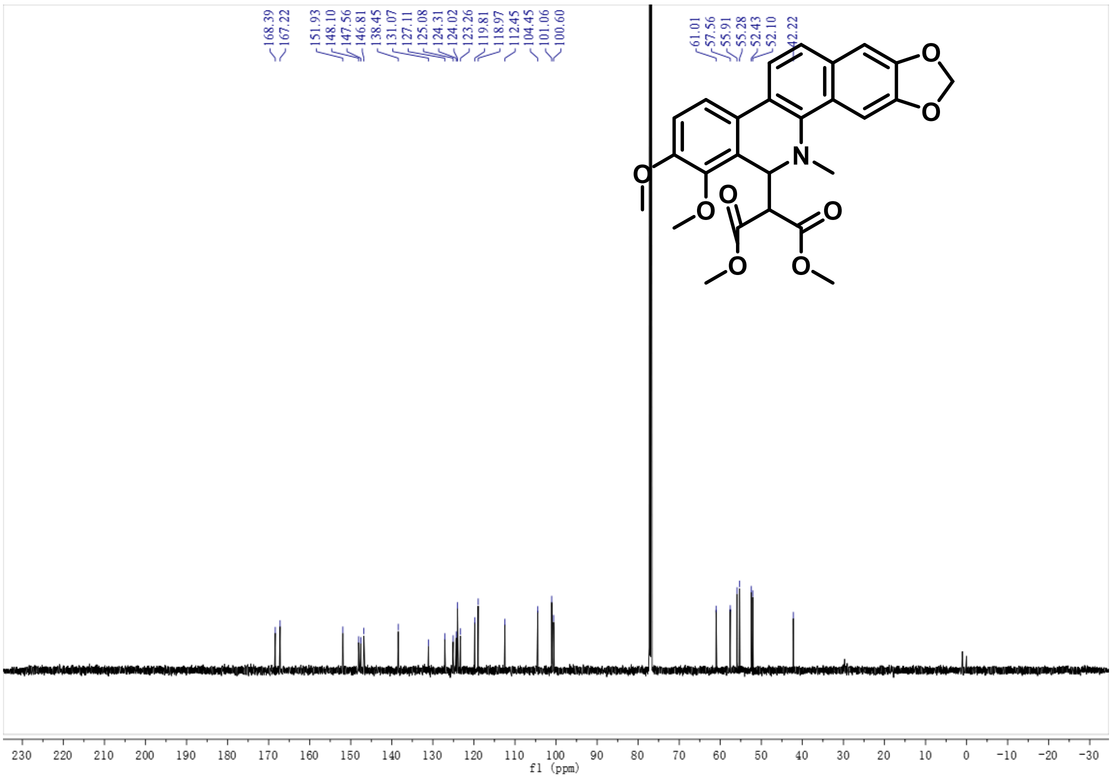

Figure S30. ^{13}C-NMR spectrum of **1i** (150 MHz, $CDCl_3$).

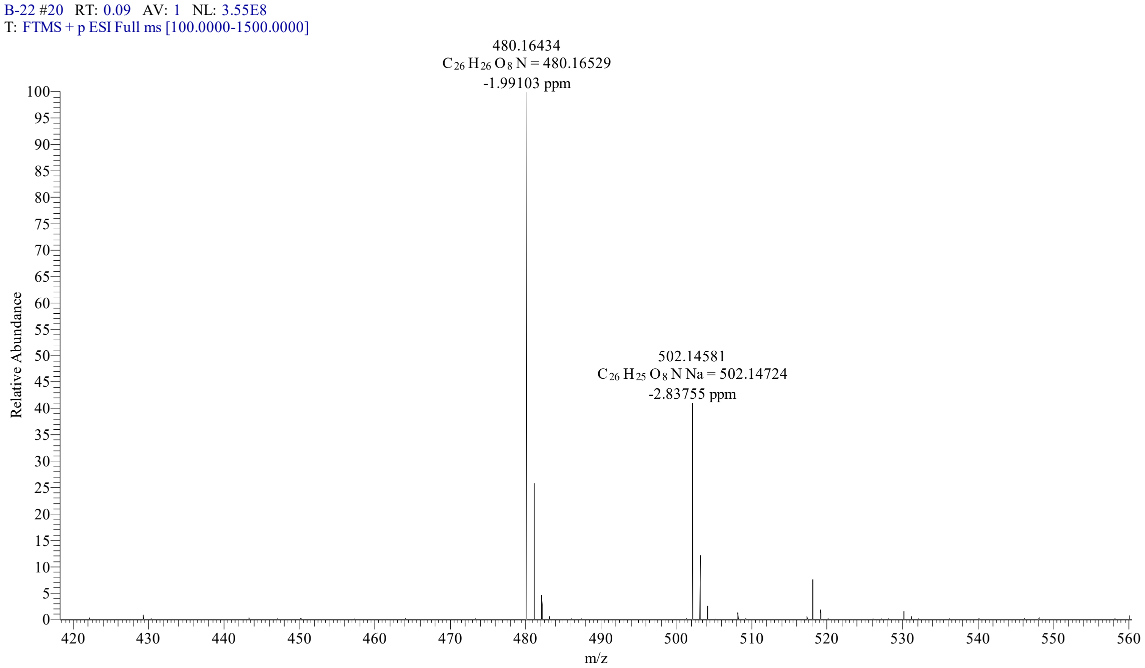

Figure S31. HR-ESI-MS spectrum of **1i.**

Compound **1j**

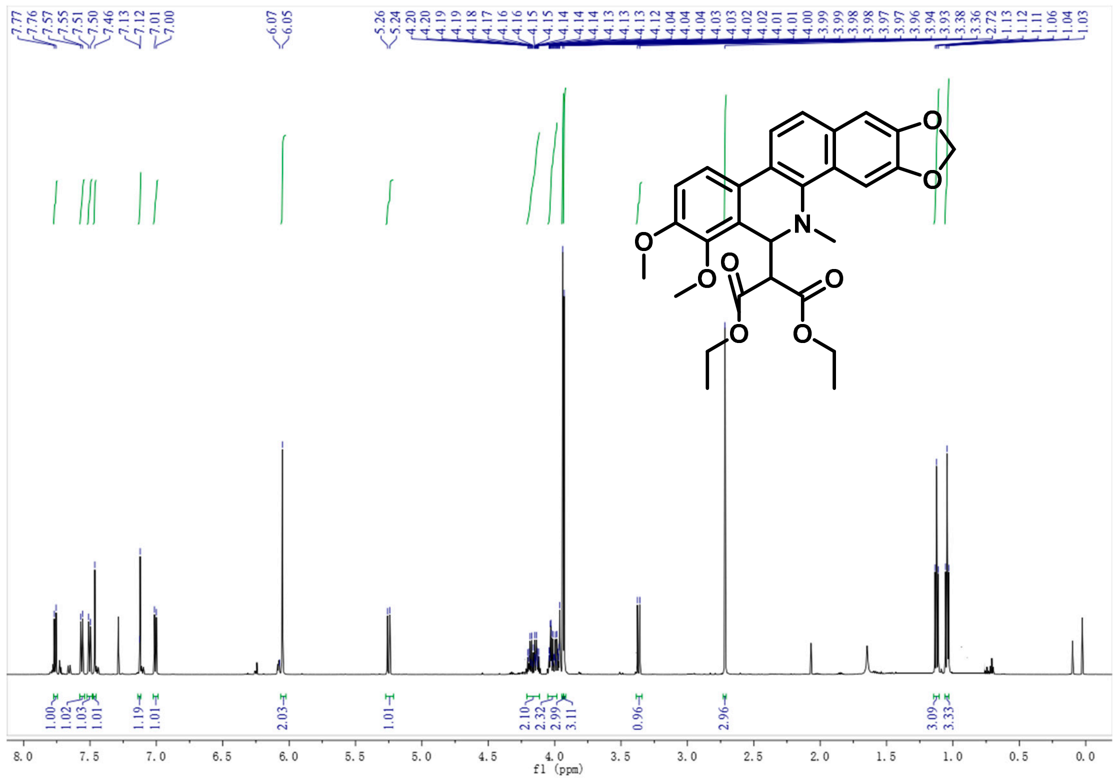

Figure S32. ^{1}H-NMR spectrum of **1j** (600 MHz, $CDCl_3$).

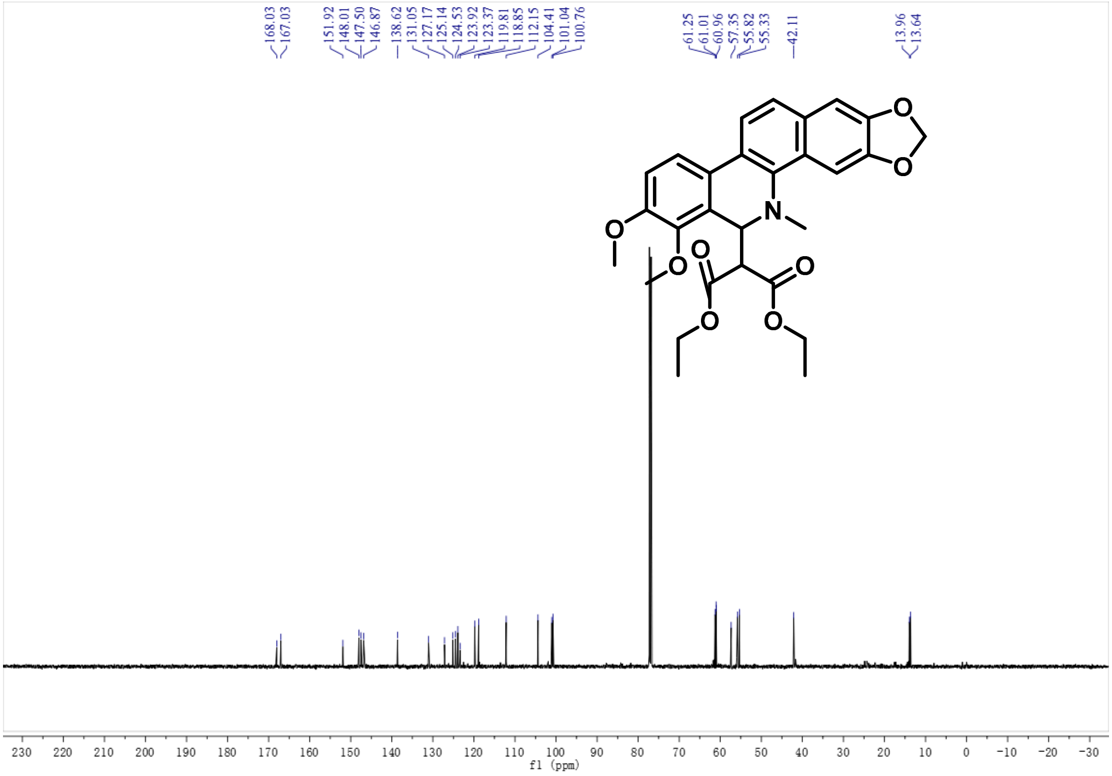

Figure S33. ^{13}C-NMR spectrum of **1j** (150 MHz, $CDCl_3$).

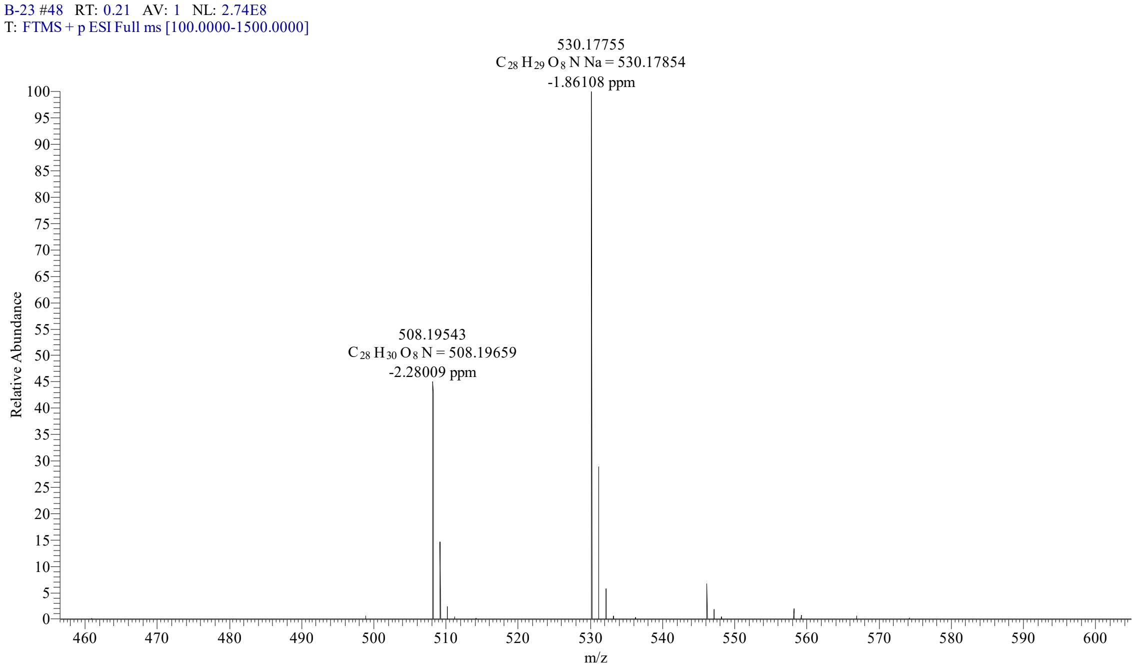

Figure S34. HR-ESI-MS spectrum of **1j.**

Compound **1k**

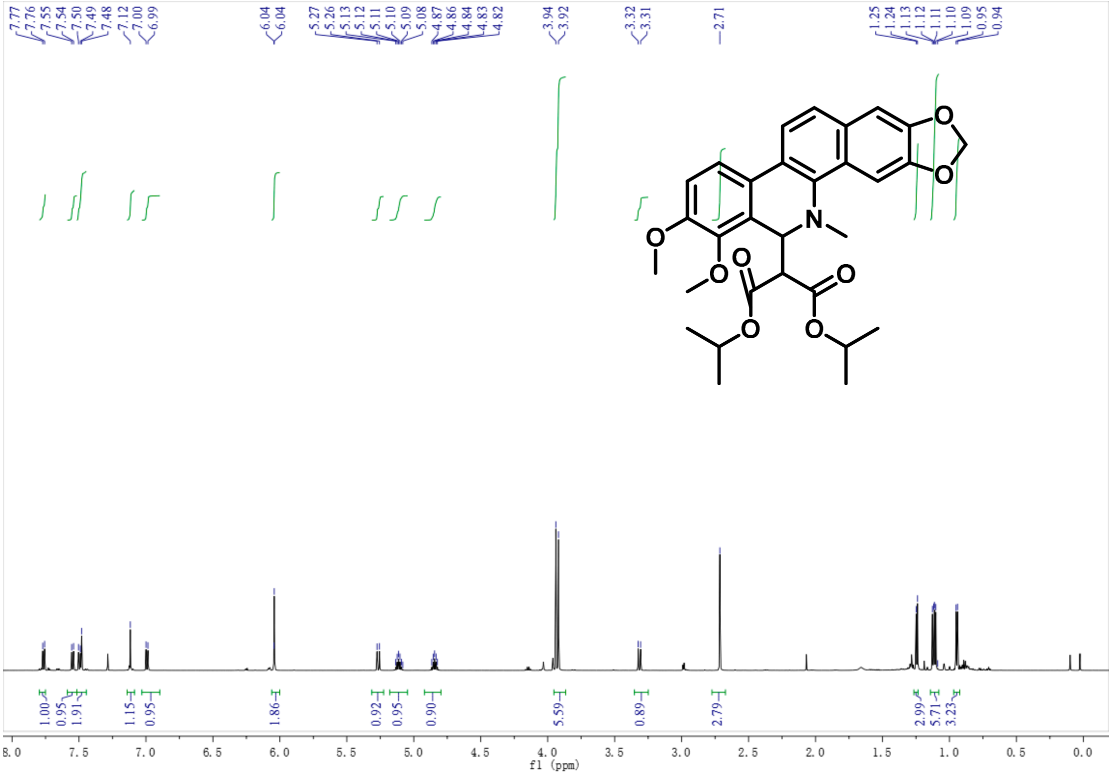

Figure S35. ^{1}H-NMR spectrum of **1k** (600 MHz, $CDCl_3$).

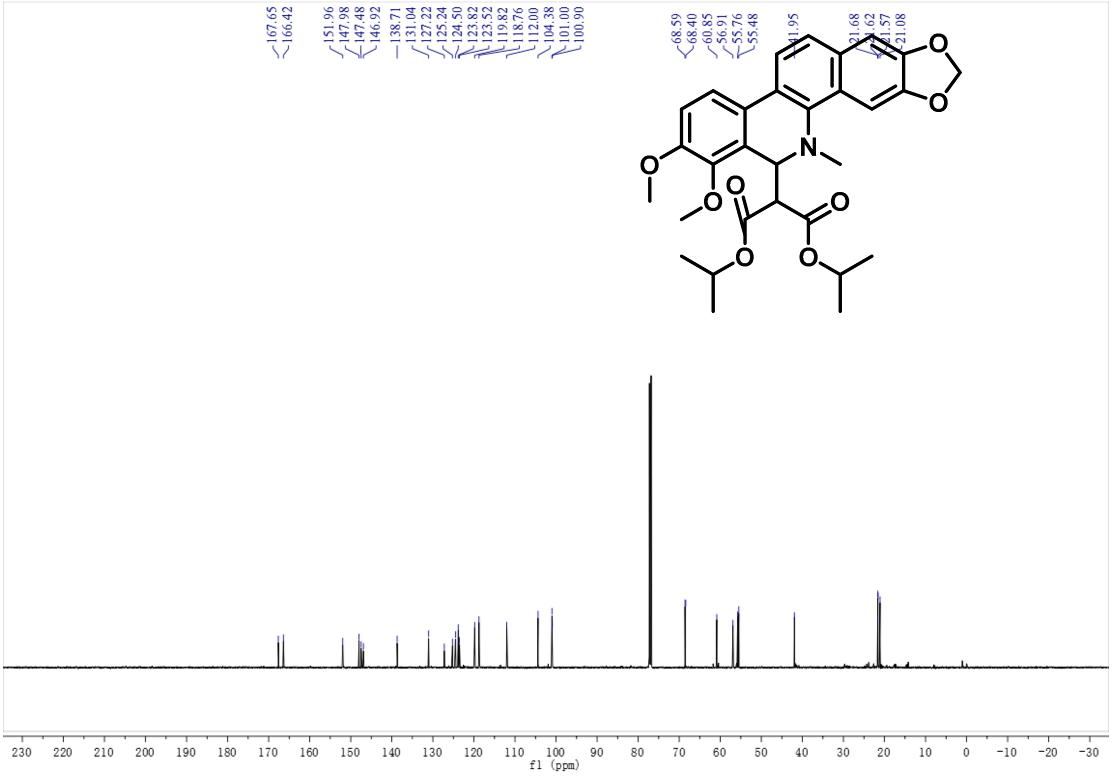

Figure S36. ^{13}C-NMR spectrum of **1k** (150 MHz, $CDCl_3$).

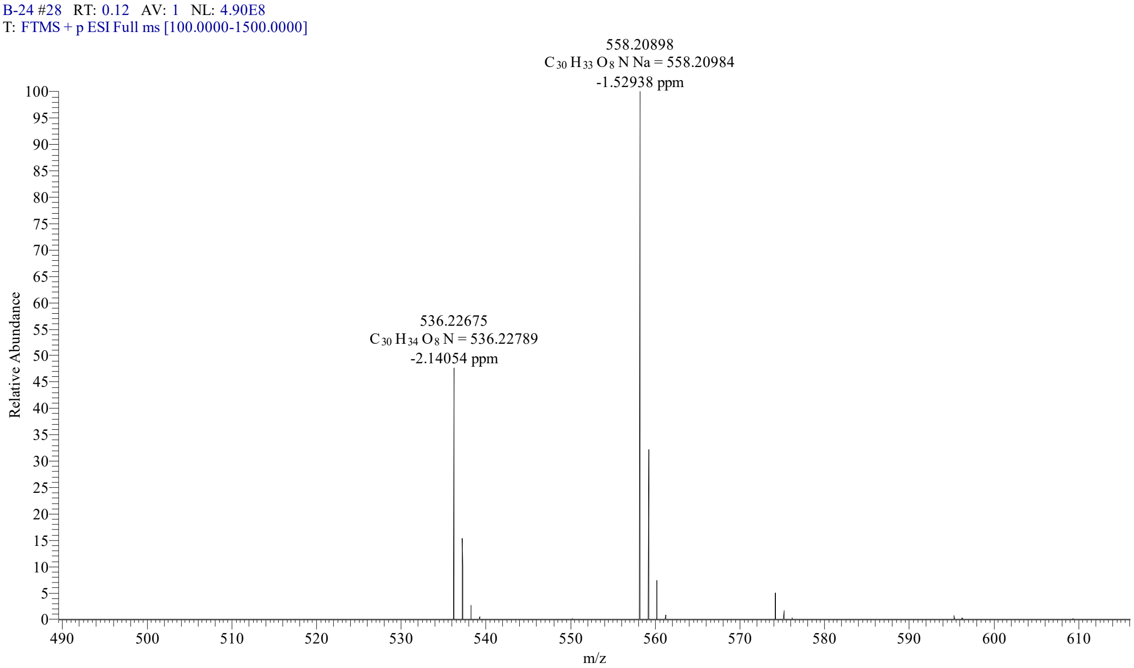

Figure S37. HR-ESI-MS spectrum of **1k.**

Compound **1l**

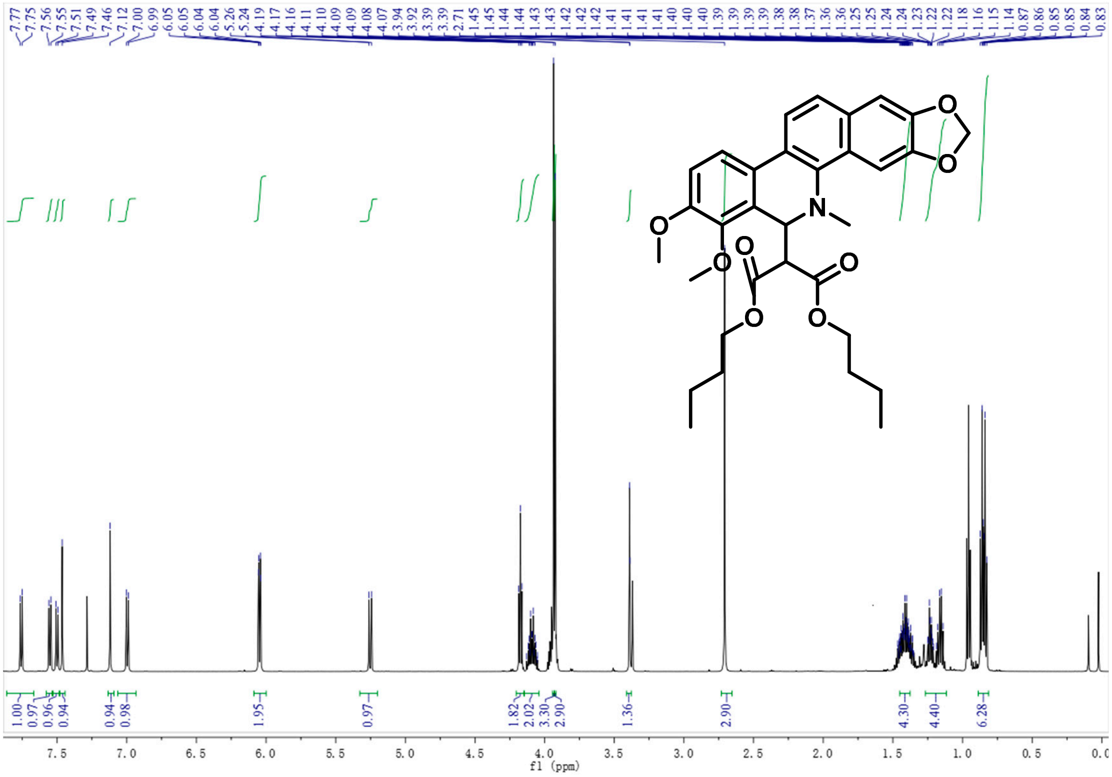

Figure S38. ^{1}H-NMR spectrum of **1l** (600 MHz, $CDCl_3$).

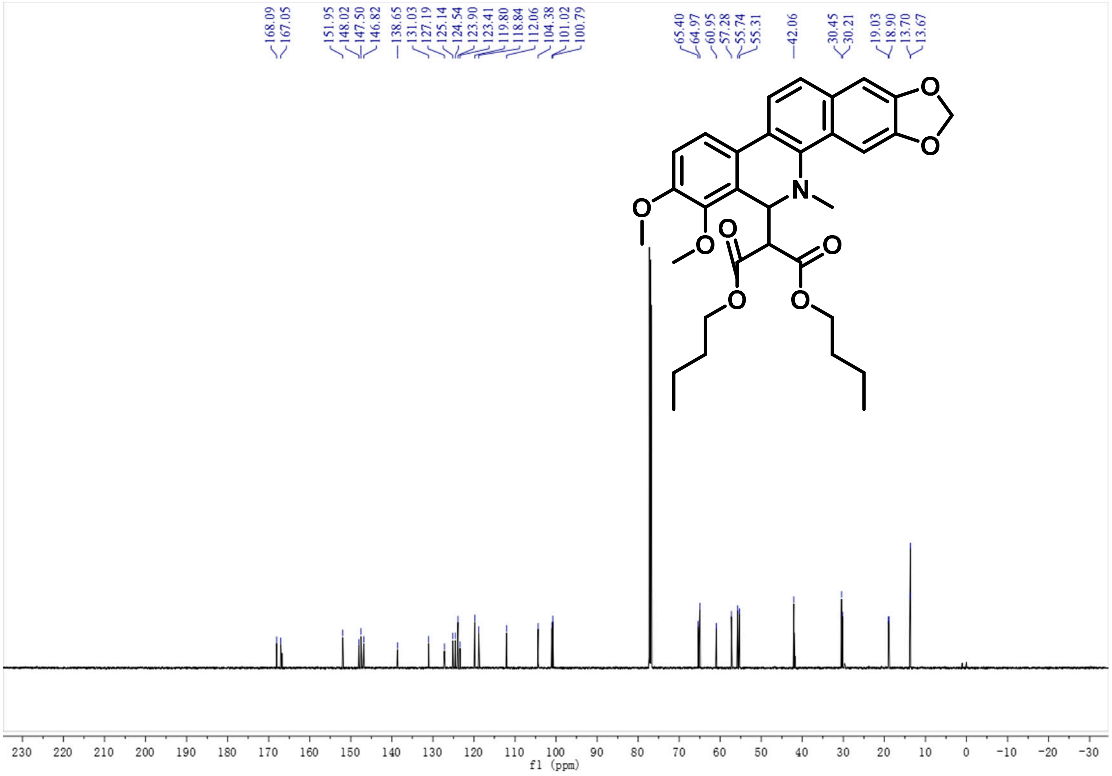

Figure S39. ^{13}C-NMR spectrum of **1l** (150 MHz, $CDCl_3$).

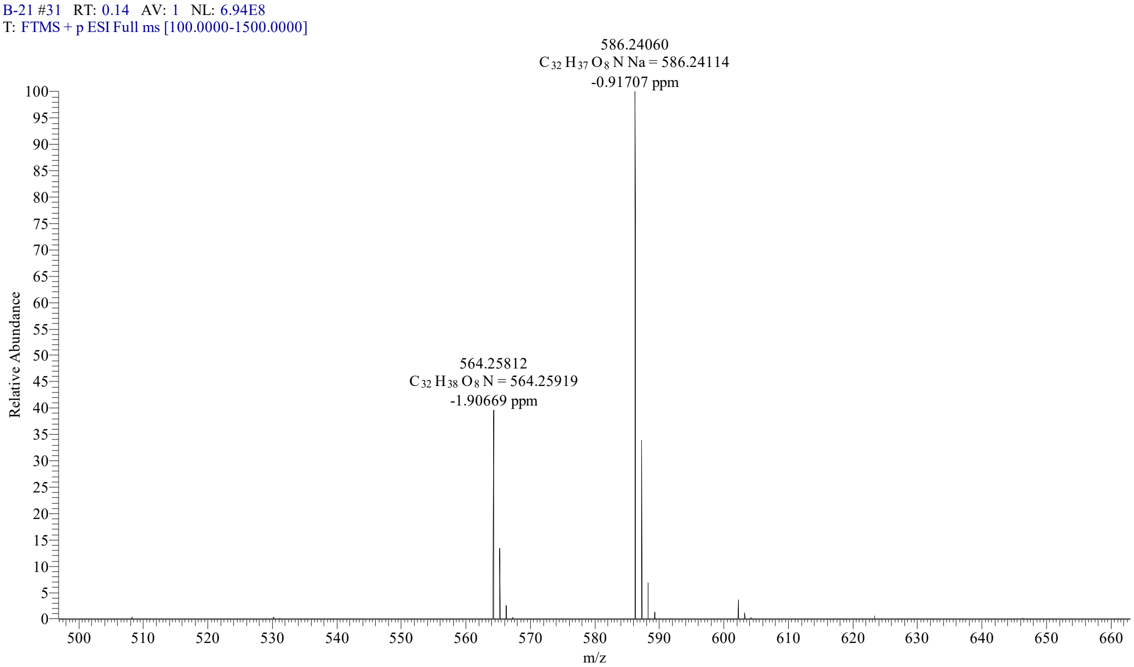

Figure S40. HR-ESI-MS spectrum of **1l.**

Compound **1m**

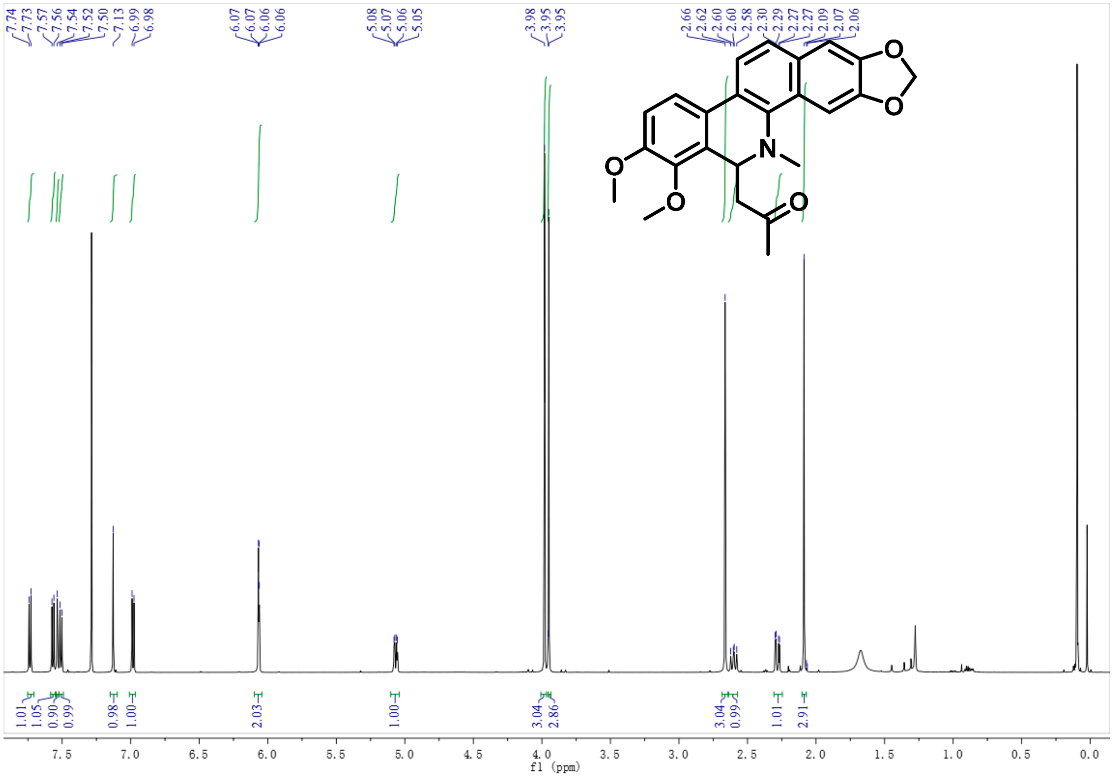

Figure S41. ^{1}H-NMR spectrum of **1m** (600 MHz, $CDCl_3$).

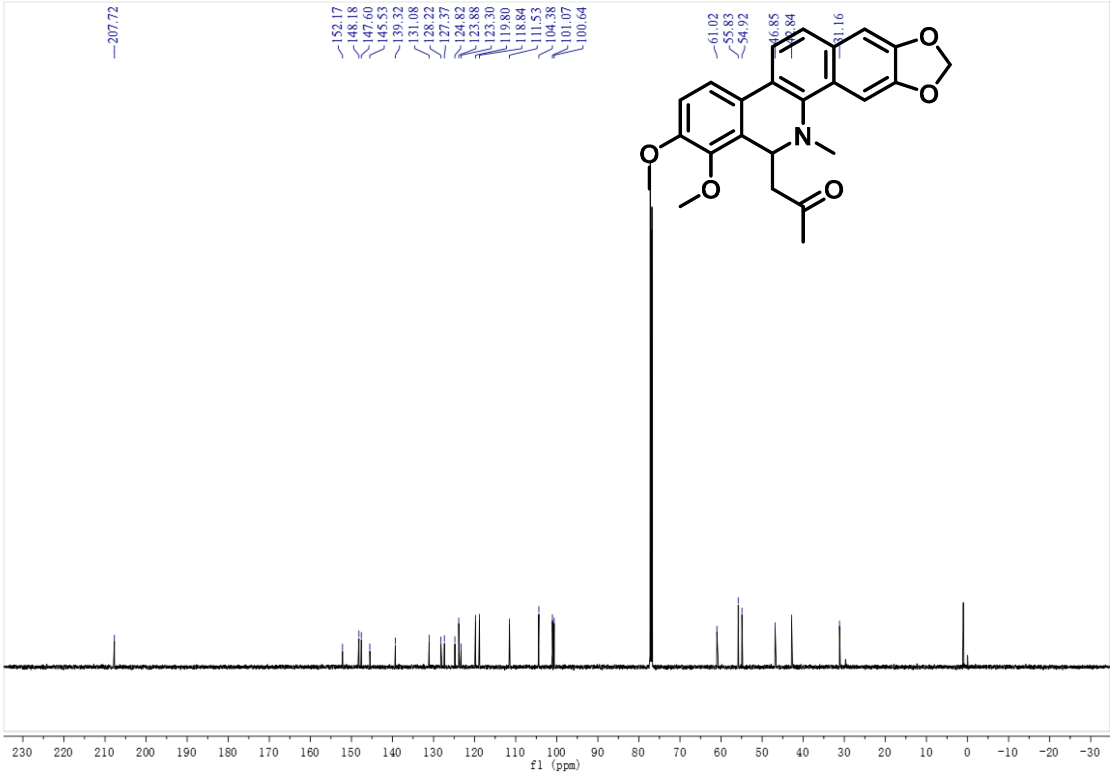

Figure S42. ^{13}C-NMR spectrum of **1m** (150 MHz, $CDCl_3$).

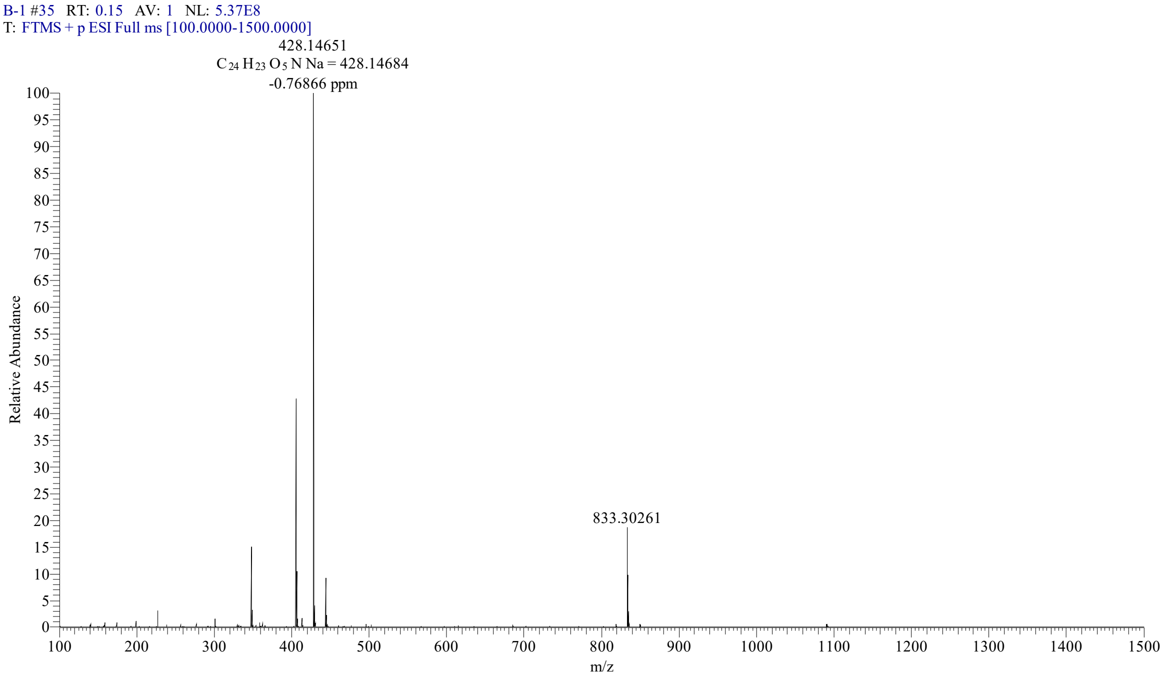

Figure S43. HR-ESI-MS spectrum of **1m.**

Compound **1n**

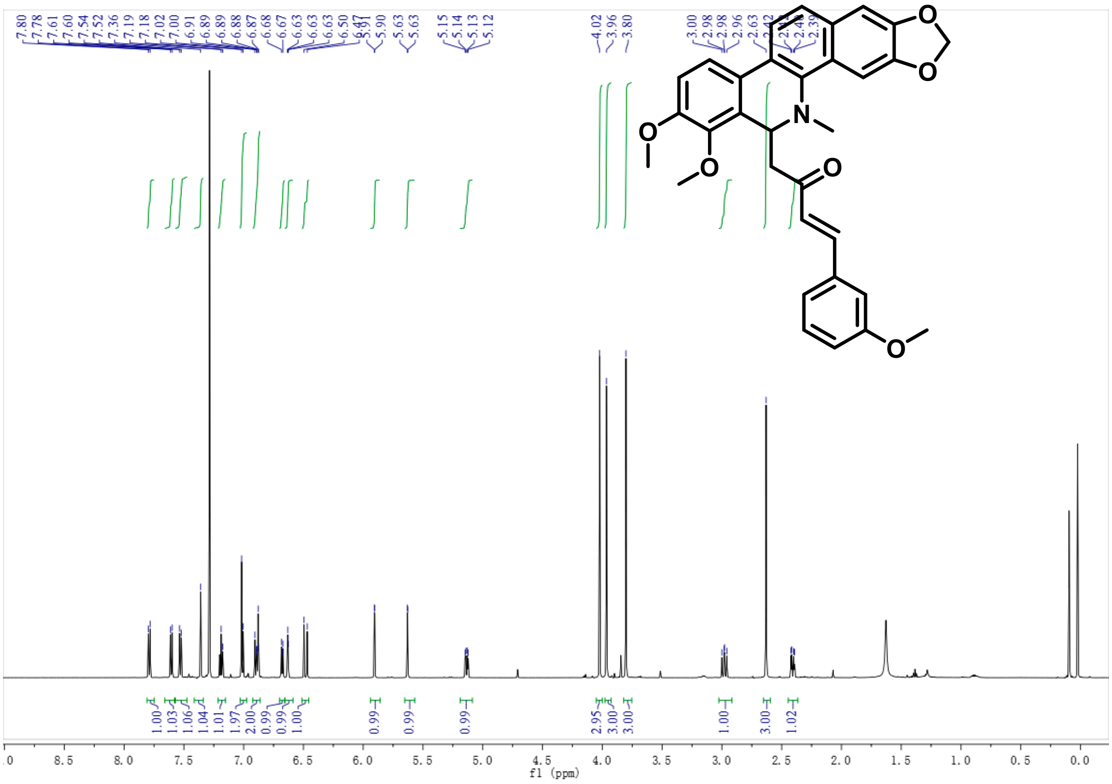

Figure S44. ^{1}H-NMR spectrum of **1n** (600 MHz, $CDCl_3$).

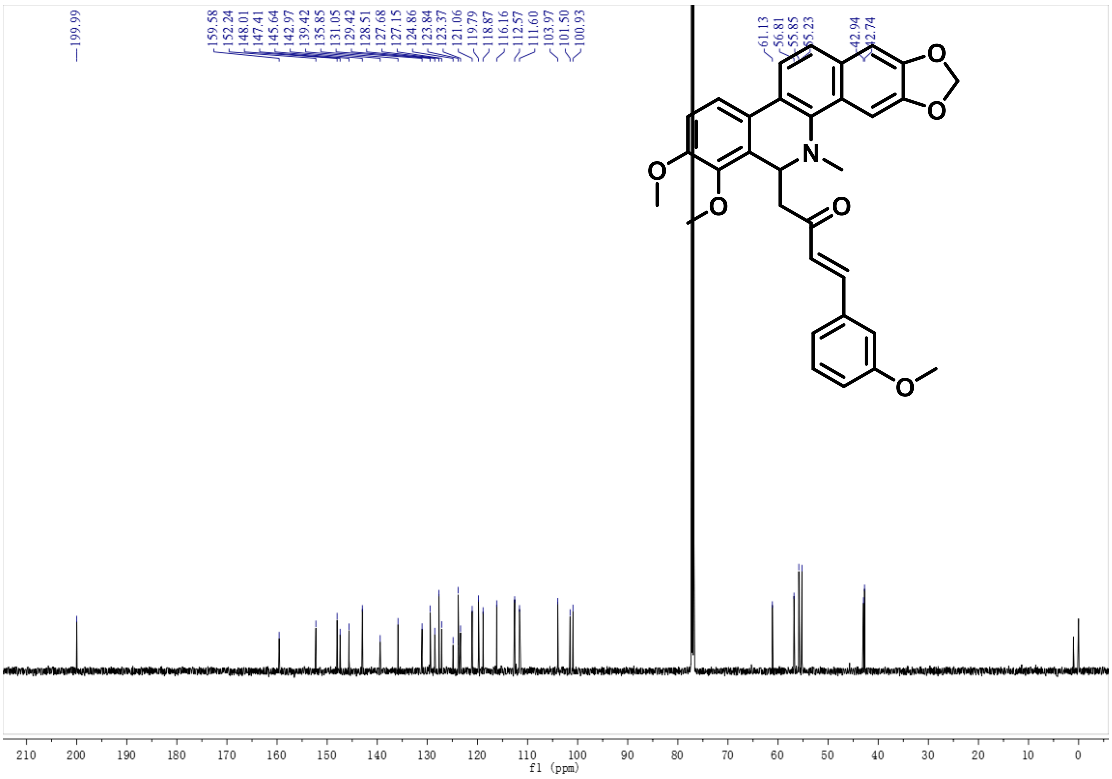

Figure S45. ^{13}C-NMR spectrum of **1n** (150 MHz, $CDCl_3$).

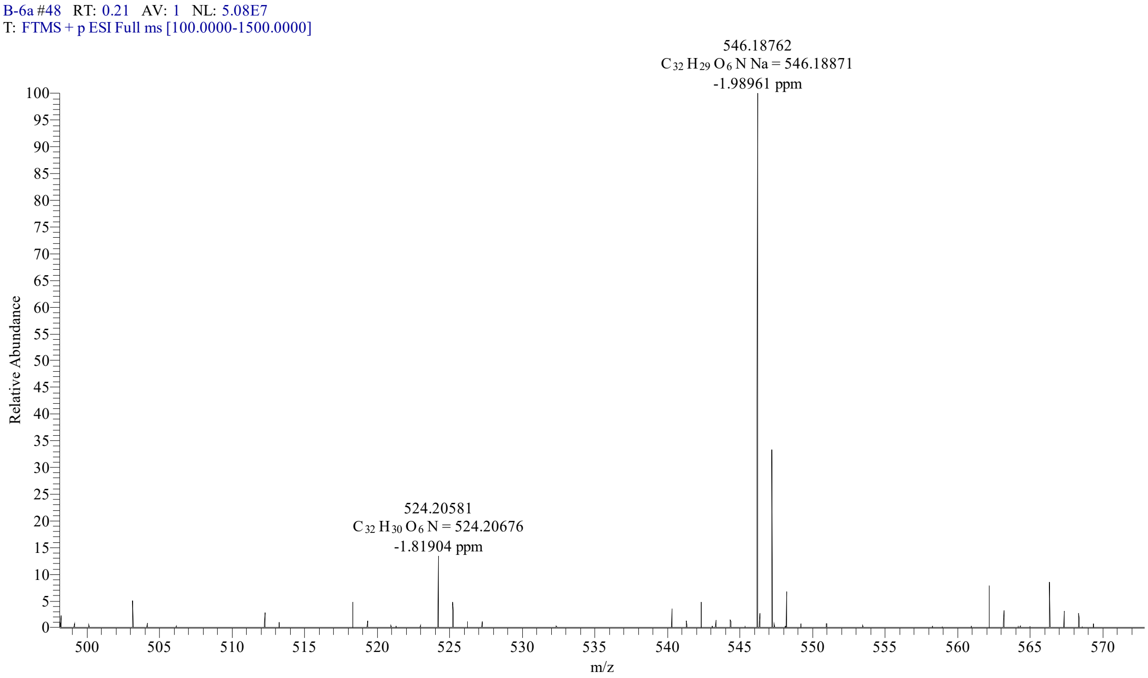

Figure S46. HR-ESI-MS spectrum of **1n.**

Compound **1o**

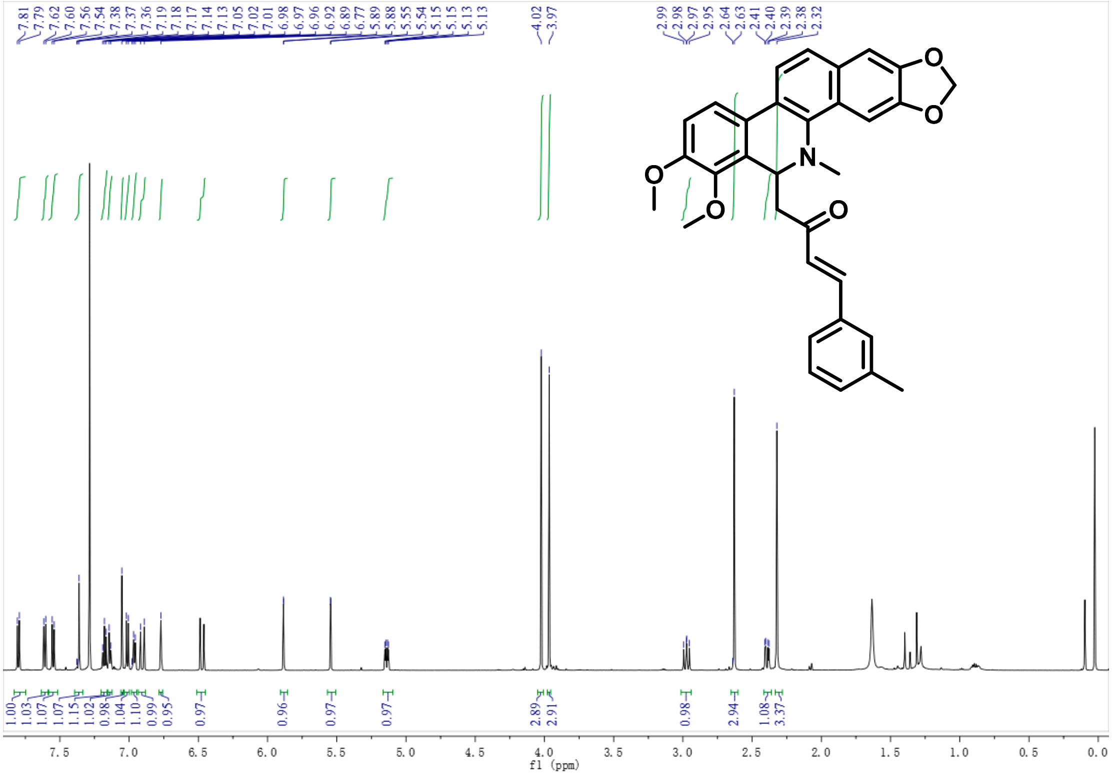

Figure S47. ^{1}H-NMR spectrum of **1o** (600 MHz, $CDCl_3$).

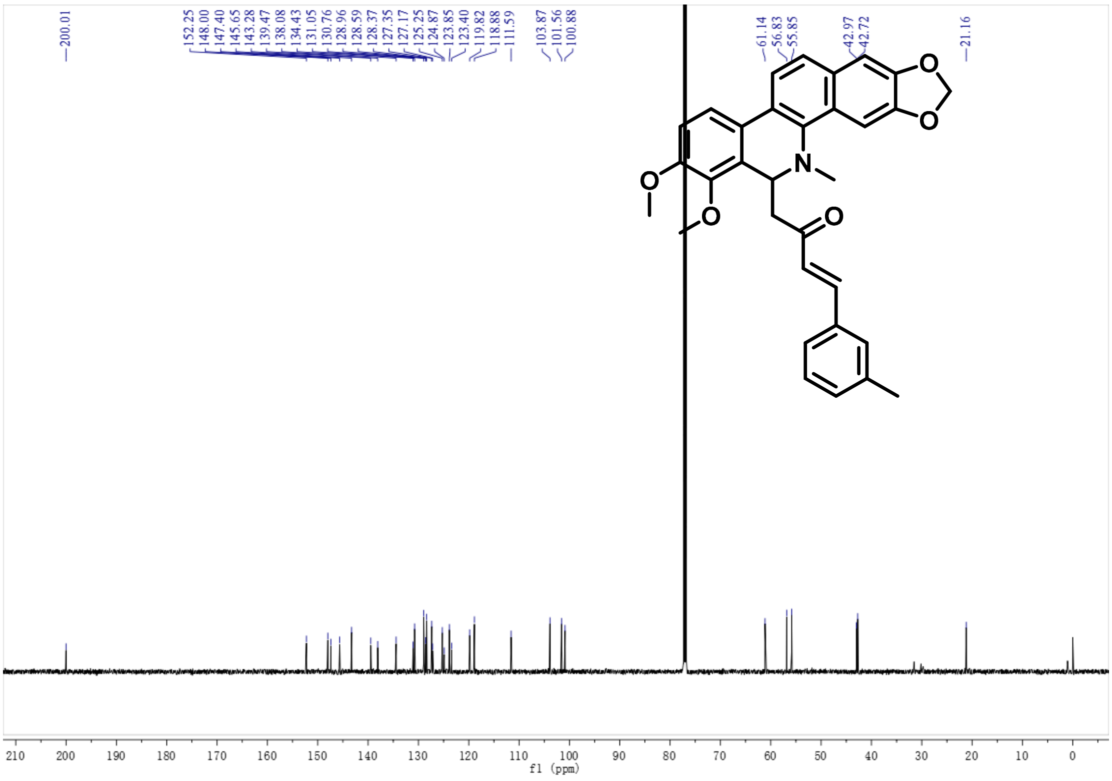

Figure S48. ^{13}C-NMR spectrum of **1o** (150 MHz, $CDCl_3$).

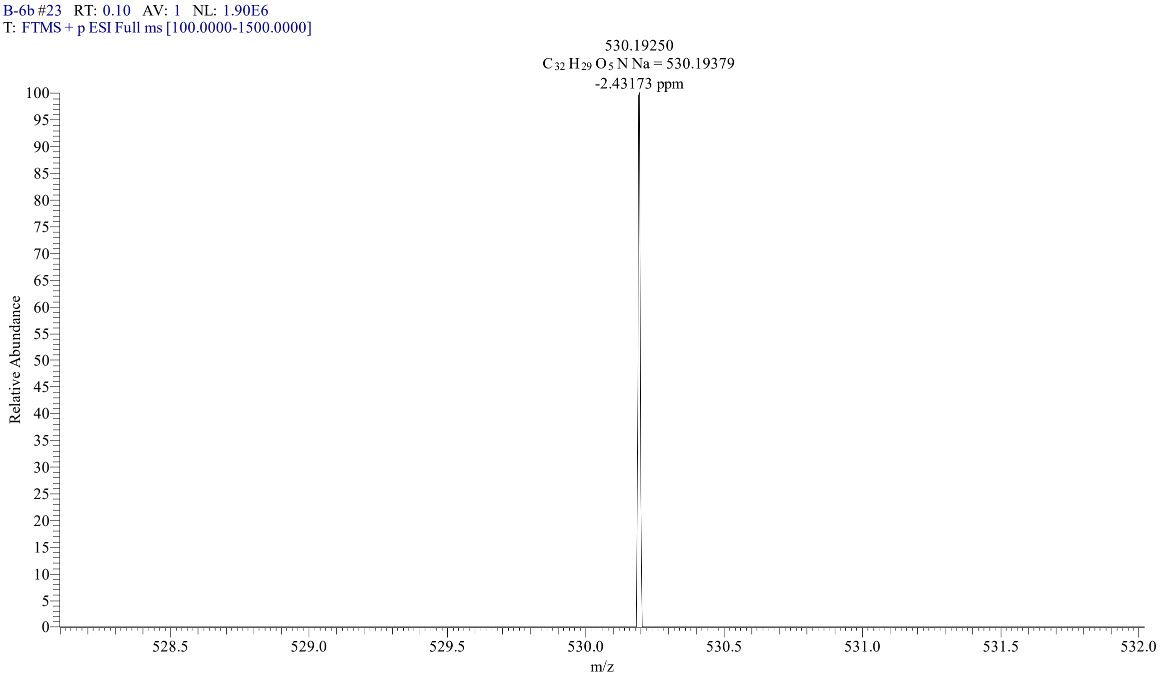

Figure S49. HR-ESI-MS spectrum of **1o.**

Compound **1p**

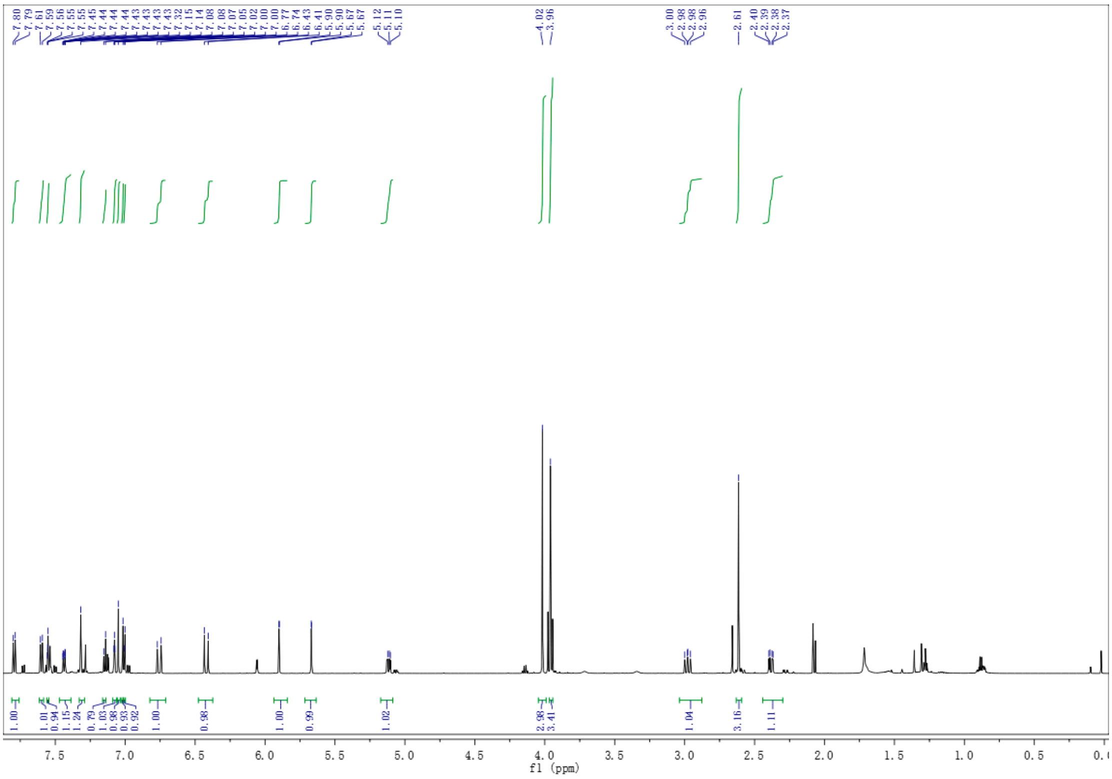

Figure S50. ^{1}H-NMR spectrum of **1p** (600 MHz, $CDCl_3$).

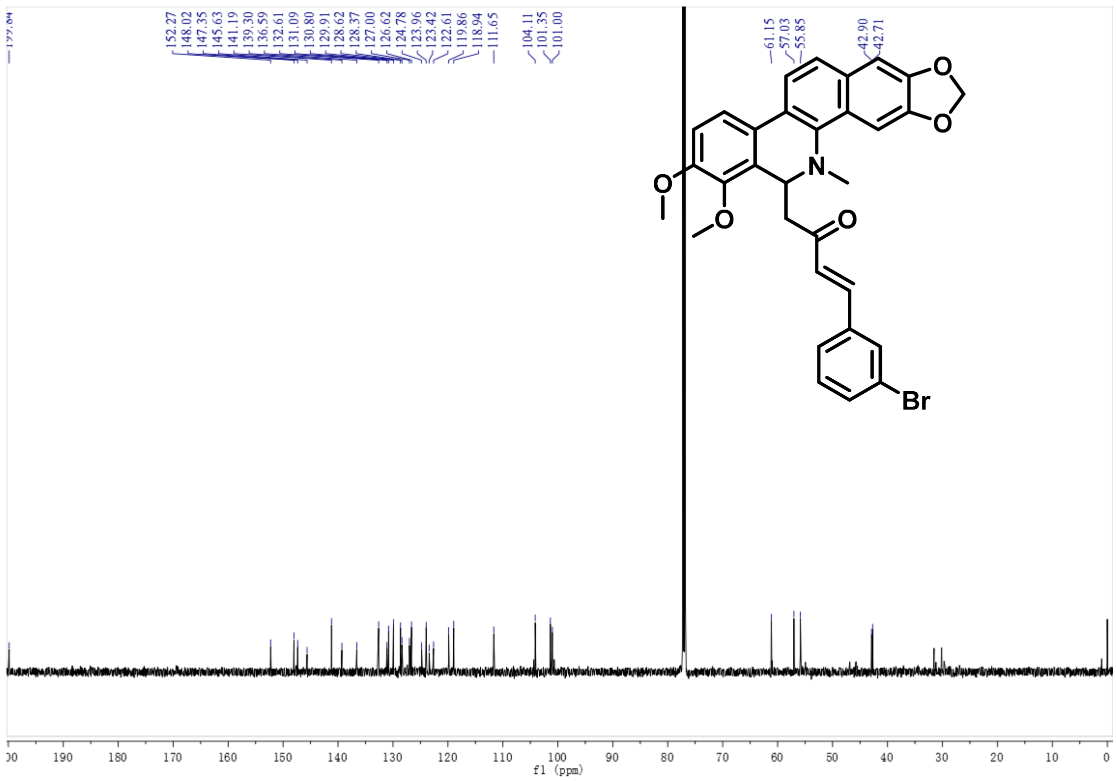

Figure S51. ^{13}C-NMR spectrum of **1p** (150 MHz, $CDCl_3$).

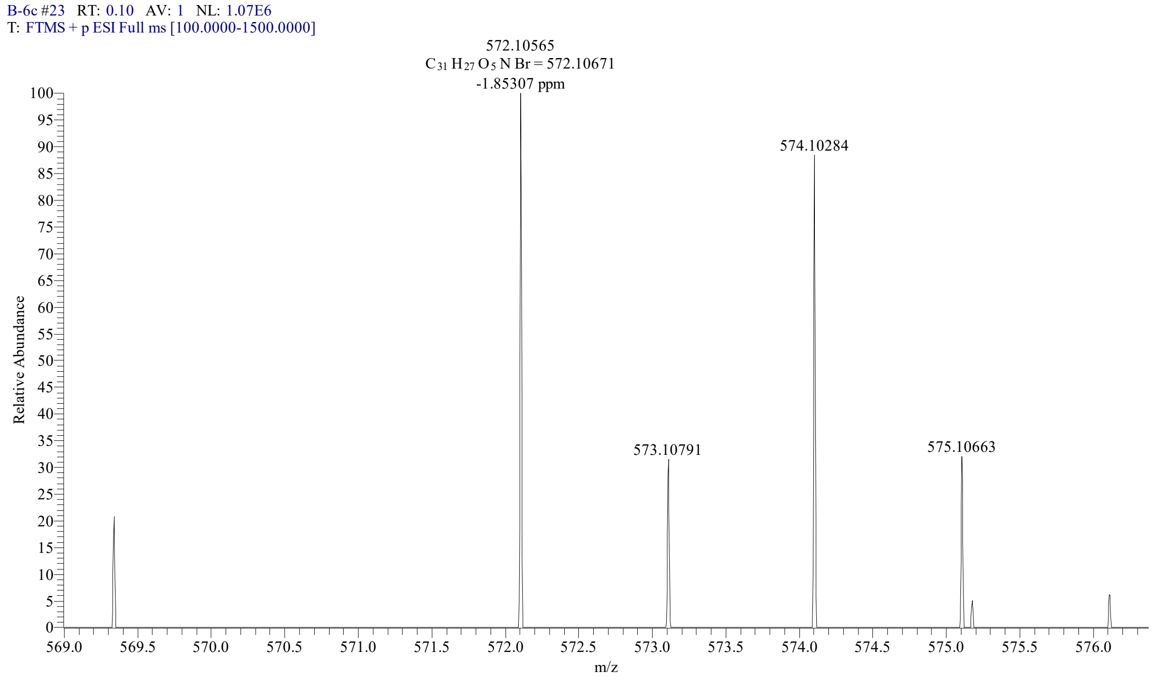

Figure S52. HR-ESI-MS spectrum of **1p.**

Compound **1q**

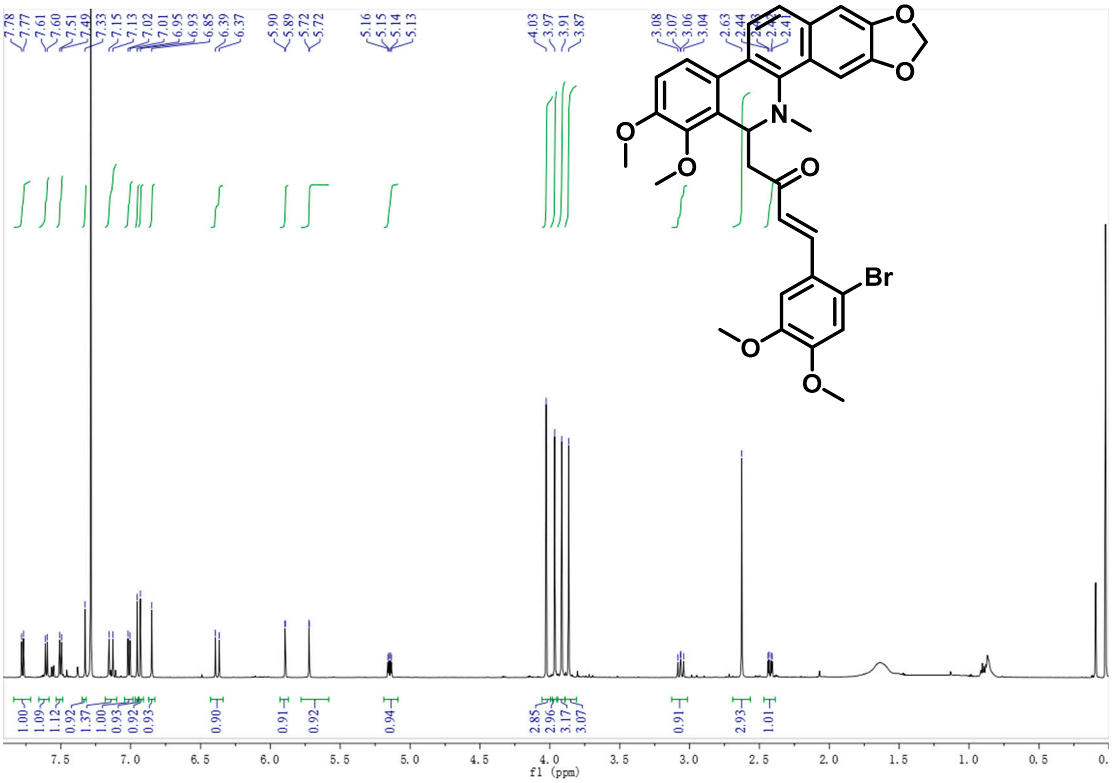

Figure S53. ^{1}H-NMR spectrum of **1q** (600 MHz, $CDCl_3$).

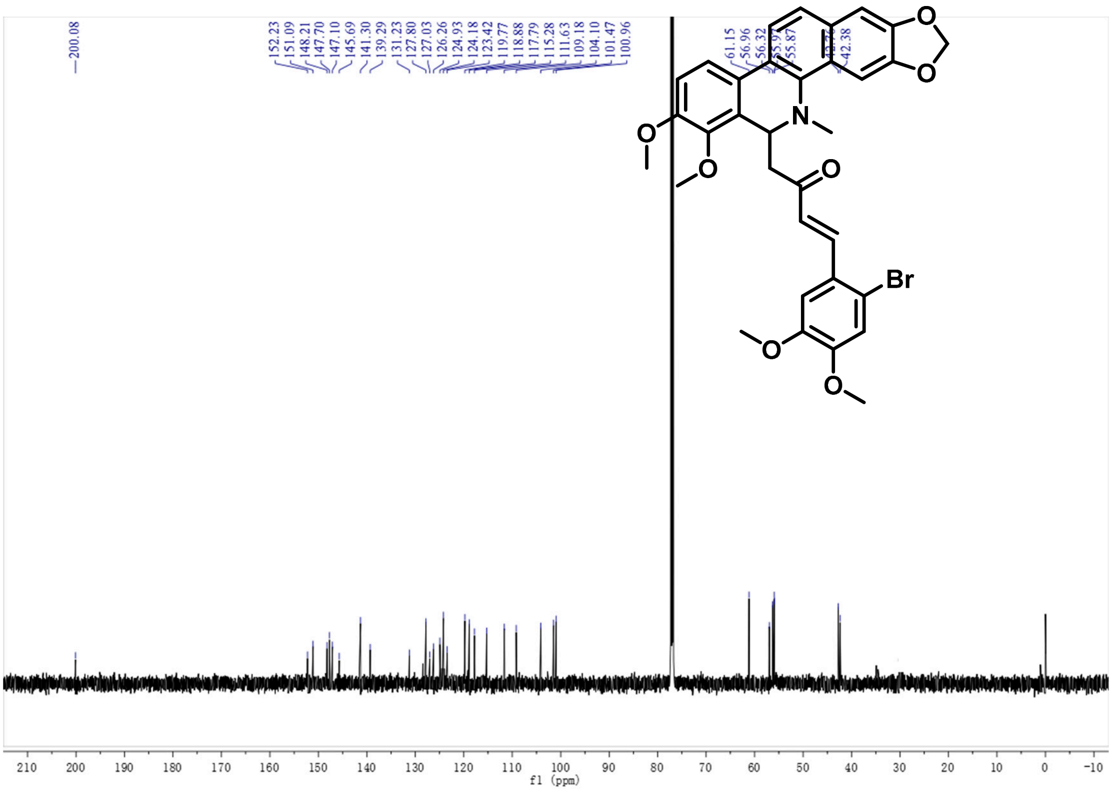

Figure S54. ^{13}C-NMR spectrum of **1q** (150 MHz, $CDCl_3$).

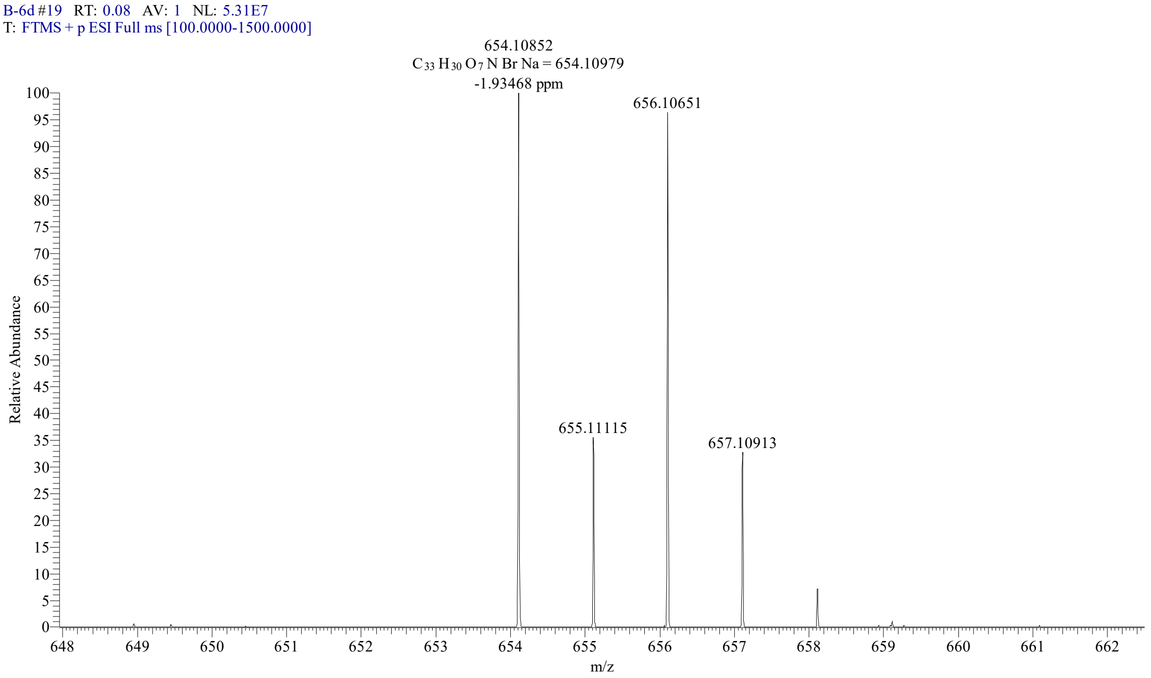

Figure S55. HR-ESI-MS spectrum of **1q.**

Compound **1r**

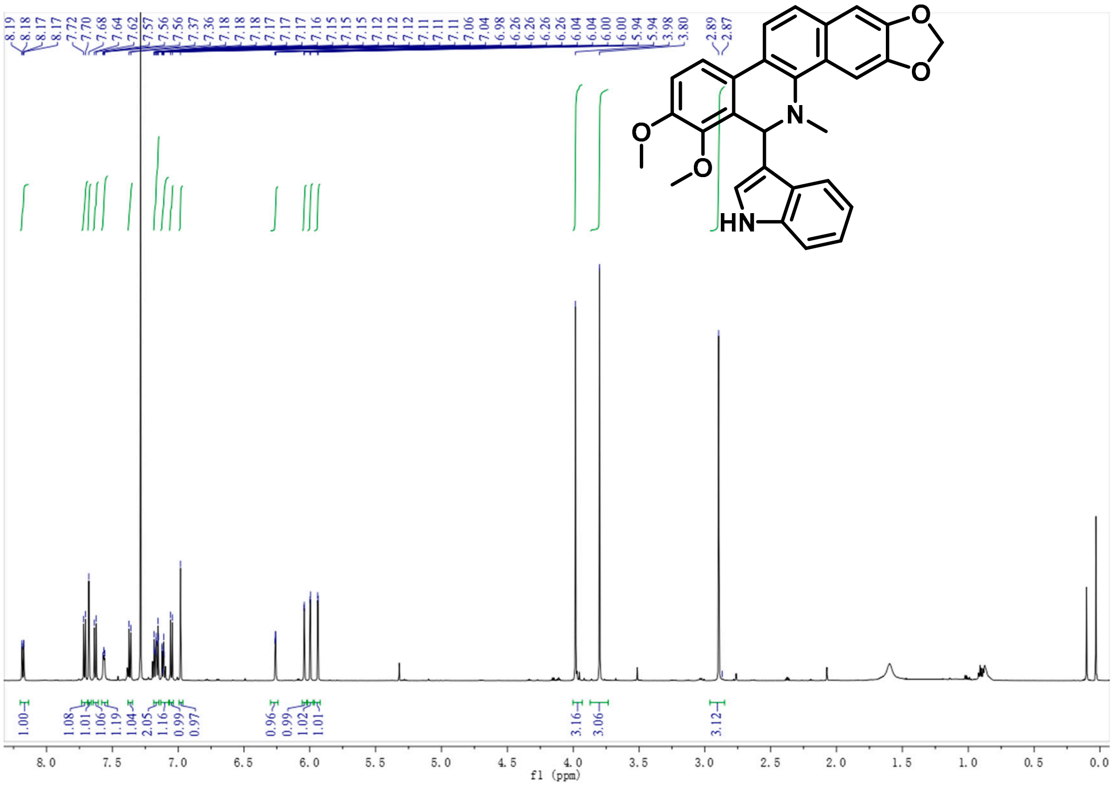

Figure S56. ^{1}H-NMR spectrum of **1r** (600 MHz, $CDCl_3$).

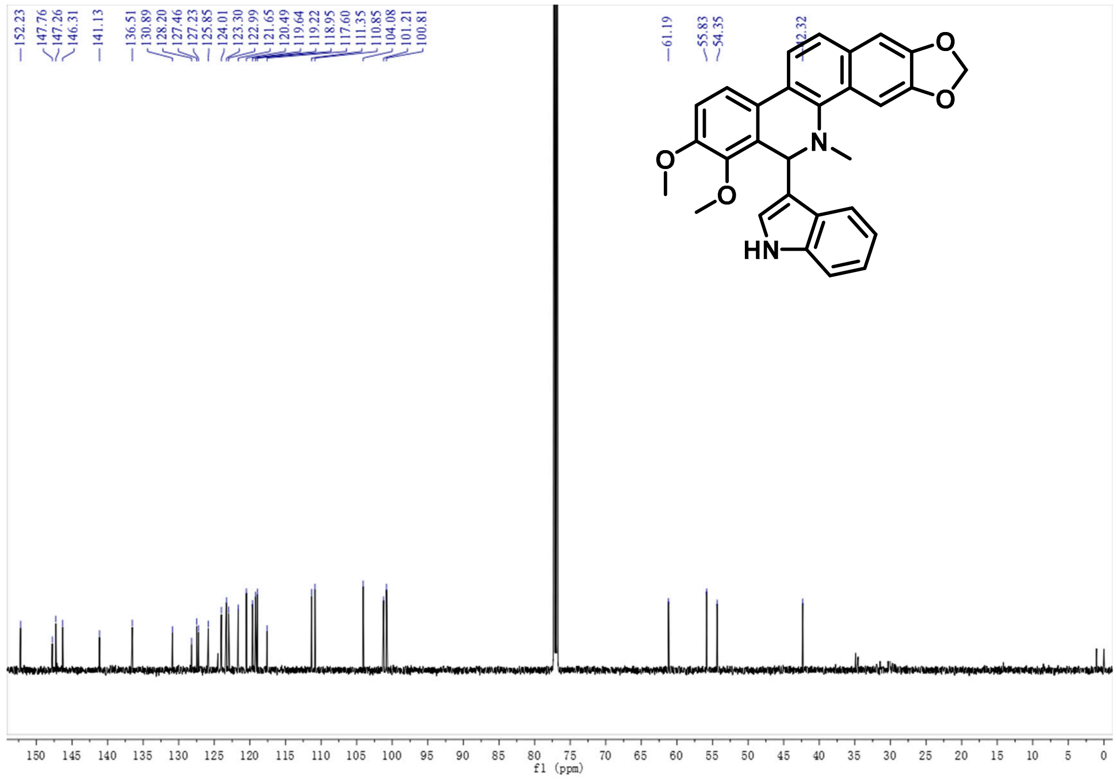

Figure S57. ^{13}C-NMR spectrum of **1r** (150 MHz, $CDCl_3$).

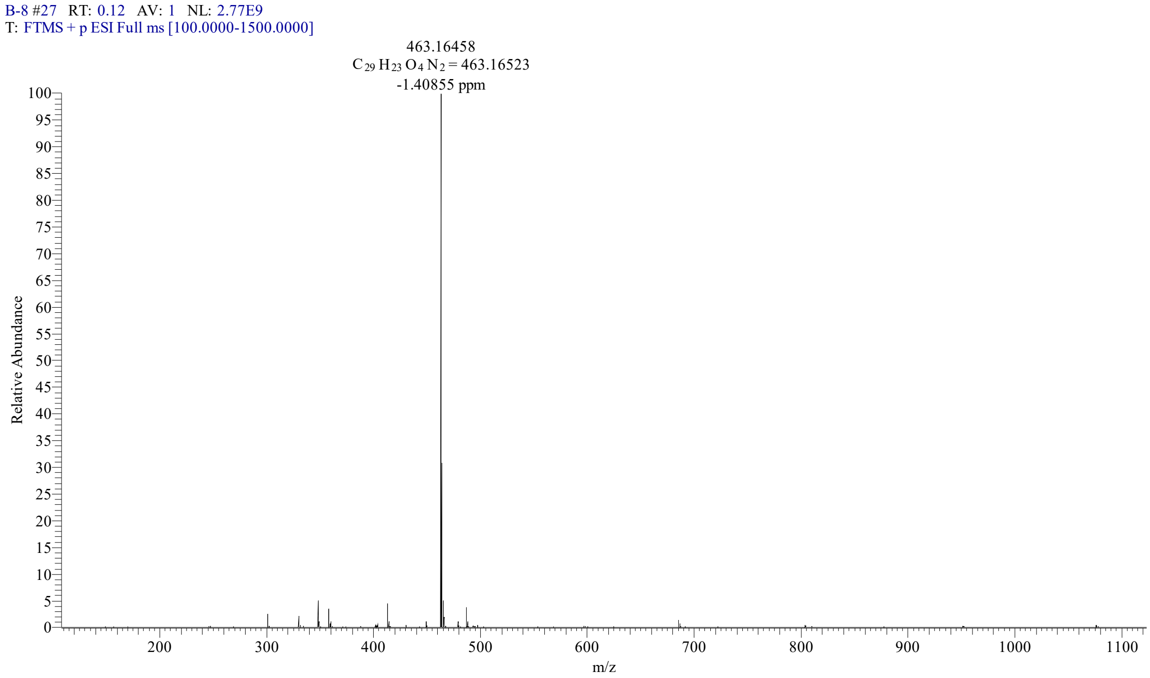

Figure S58. HR-ESI-MS spectrum of **1r.**

Compound **1s**

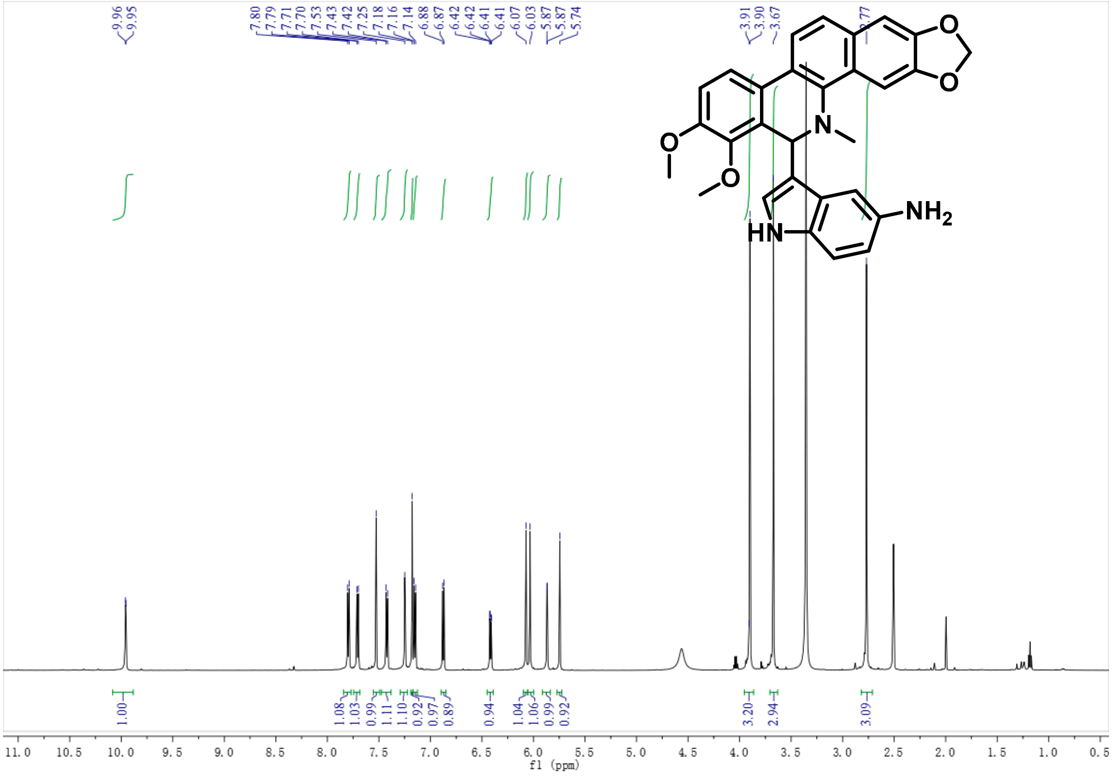

Figure S59. ^{1}H-NMR spectrum of **1s** (600 MHz, DMSO-d_6).

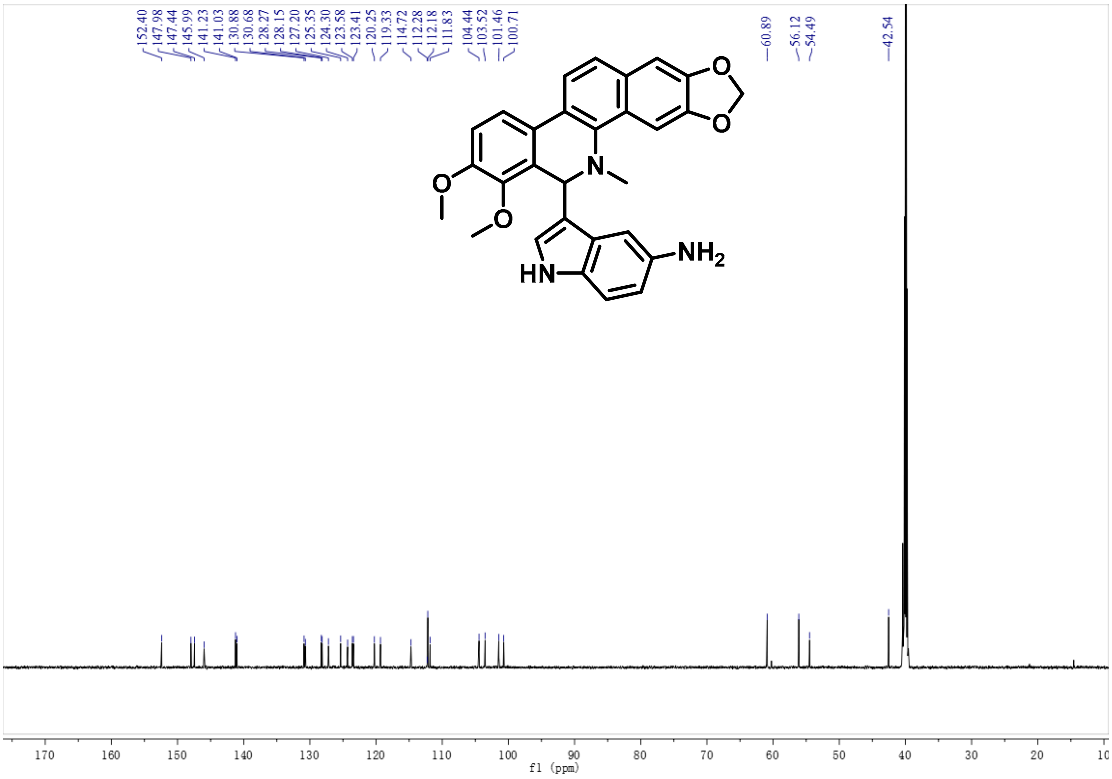

Figure S60. ^{13}C-NMR spectrum of **1s** (150 MHz, DMSO-d_6).

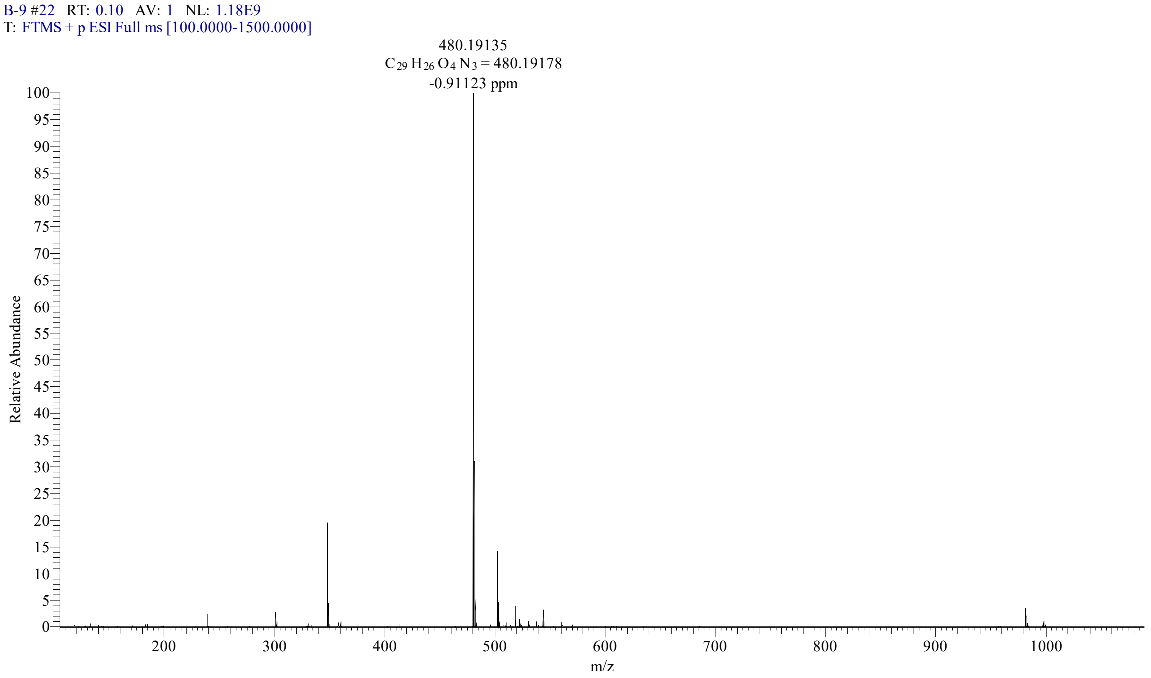

Figure S61. HR-ESI-MS spectrum of **1s.**

Compound **1t**

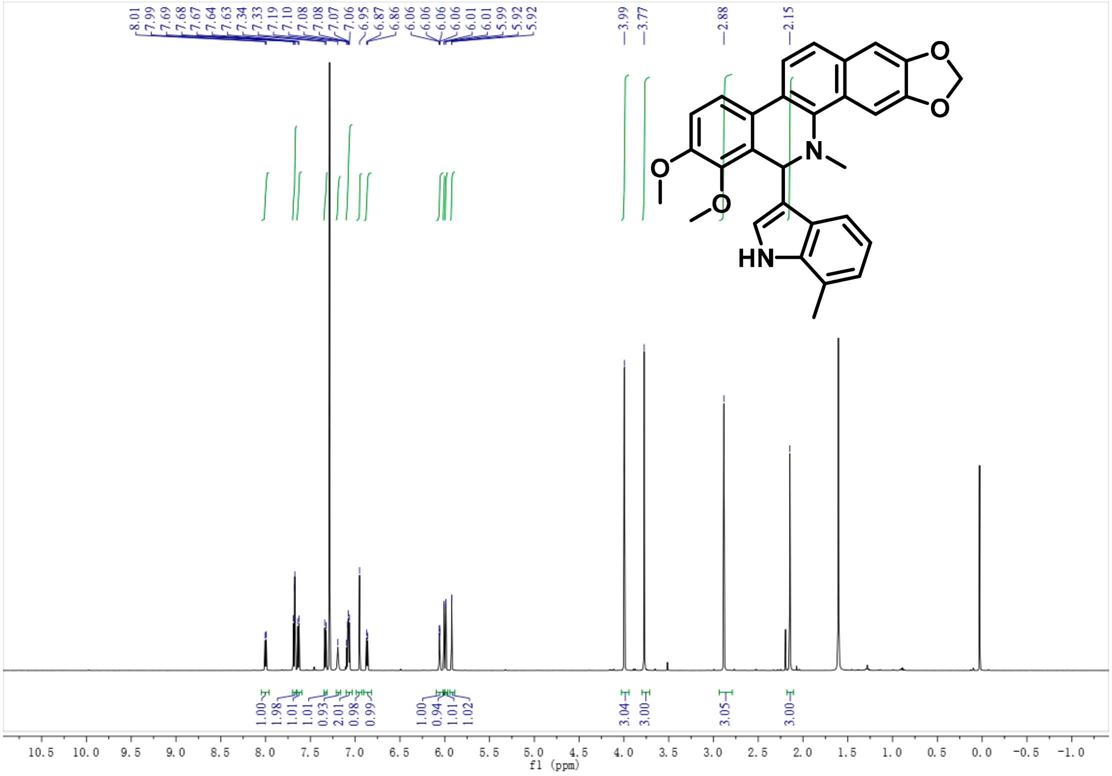

Figure S62. ^{1}H-NMR spectrum of **1t** (600 MHz, $CDCl_3$).

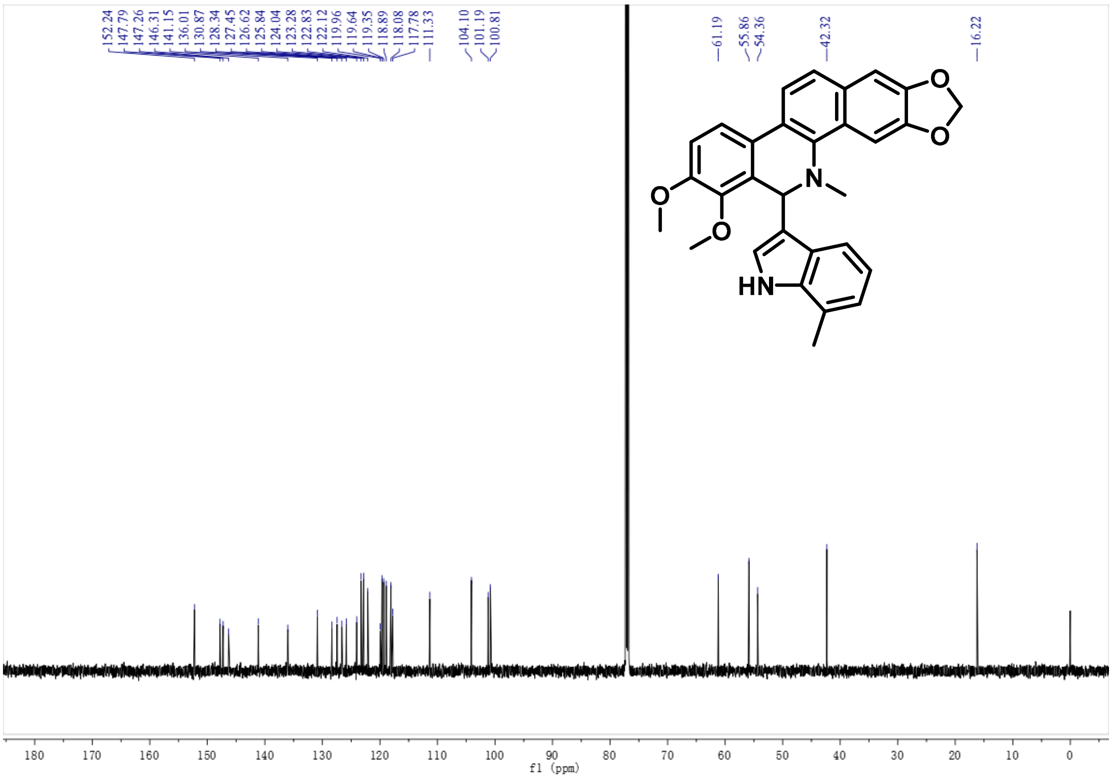

Figure S63. ^{13}C-NMR spectrum of **1t** (150 MHz, $CDCl_3$).

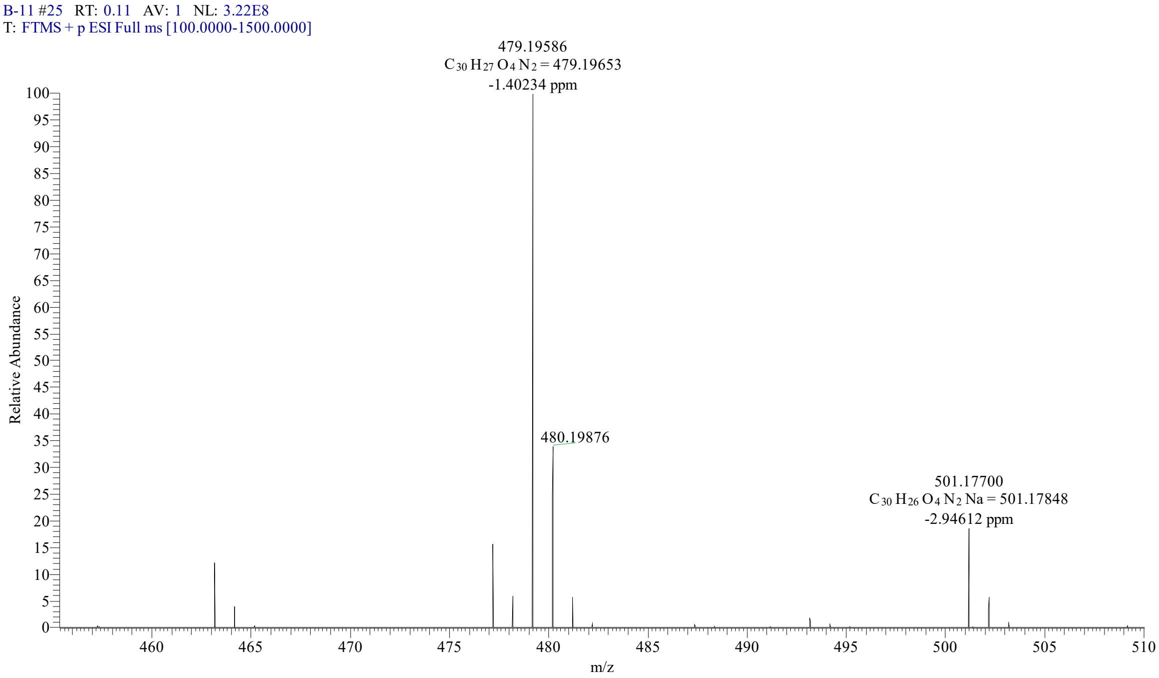

Figure S64. HR-ESI-MS spectrum of **1t.**

Compound **1u**

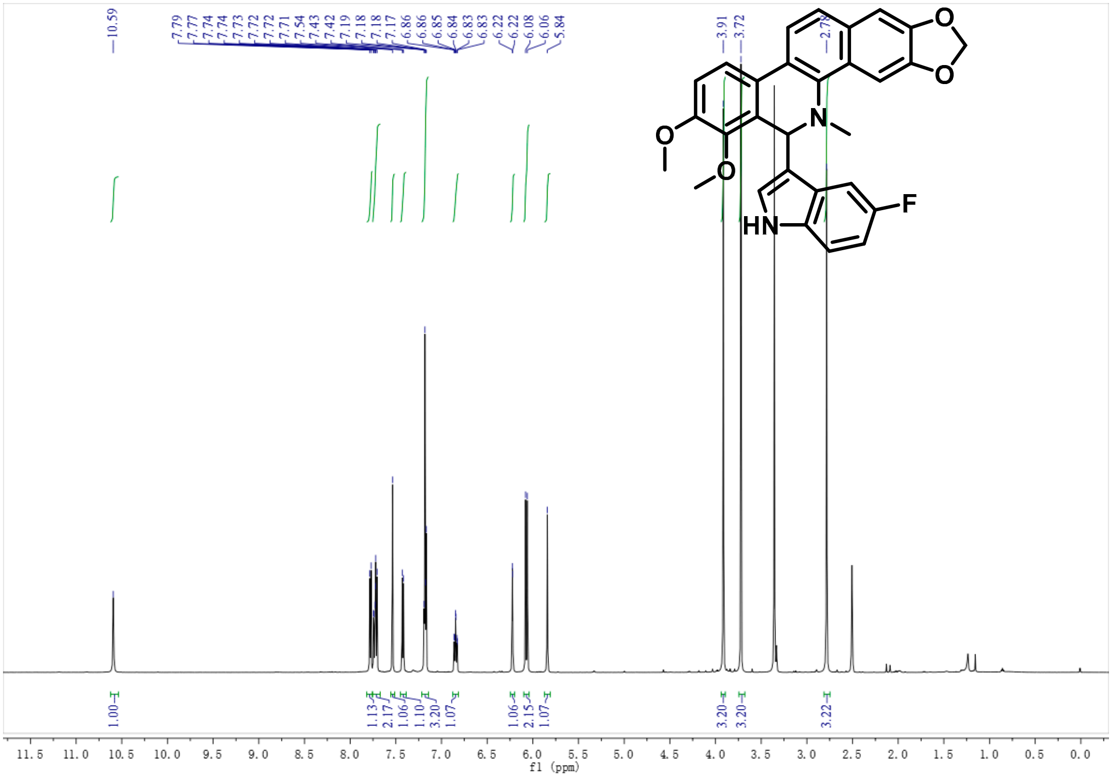

Figure S65. ^{1}H-NMR spectrum of **1u** (600 MHz, DMSO-d_6).

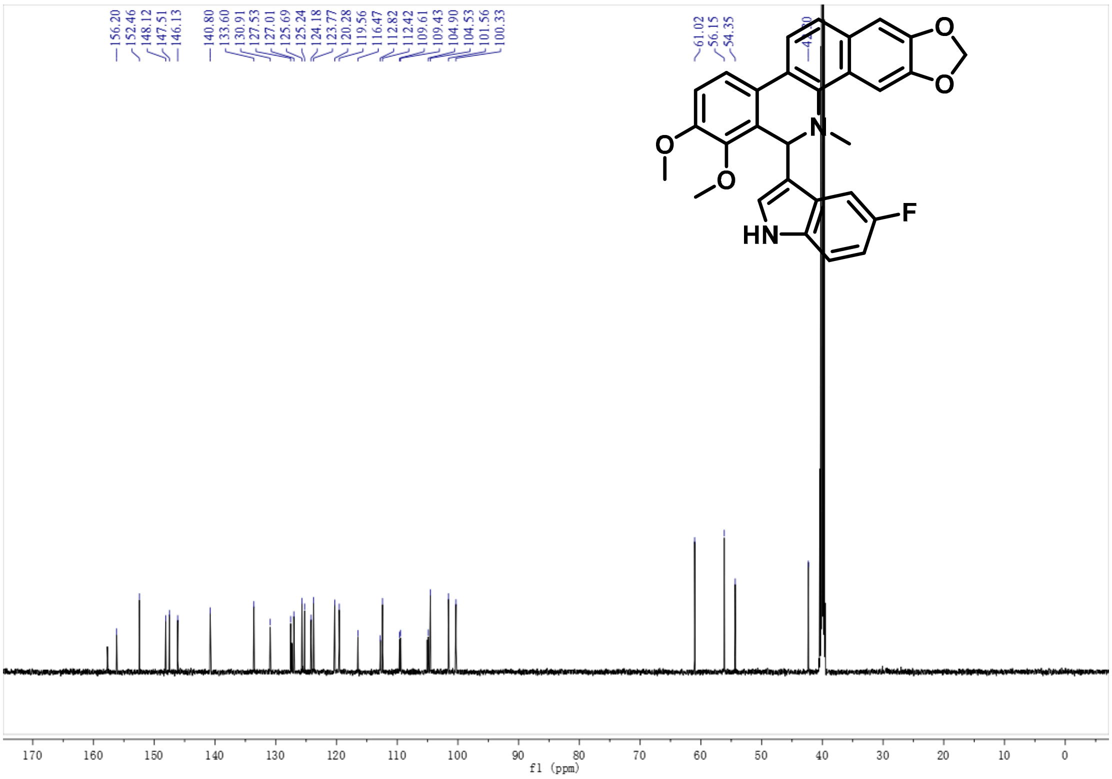

Figure S66. ^{13}C-NMR spectrum of **1u** (150 MHz, DMSO-d_6).

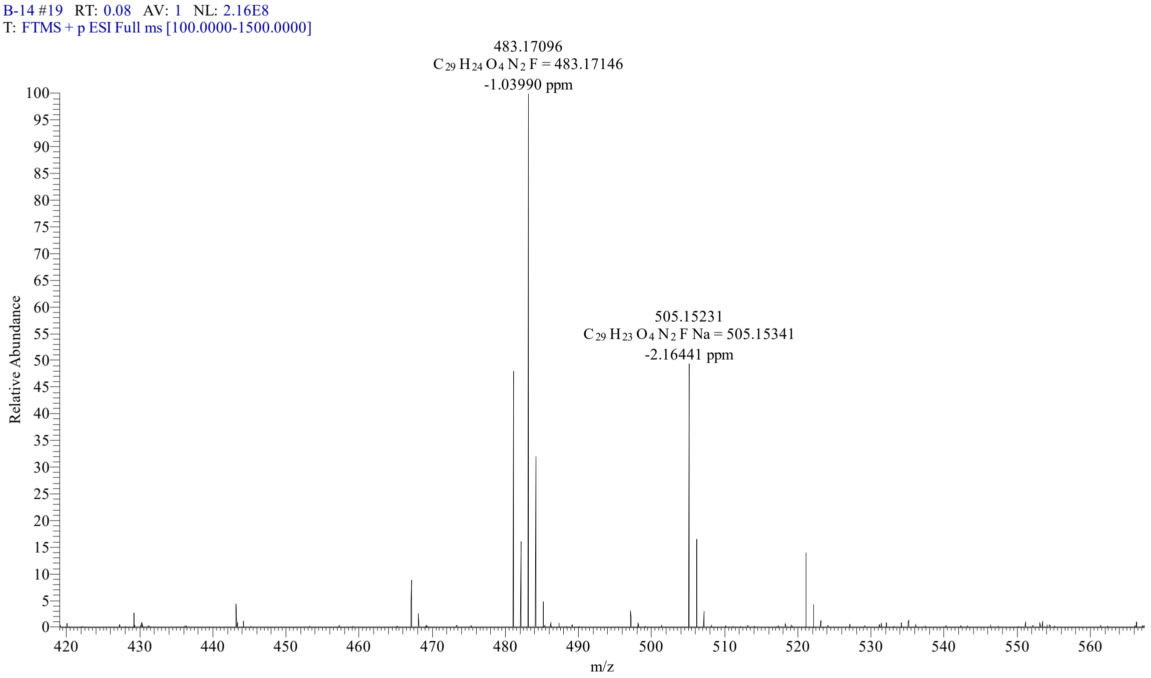

Figure S67. HR-ESI-MS spectrum of **1u.**

Compound **2a**

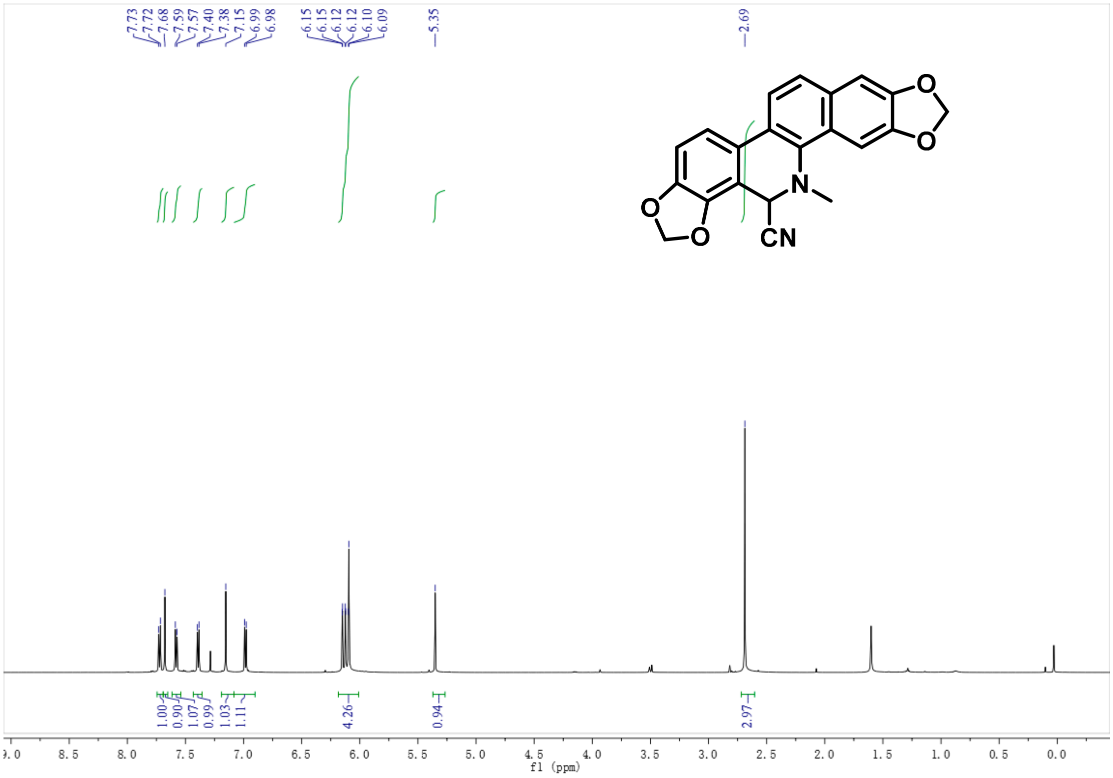

Figure S68. ^{1}H-NMR spectrum of **2a** (600 MHz, $CDCl_3$).

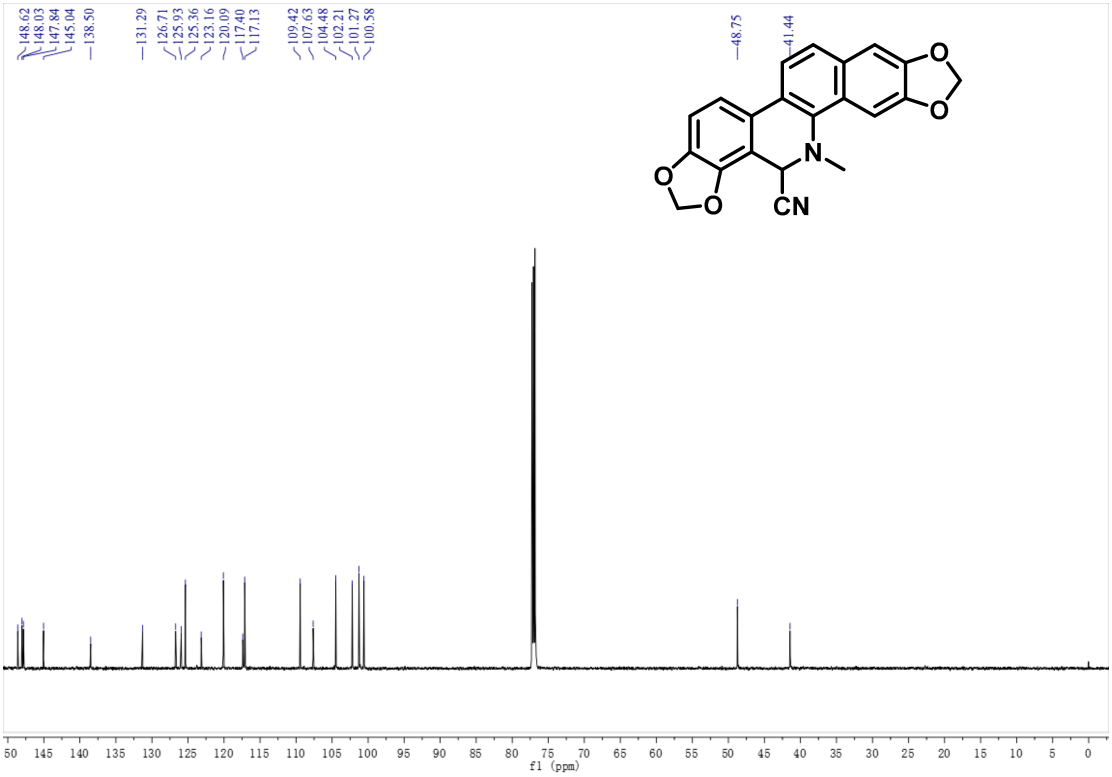

Figure S69. ^{13}C-NMR spectrum of **2a** (150 MHz, $CDCl_3$).

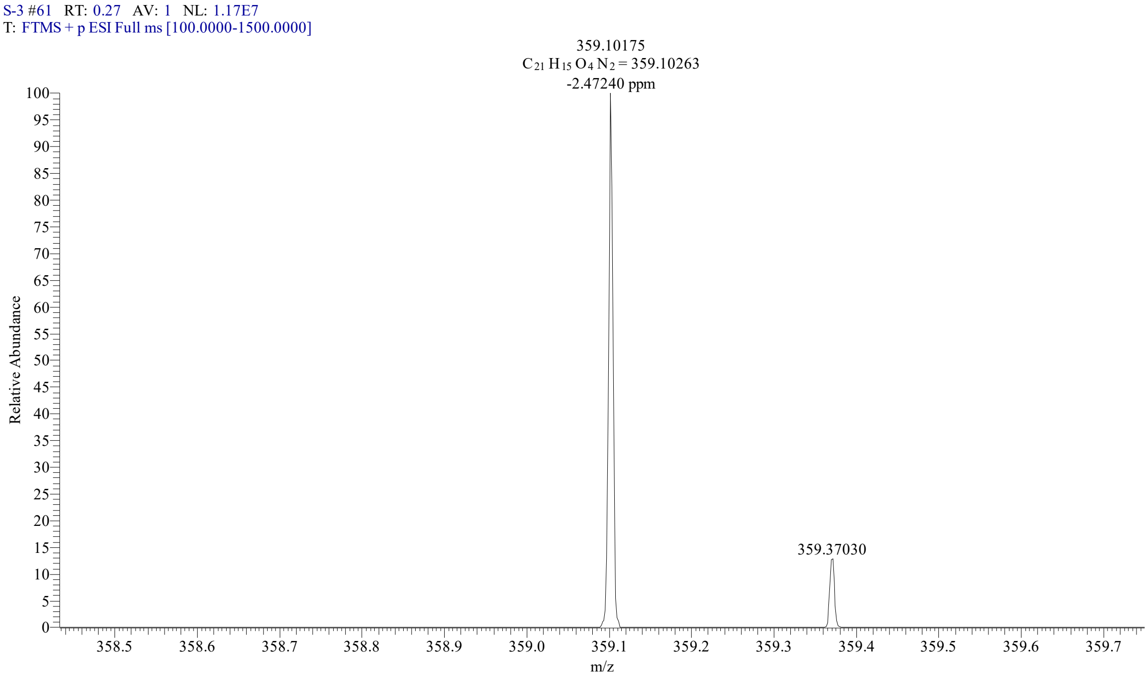

Figure S70. HR-ESI-MS spectrum of **2a.**

Compound **2b**

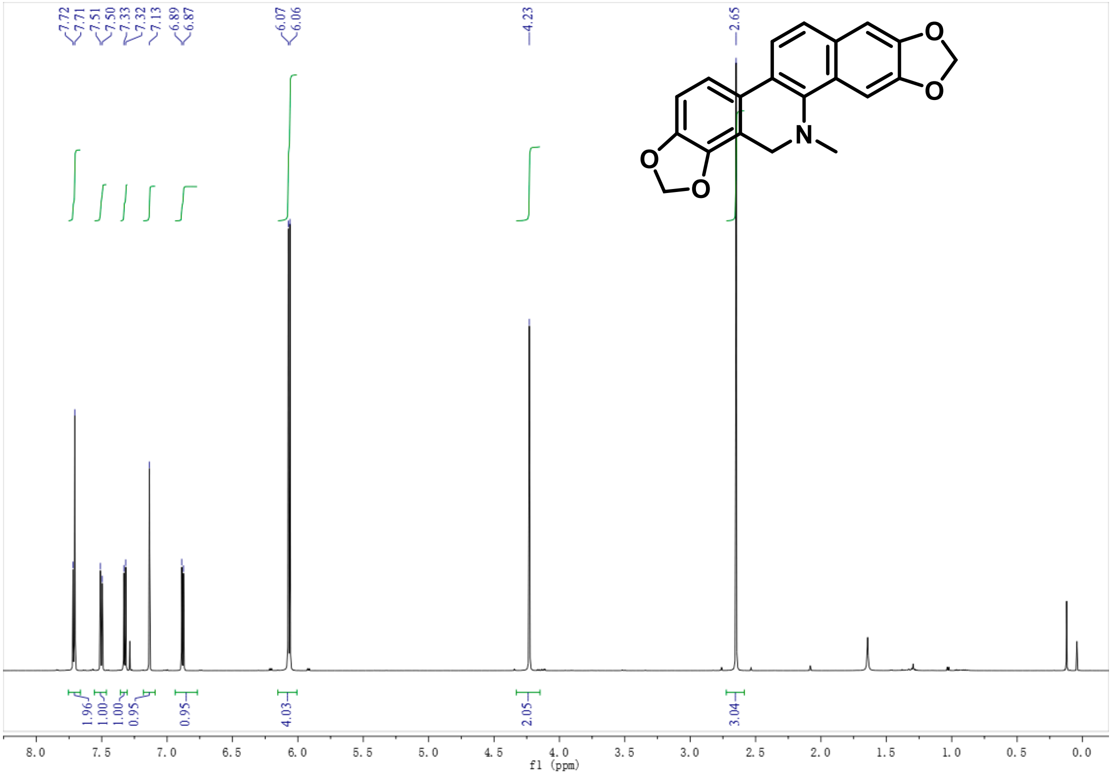

Figure S71. ^{1}H-NMR spectrum of **2b** (600 MHz, $CDCl_3$).

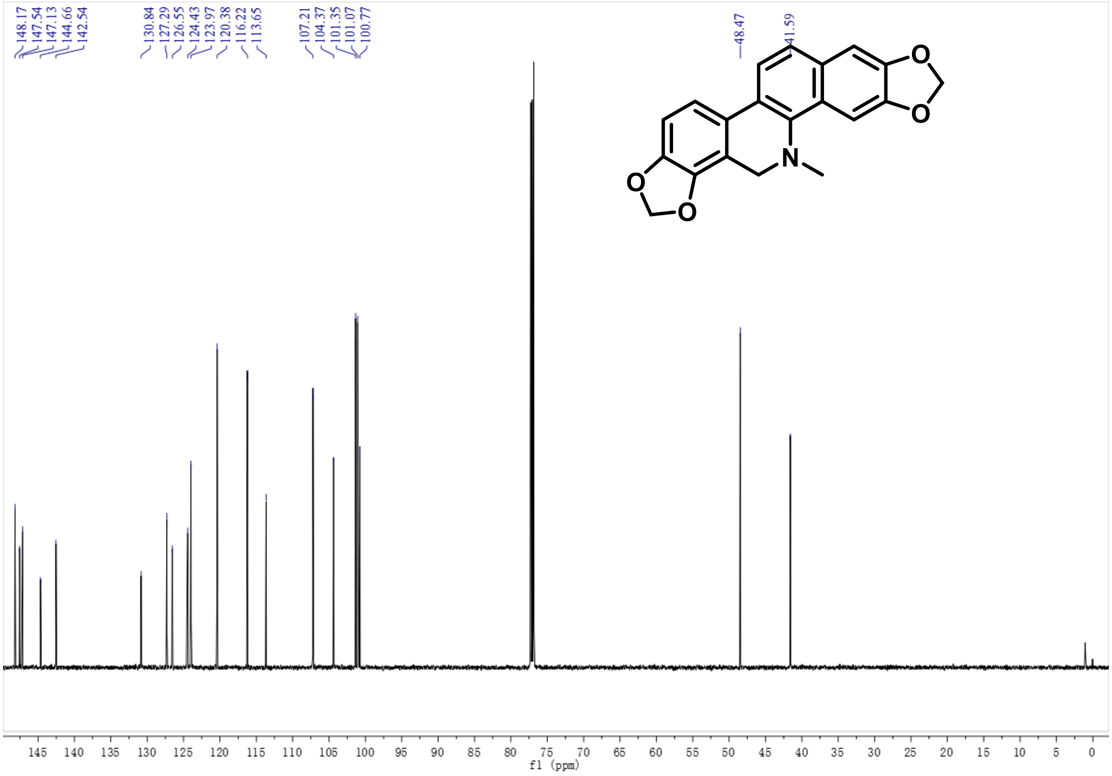

Figure S72. ^{13}C-NMR spectrum of **2b** (150 MHz, $CDCl_3$).

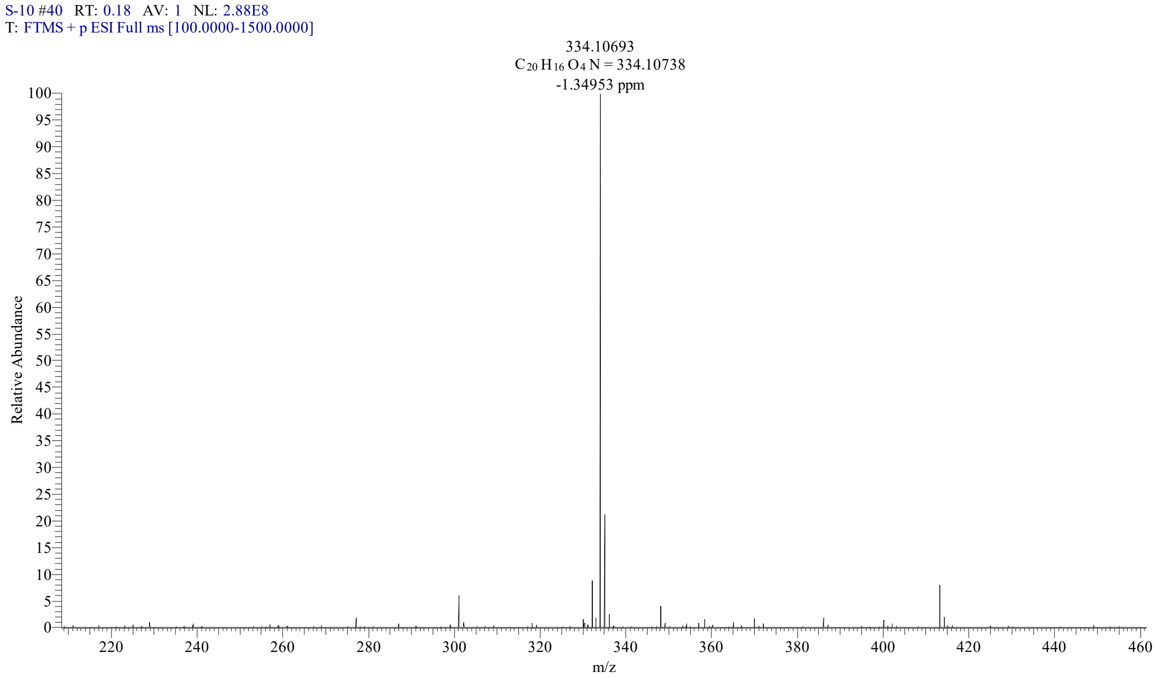

Figure S73. HR-ESI-MS spectrum of **2b.**

Compound **2c**

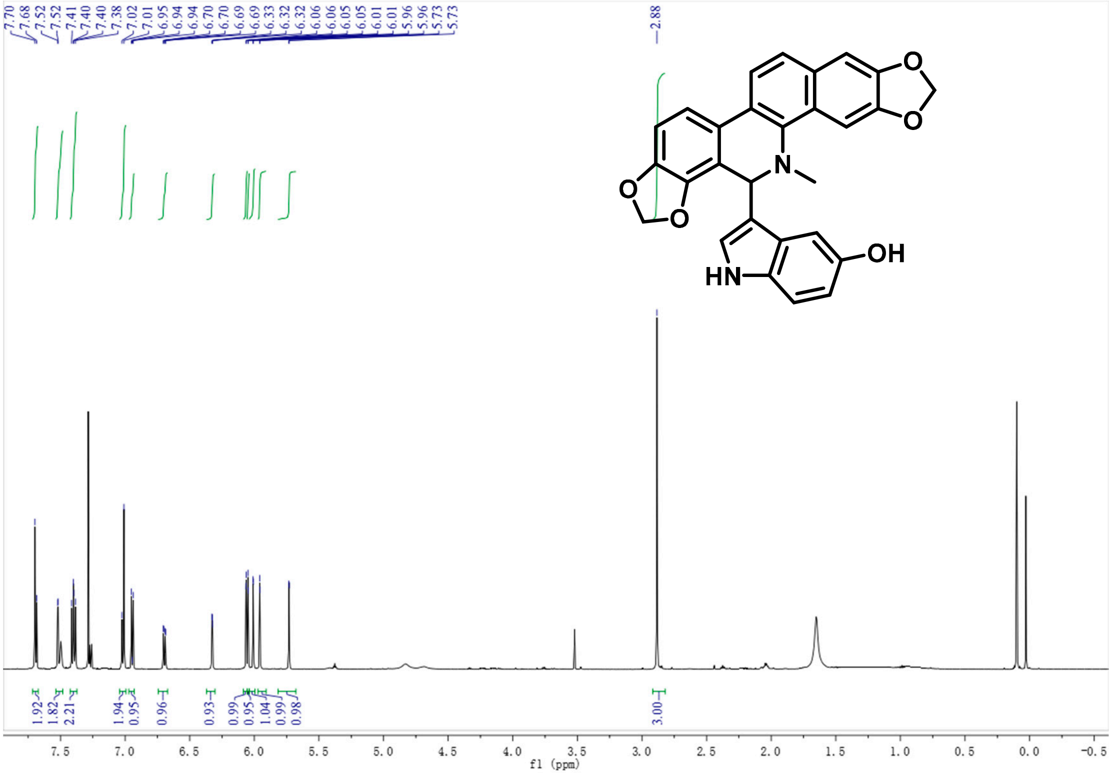

Figure S74. ^{1}H-NMR spectrum of **2c** (600 MHz, $CDCl_3$).

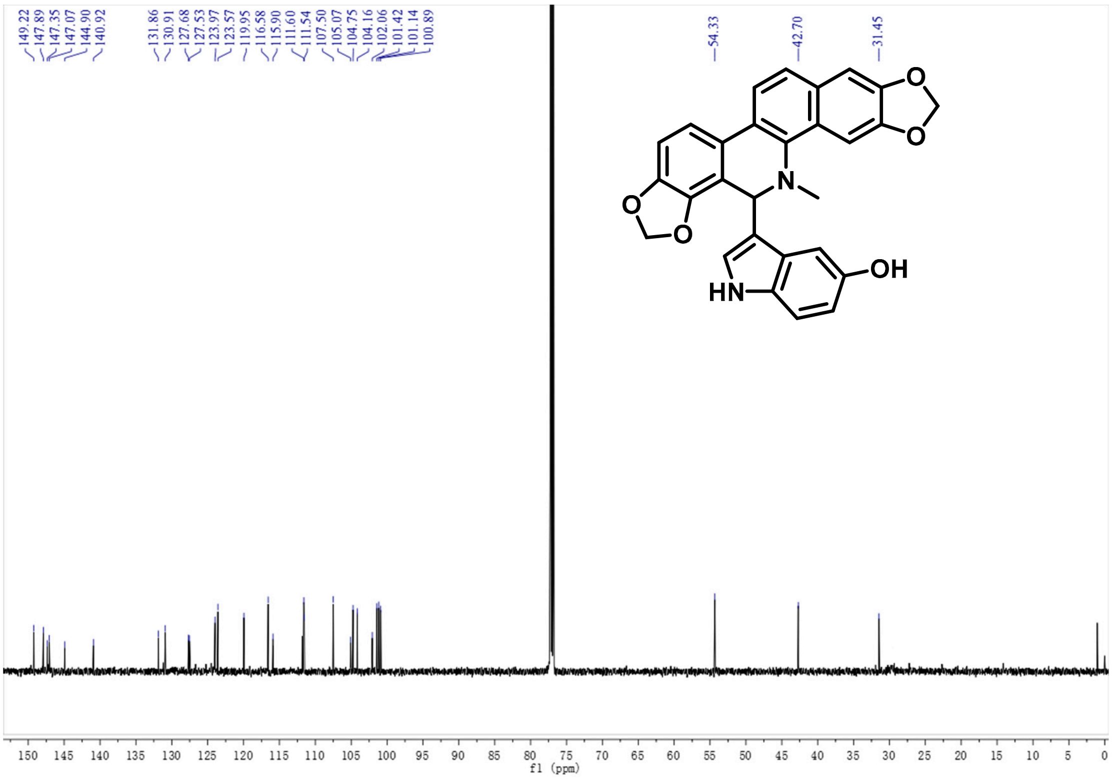

Figure S75. ^{13}C-NMR spectrum of **2c** (150 MHz, $CDCl_3$).

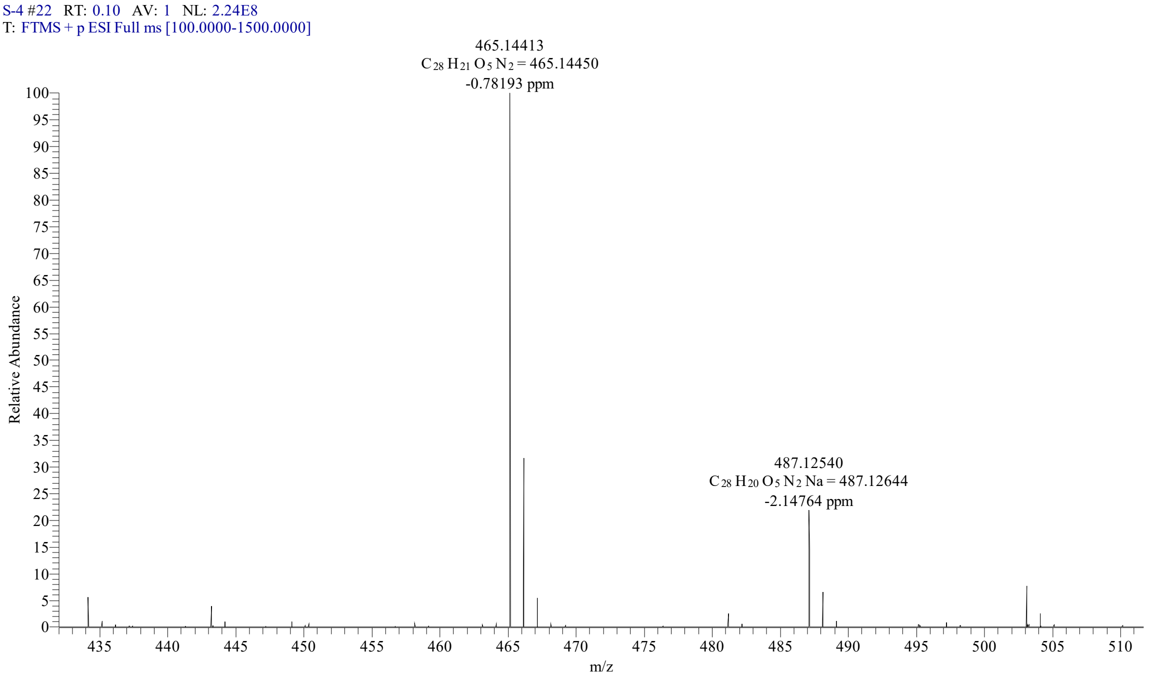

Figure S76. HR-ESI-MS spectrum of **2c.**

Compound **2d**

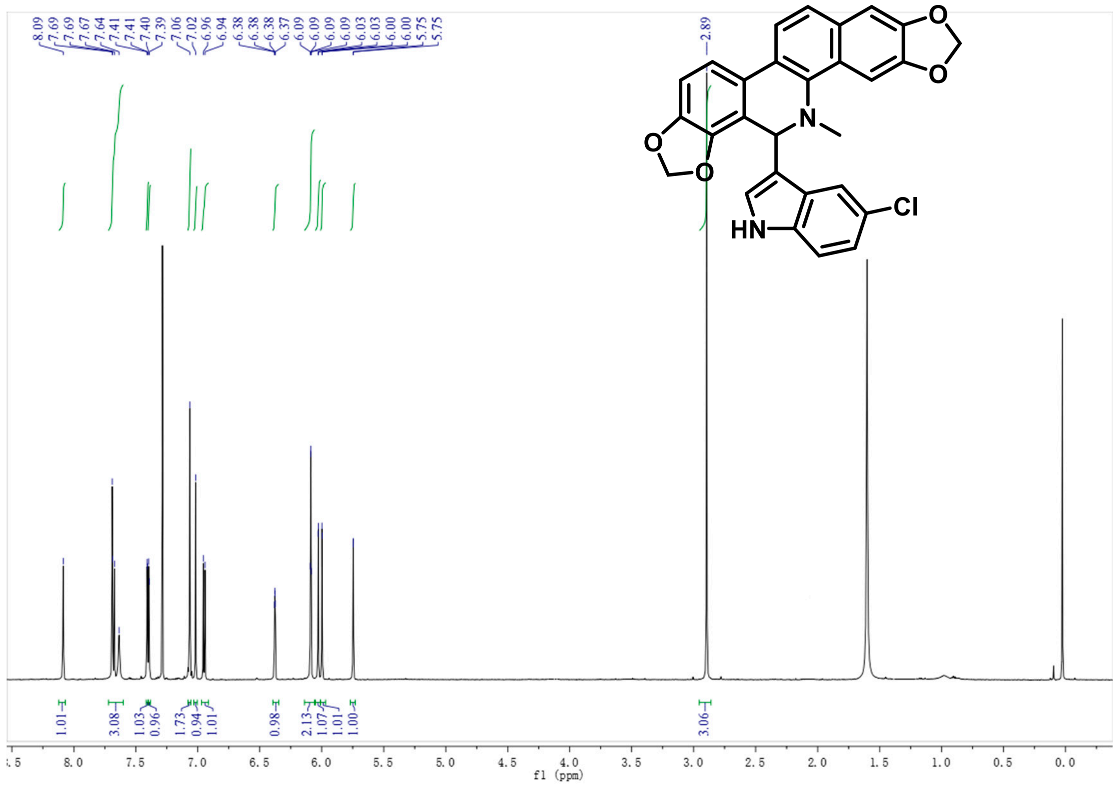

Figure S77. ^{1}H-NMR spectrum of **2d** (600 MHz, $CDCl_3$).

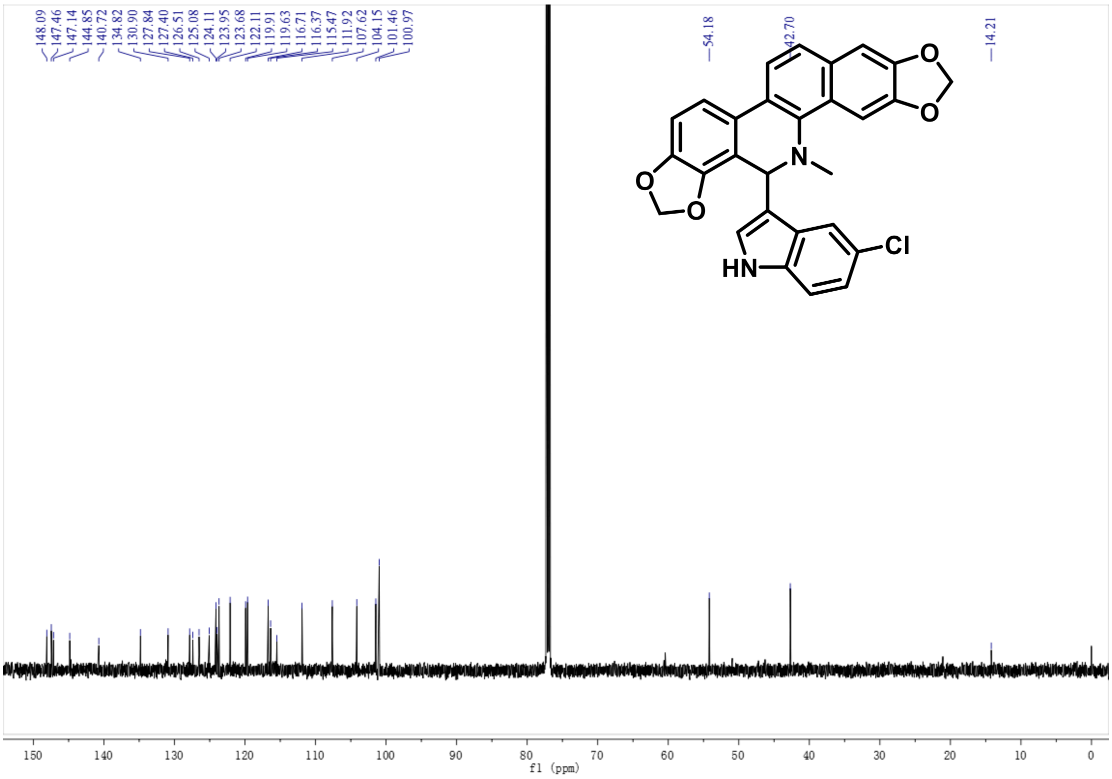

Figure S78. ^{13}C-NMR spectrum of **2d** (150 MHz, $CDCl_3$).

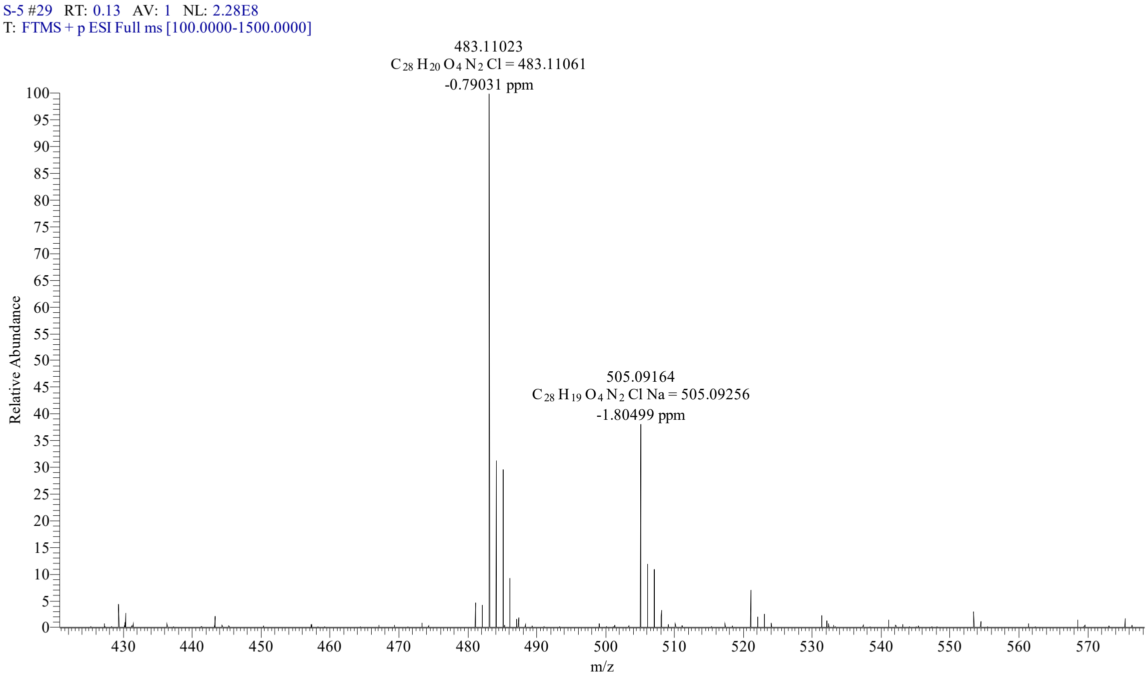

Figure S79. HR-ESI-MS spectrum of **2d.**

Compound **2e**

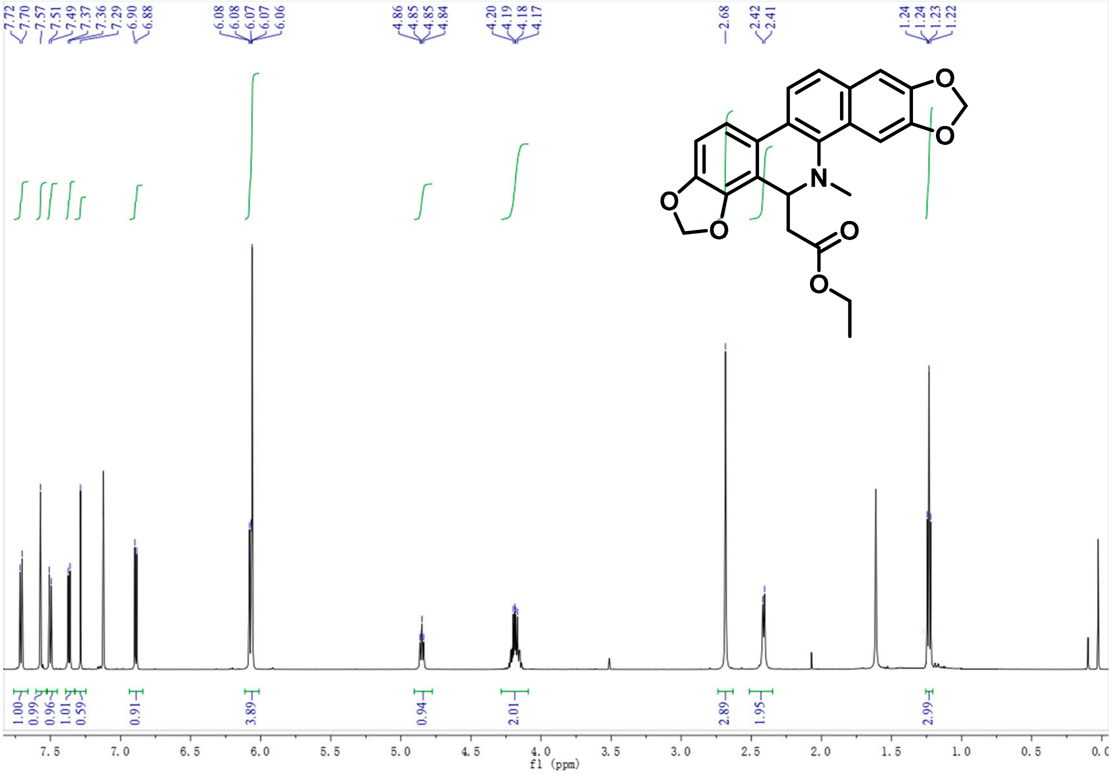

Figure S80. ^{1}H-NMR spectrum of **2e** (600 MHz, $CDCl_3$).

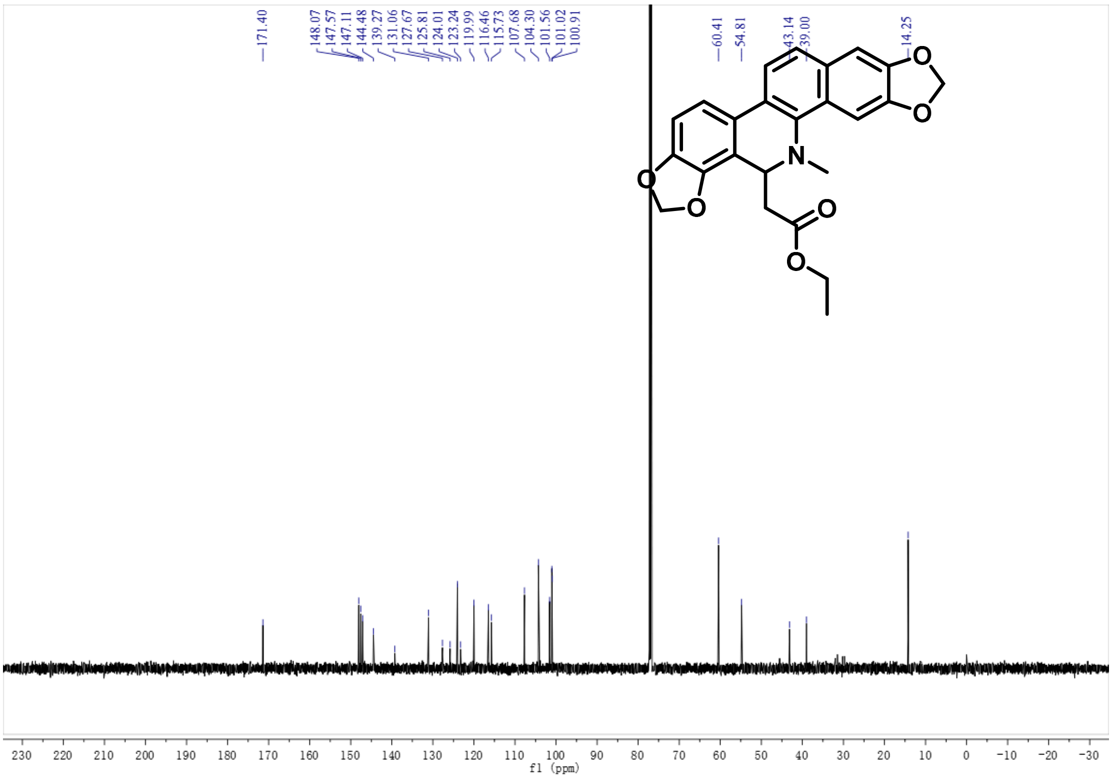

Figure S81. ^{13}C-NMR spectrum of **2e** (150 MHz, $CDCl_3$).

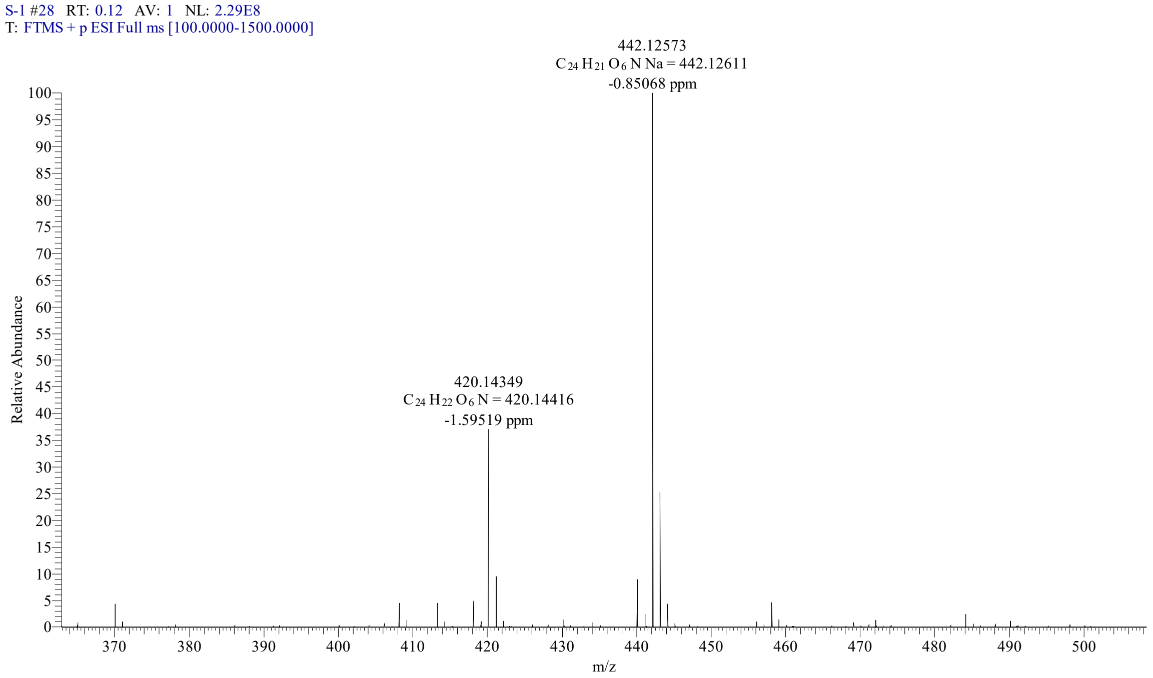

Figure S82. HR-ESI-MS spectrum of **2e.**

Compound **2f**

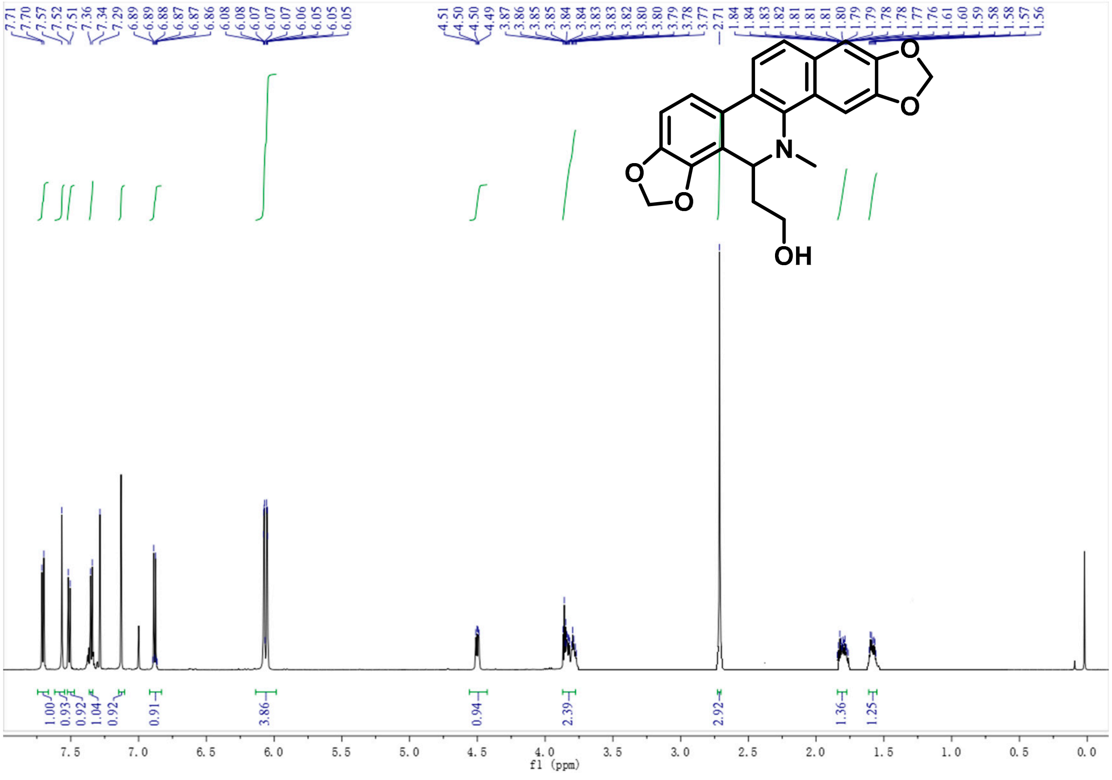

Figure S83. ^{1}H-NMR spectrum of **2f** (600 MHz, $CDCl_3$).

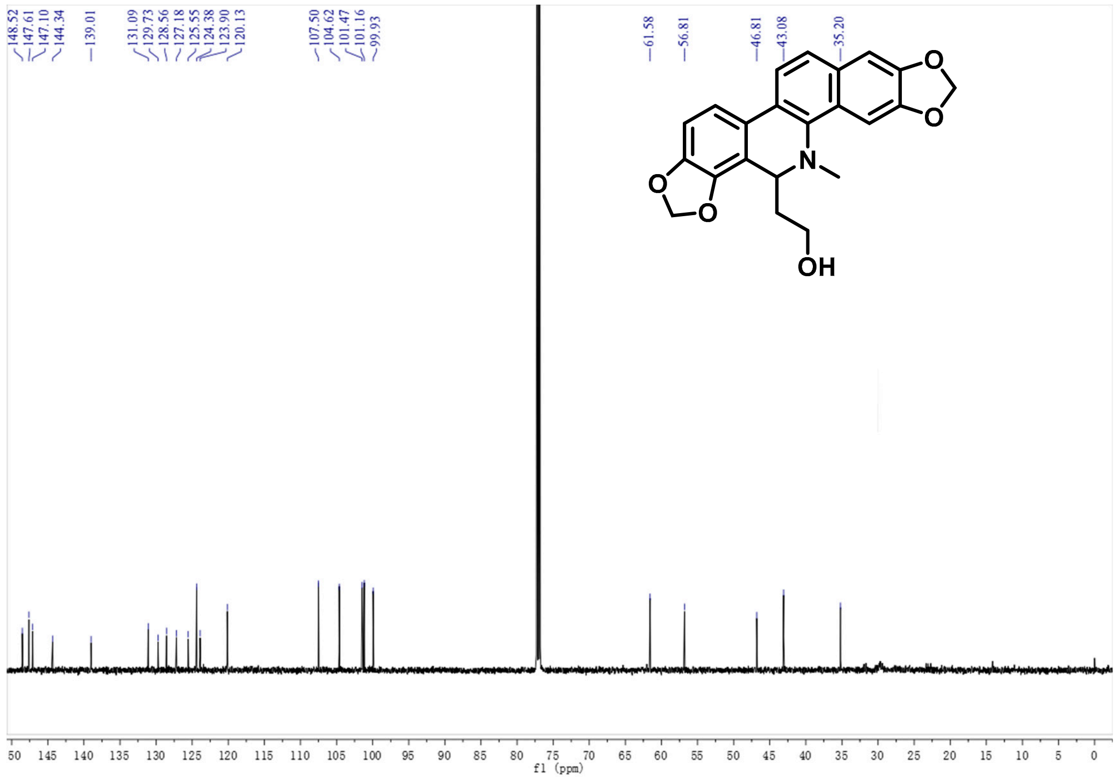

Figure S84. ^{13}C-NMR spectrum of **2f** (150 MHz, $CDCl_3$).

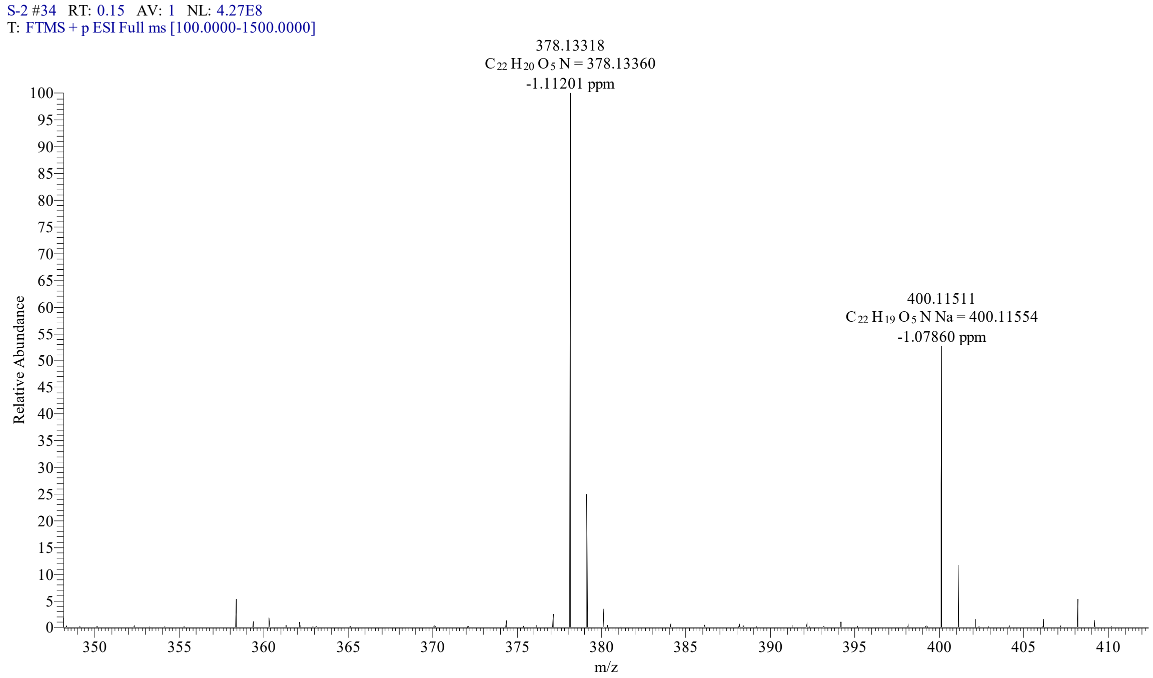

Figure S85. HR-ESI-MS spectrum of **2f.**

Compound **2g**

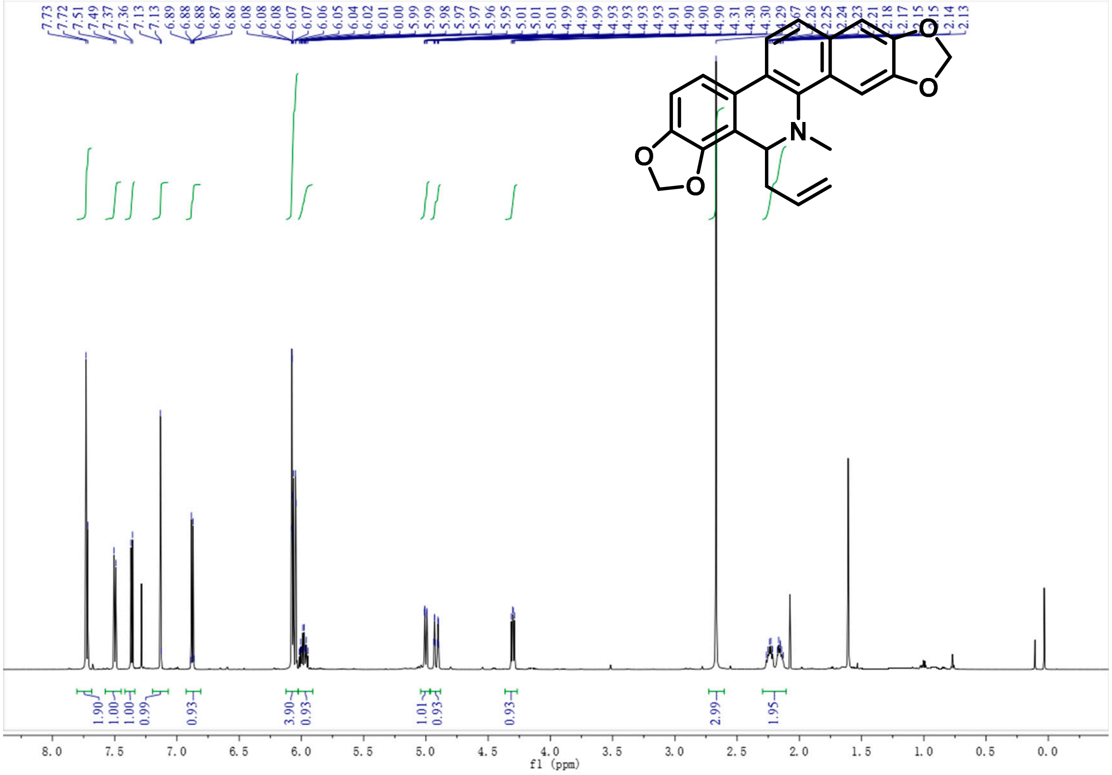

Figure S86. ¹H-NMR spectrum of **2g** (600 MHz, $CDCl_3$).

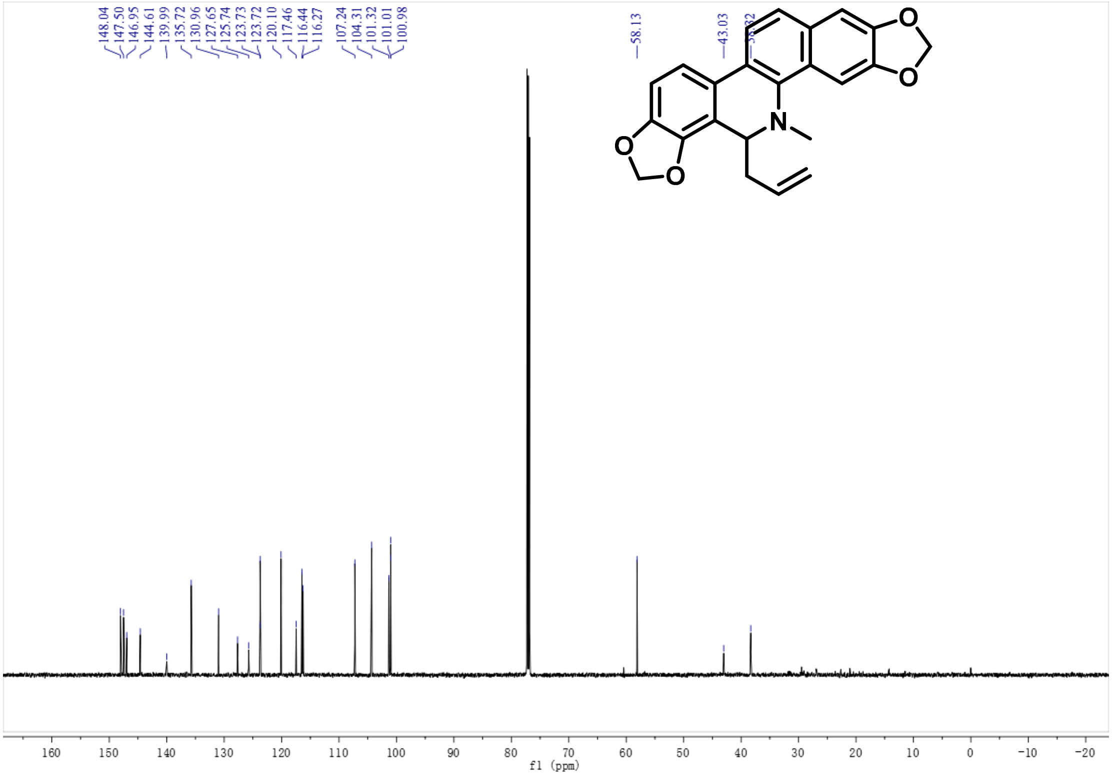

Figure S87. ¹³C-NMR spectrum of **2g** (150 MHz, $CDCl_3$).

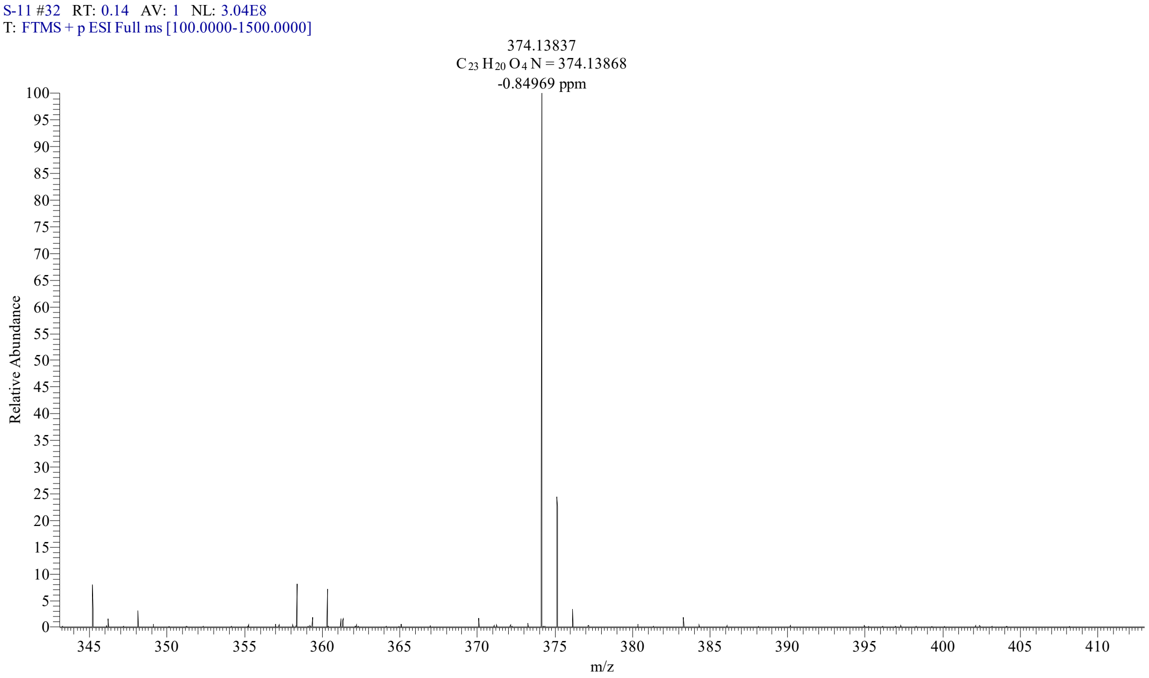

Figure S88. HR-ESI-MS spectrum of **2g**

Compound **2h**

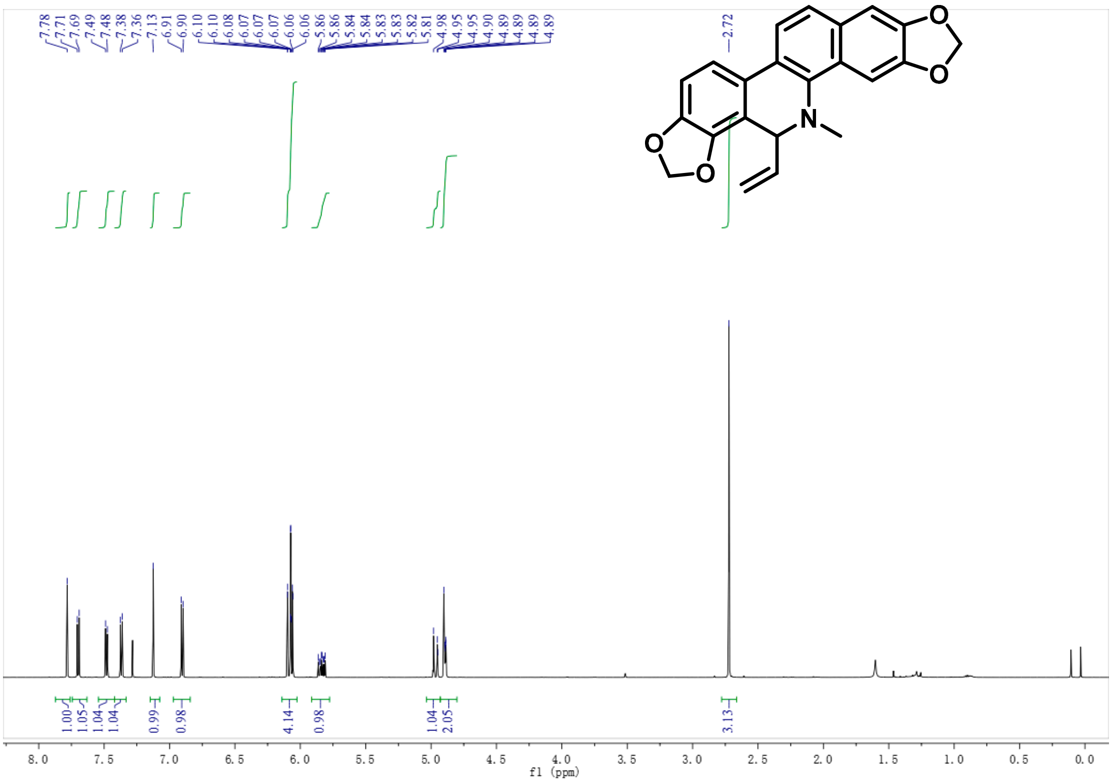

Figure S89. ^{1}H-NMR spectrum of **2h** (600 MHz, $CDCl_3$).

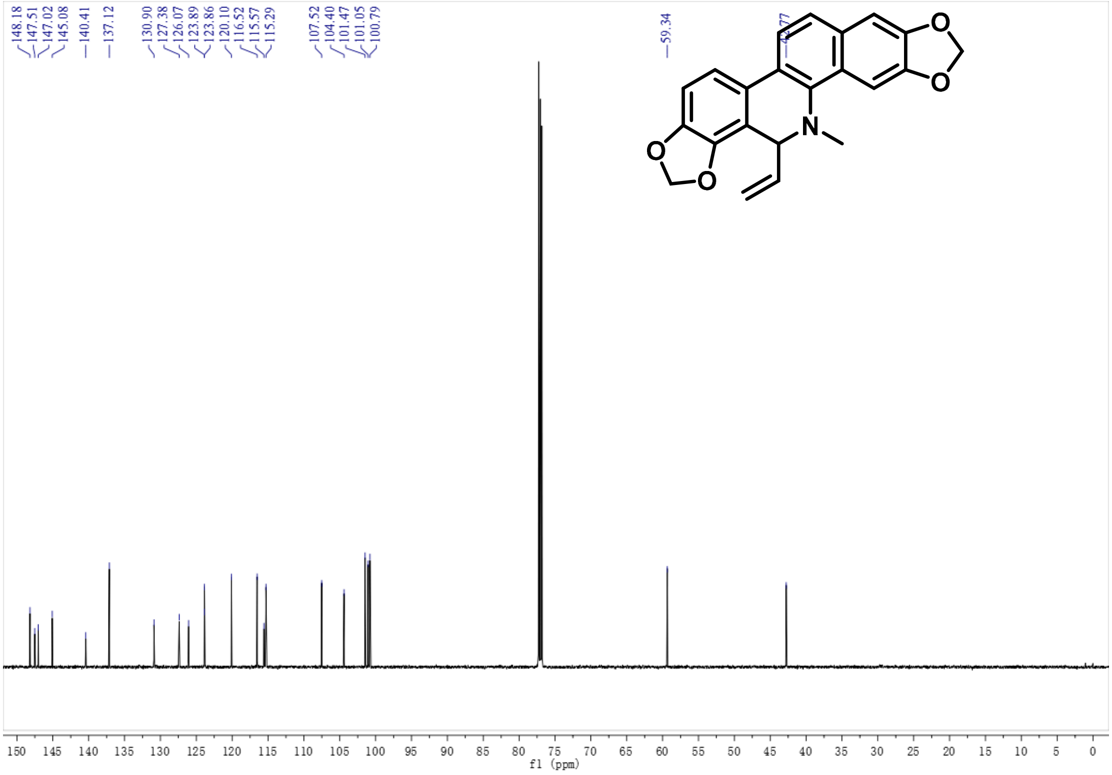

Figure S90. ^{13}C-NMR spectrum of **2h** (150 MHz, $CDCl_3$).

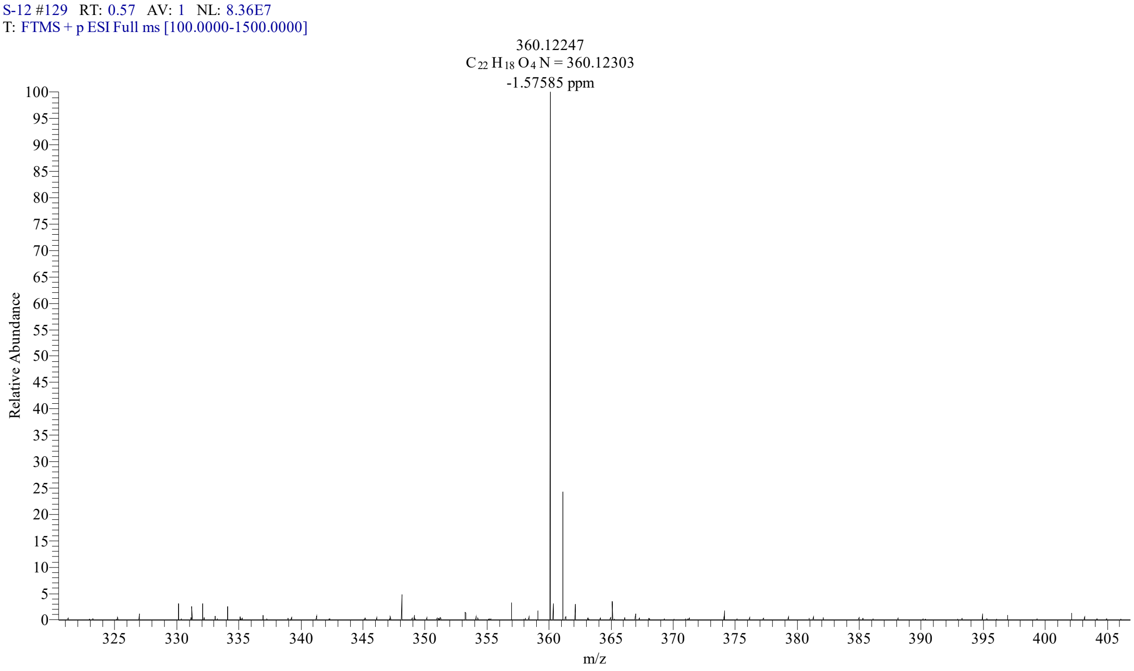

Figure S91. HR-ESI-MS spectrum of **2h.**

Compound **2i**

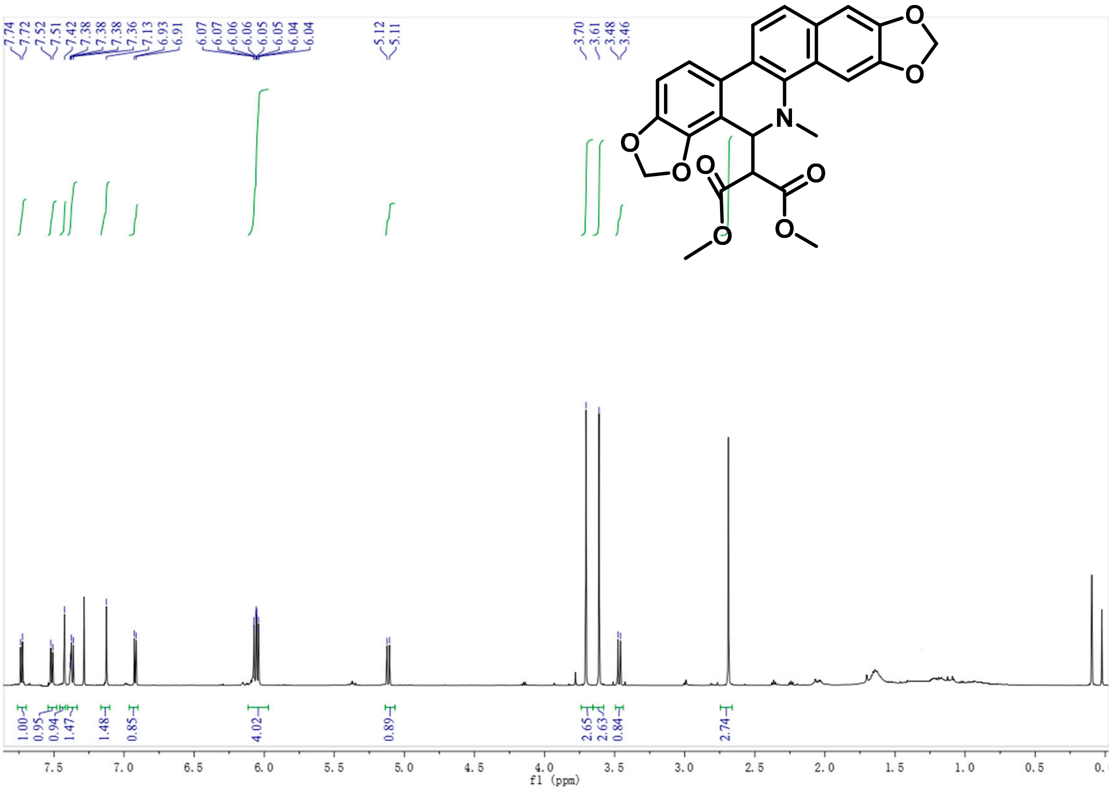

Figure S92. ^{1}H-NMR spectrum of **2i** (600 MHz, $CDCl_3$).

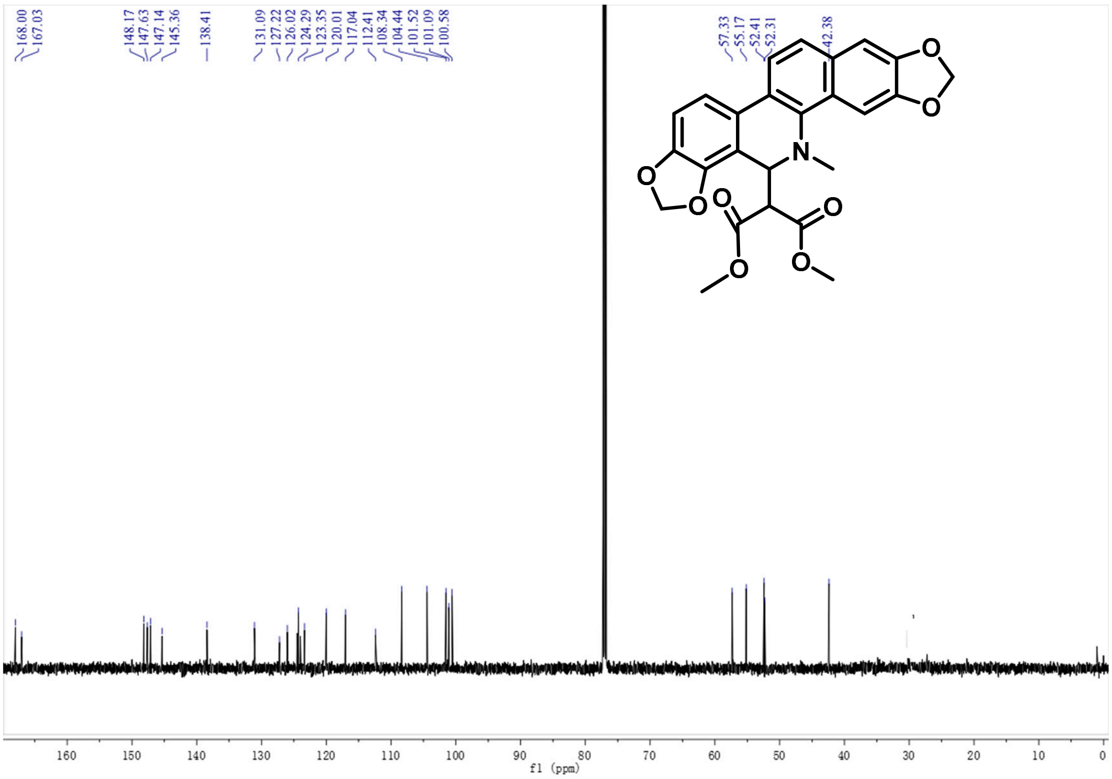

Figure S93. ^{13}C-NMR spectrum of **2i** (150 MHz, $CDCl_3$).

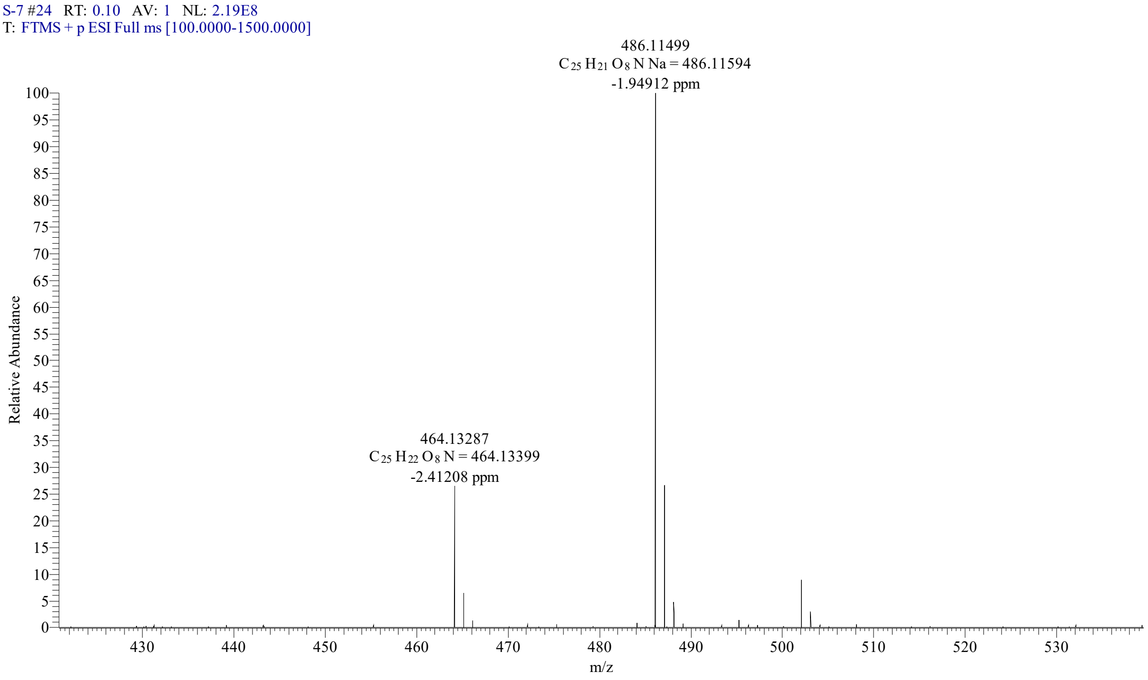

Figure S94. HR-ESI-MS spectrum of **2i.**

Compound **2j**

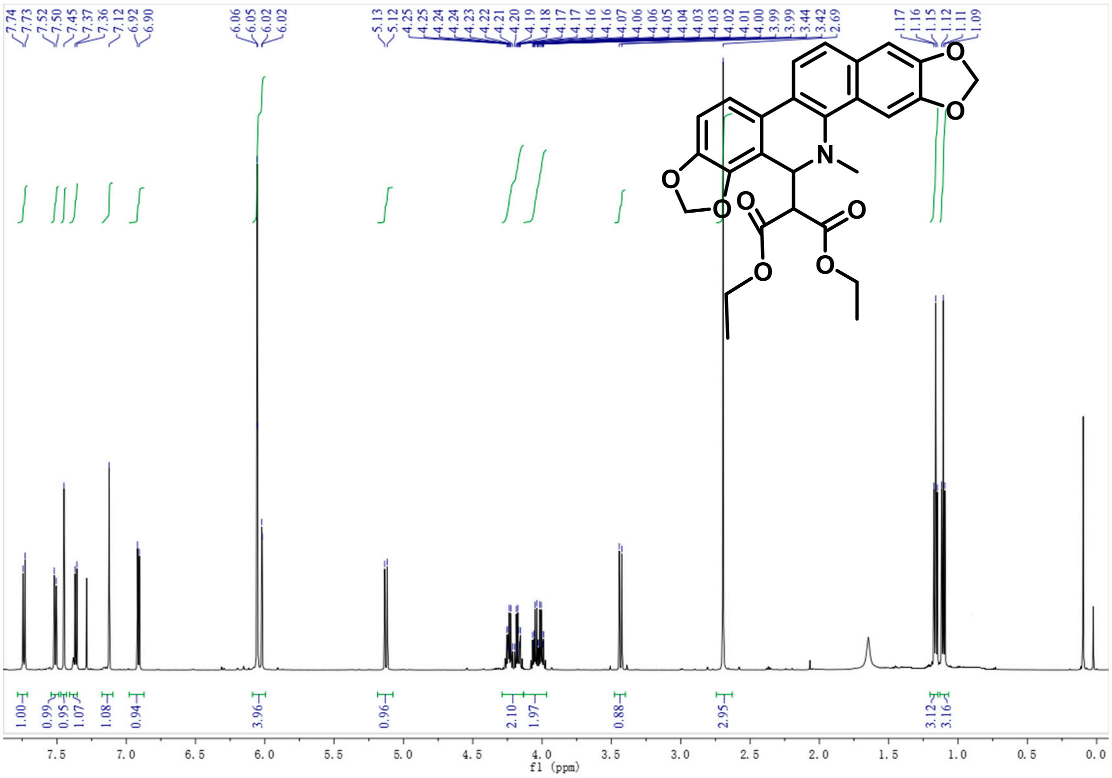

Figure S95. ^{1}H-NMR spectrum of **2j** (600 MHz, $CDCl_3$).

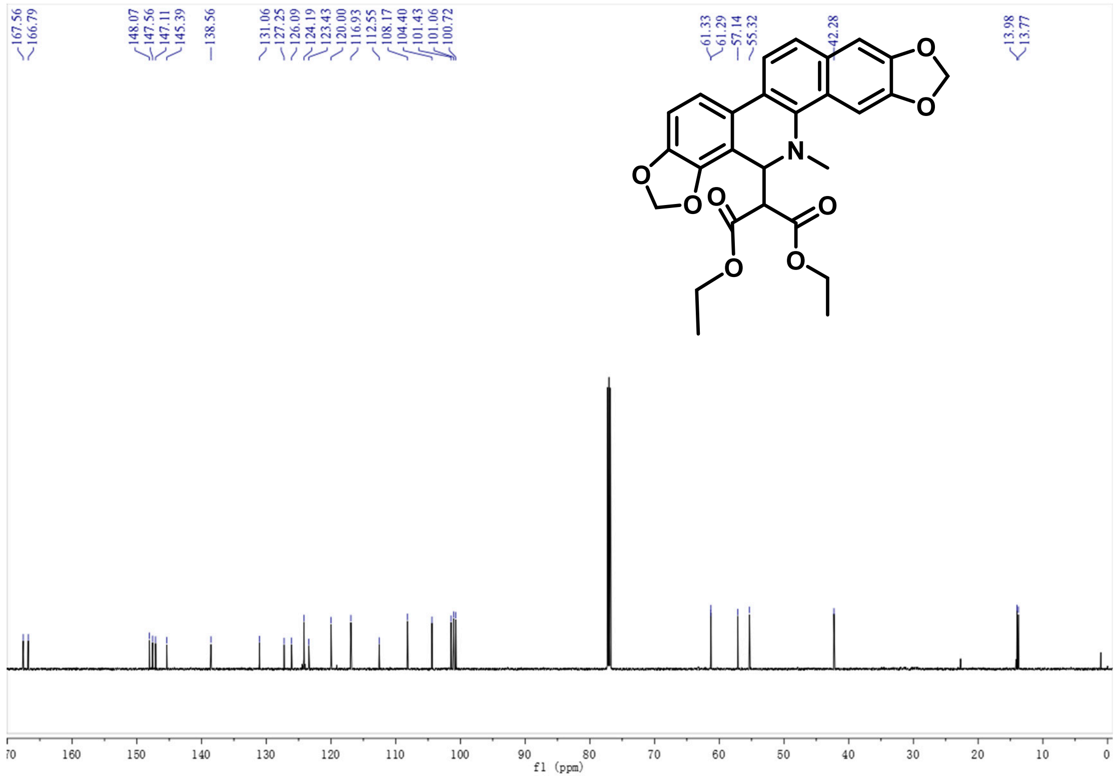

Figure S96. ^{13}C-NMR spectrum of **2j** (150 MHz, $CDCl_3$).

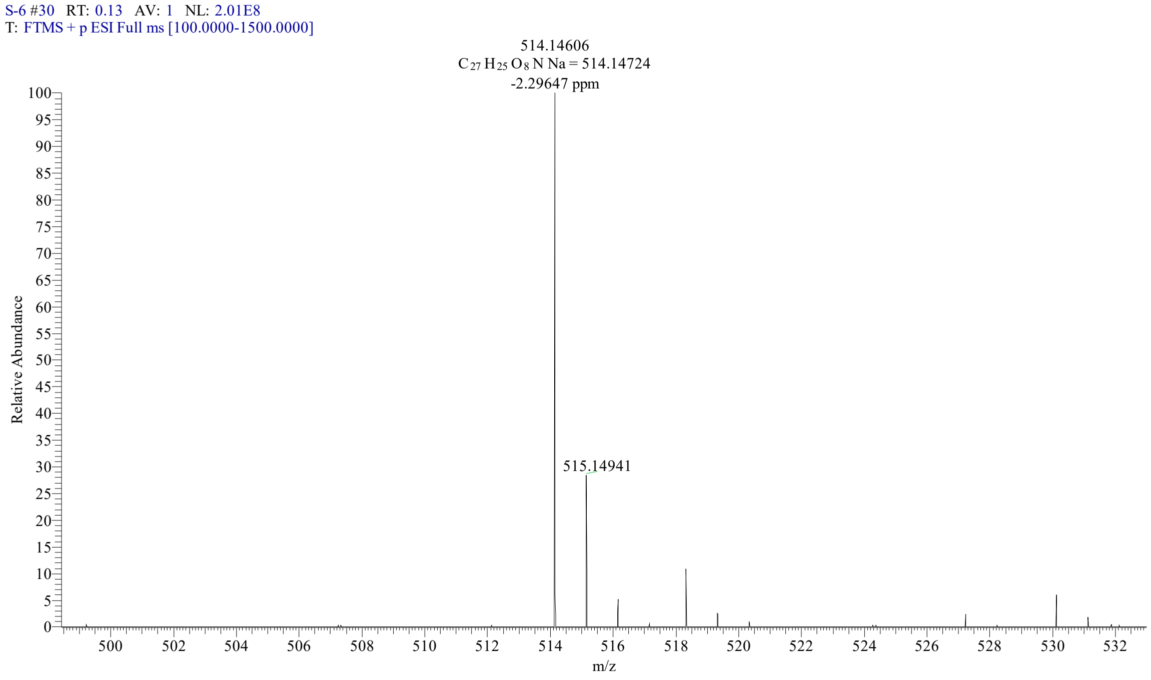

Figure S97. HR-ESI-MS spectrum of **2j.**

Compound **2k**

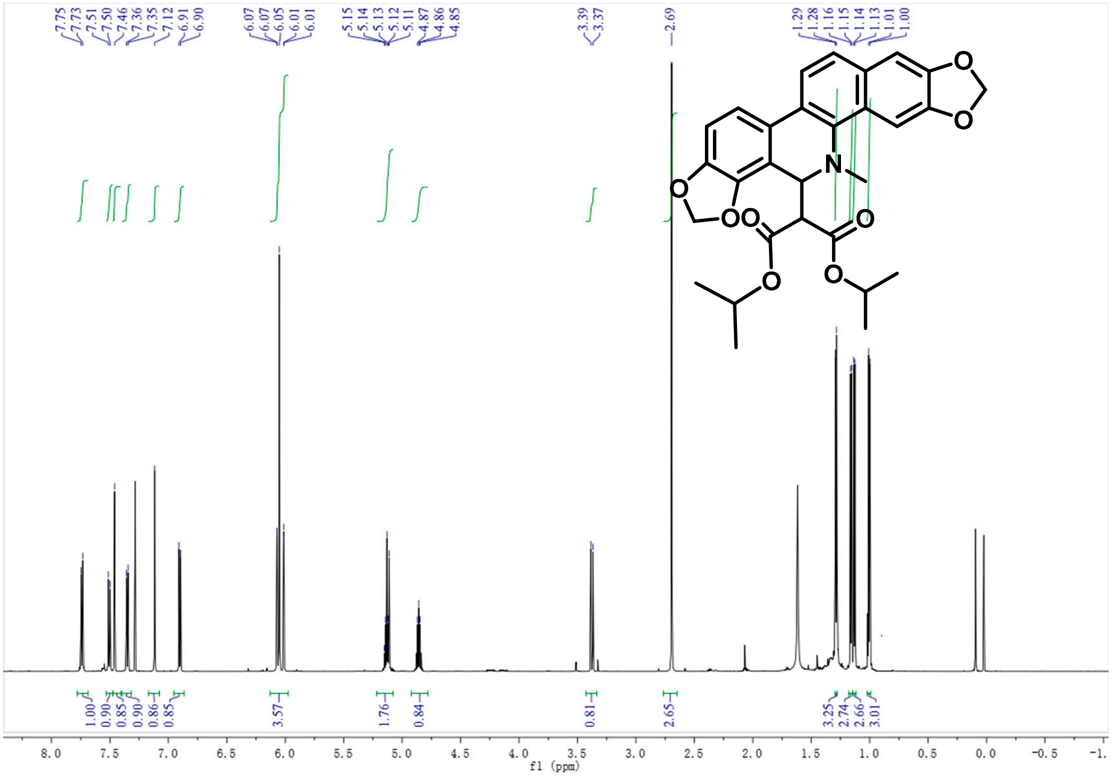

Figure S98. ^{1}H-NMR spectrum of **2k** (600 MHz, $CDCl_3$).

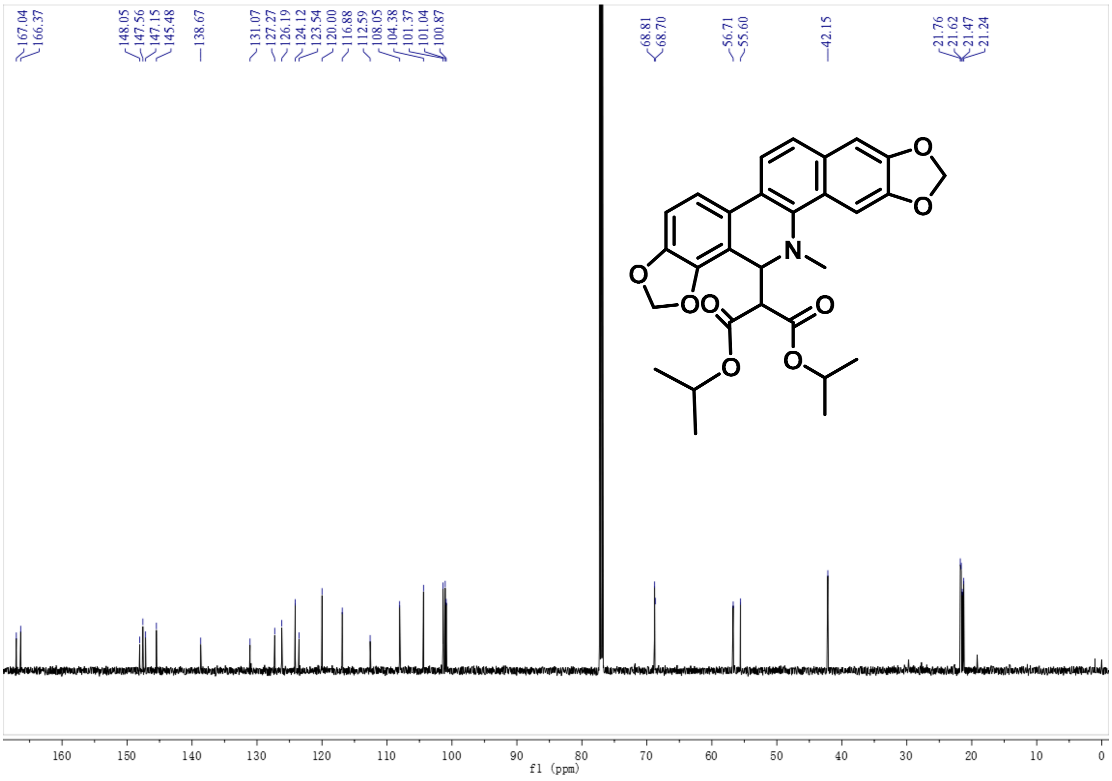

Figure S99. ^{13}C-NMR spectrum of **2k** (150 MHz, $CDCl_3$).

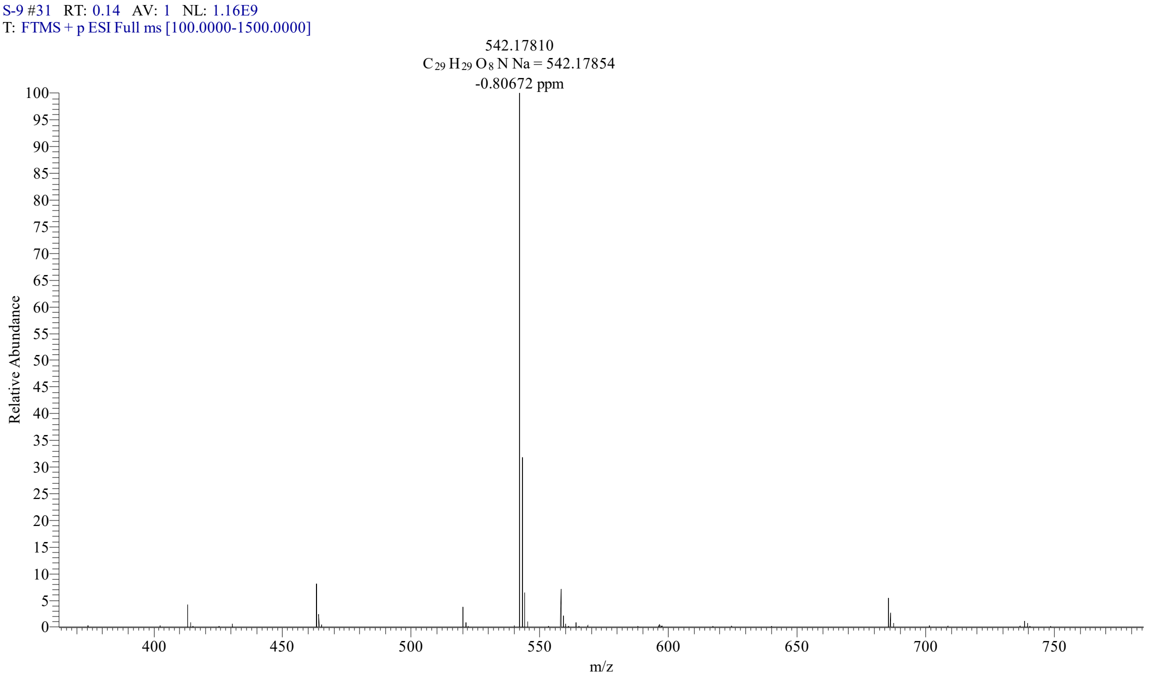

Figure S100. HR-ESI-MS spectrum of **2k.**

Compound **2l**

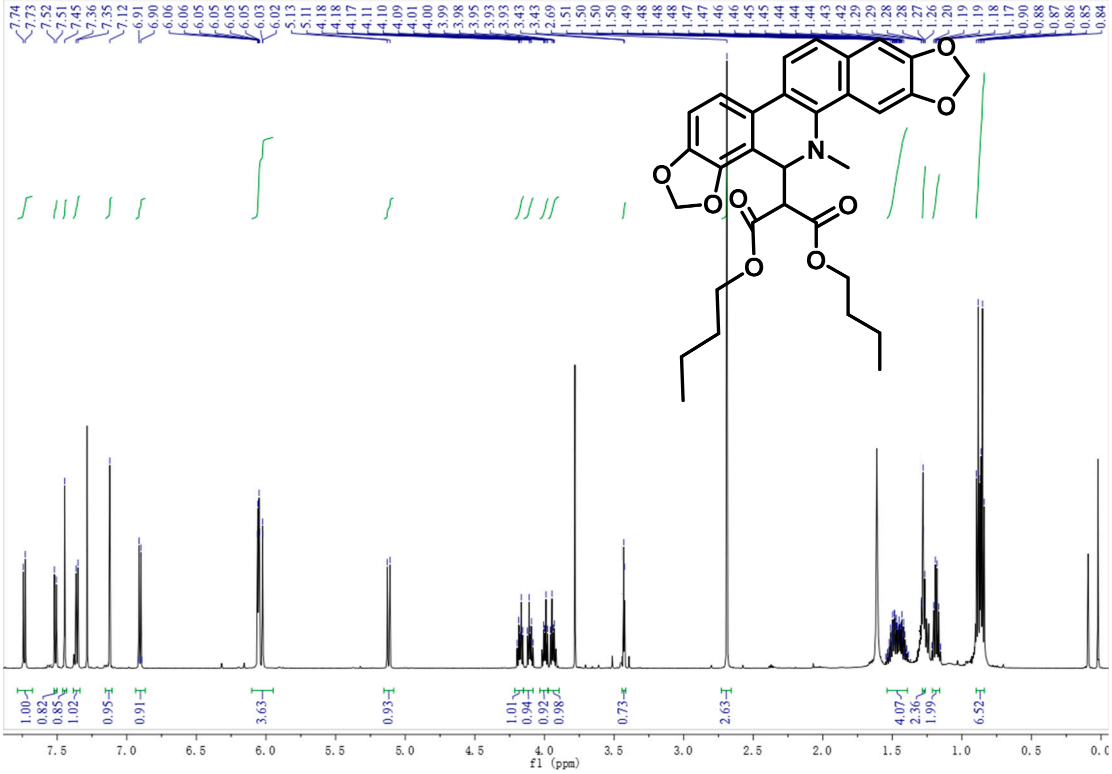

Figure S101. ^{1}H-NMR spectrum of **2l** (600 MHz, $CDCl_3$).

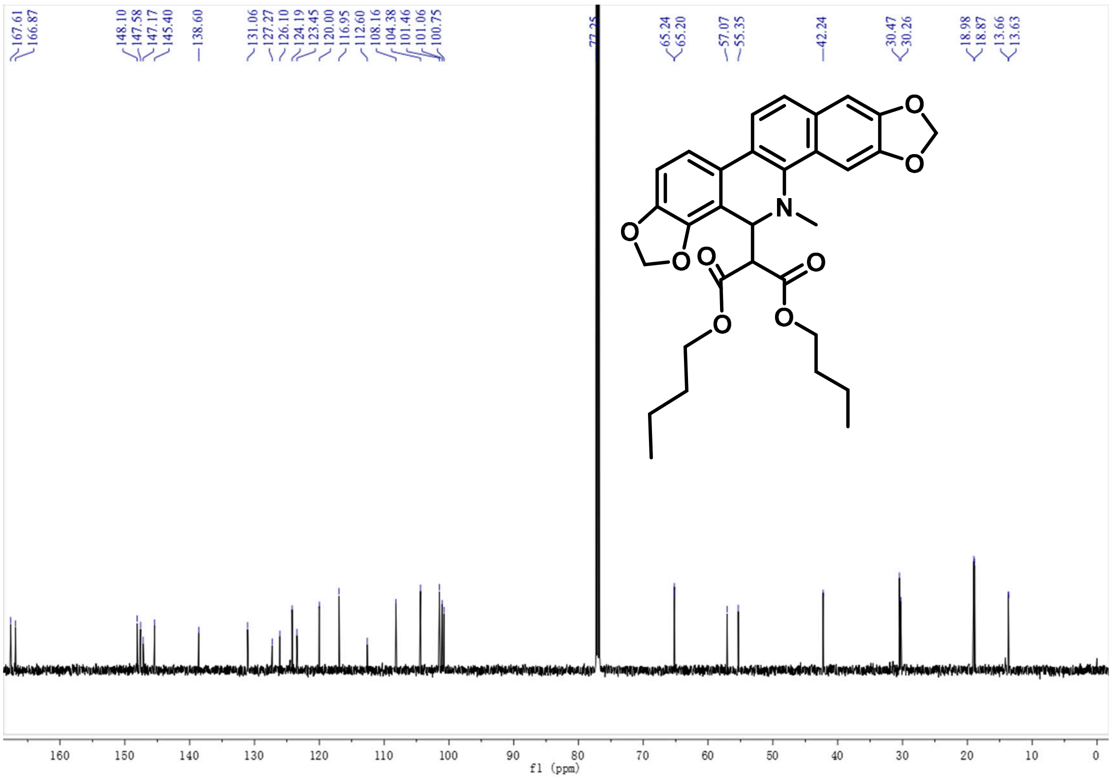

Figure S102. ^{13}C-NMR spectrum of **2l** (150 MHz, $CDCl_3$).

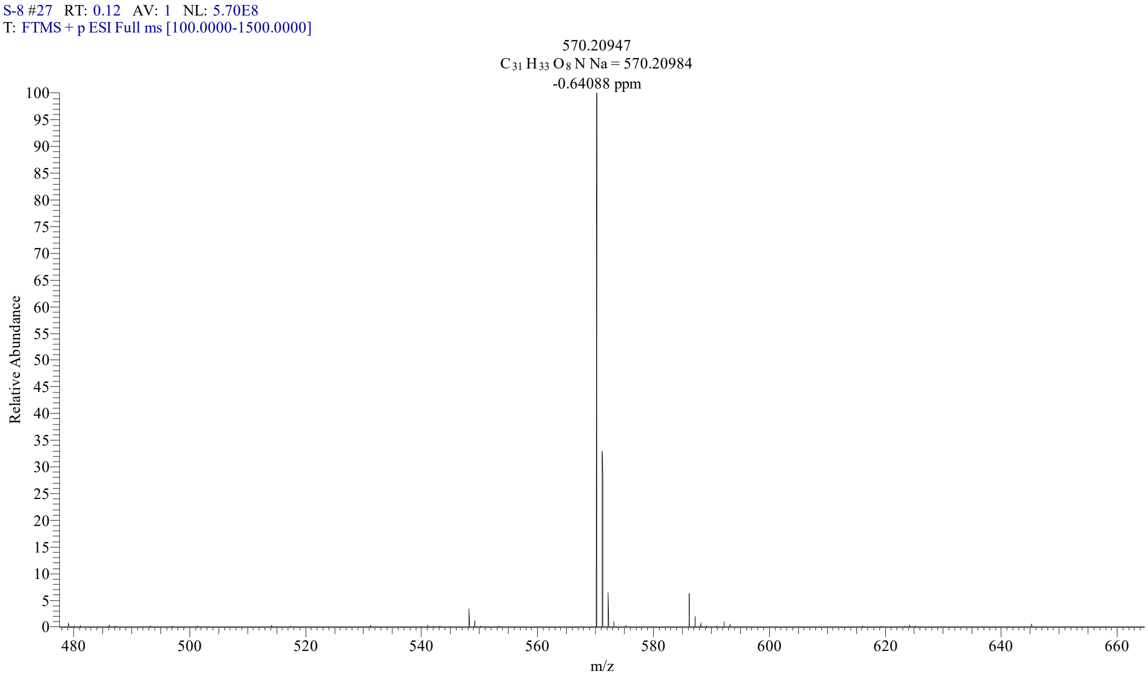

Figure S103. HR-ESI-MS spectrum of **2l.**

The HPLC chromatograms of compounds 1, 2, 1a–1u, and 2a–2l

HPLC chromatogram conditions

The chromatographic column was obtained from Thermo Science (Part No. 25905-254630), and the chromatograph was run with a flow rate of 1.0 mL/min and a column temperature maintained at 30 °C. The mobile phase system was acetonitrile (A) and 0.1% phosphoric acid aqueous solution (B); injection volume and detection wavelength are presented in Table 1.

Table S1. The mobile phase system, injection volume, and detection wavelength of compounds.

<table>
<tr><th>Compd.</th><th>A:B (%)</th><th>Injection Volume (μL)</th><th>Detection Wavelength (nm)</th></tr>
<tr><td>1, 2, 1a, 1c, 1e–1g, 1l, 1n, 1t, 2b, 2d, 2f–2h, 2j–2l</td><td rowspan="2">70:30</td><td>10.0</td><td>269</td></tr>
<tr><td>1b, 1d, 1h–1k, 1m, 1q–1r, 1u, 2a, 2c, 2e, 2i</td><td>0.8</td><td>269</td></tr>
<tr><td>1o, 1p</td><td>60:40</td><td>0.8</td><td>290</td></tr>
<tr><td>1s</td><td>90:10</td><td>0.8</td><td>285</td></tr>
</table>

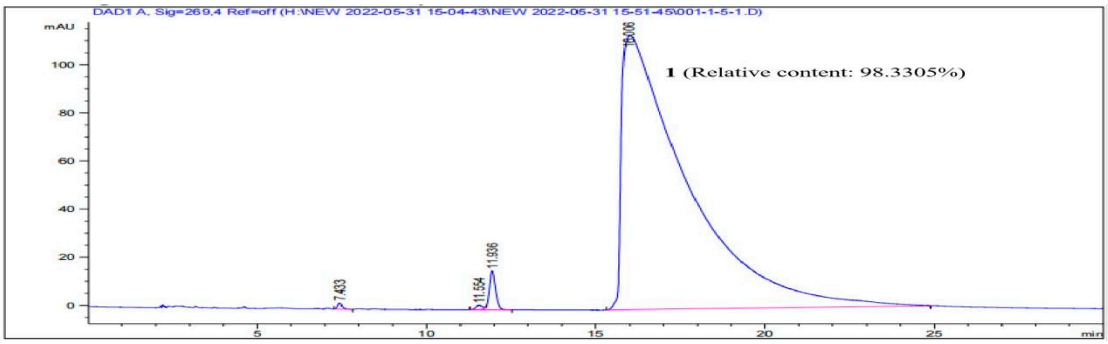

Figure S104. HPLC chromatogram of **1**.

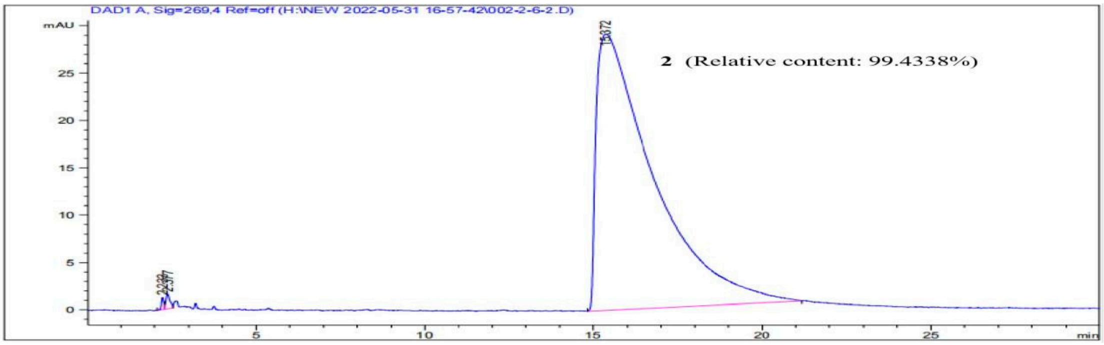

Figure S105. HPLC chromatogram of **2**.

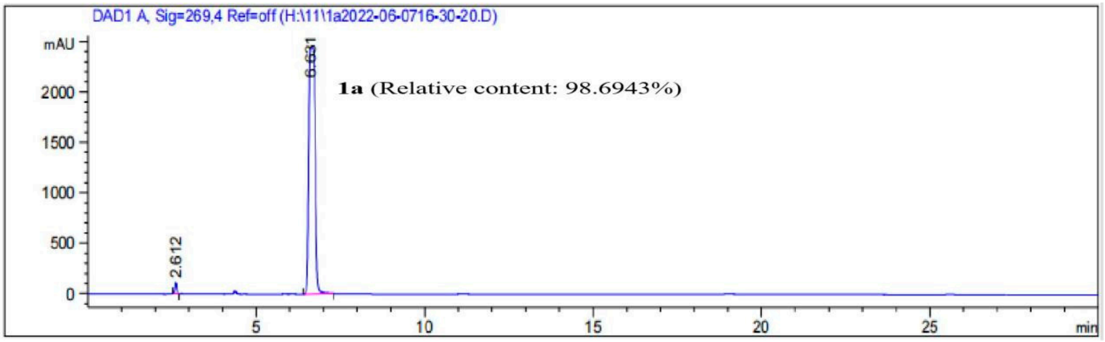

Figure S106. HPLC chromatogram of **1a.**

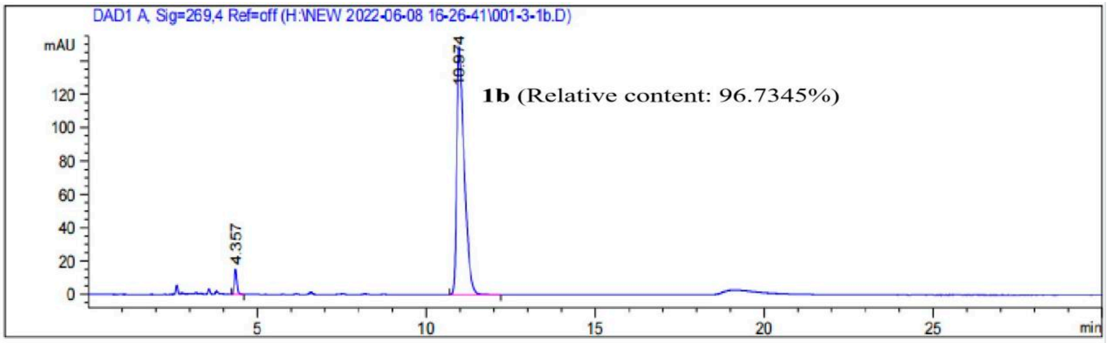

Figure S107. HPLC chromatogram of **1b.**

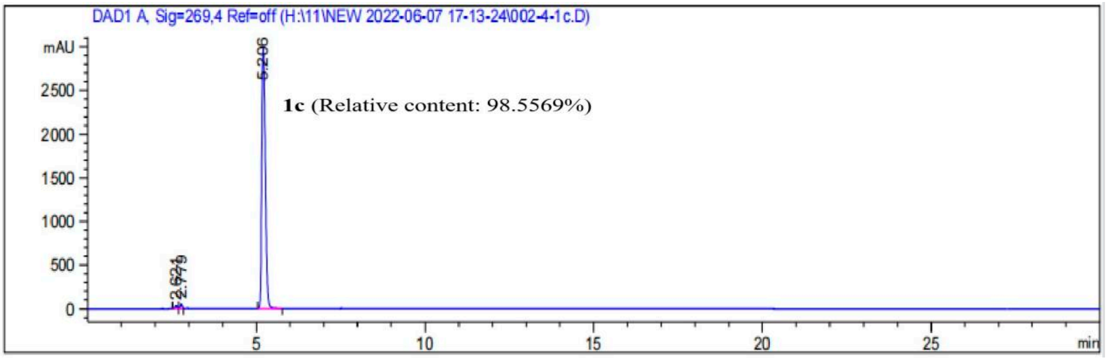

Figure S108. HPLC chromatogram of **1c.**

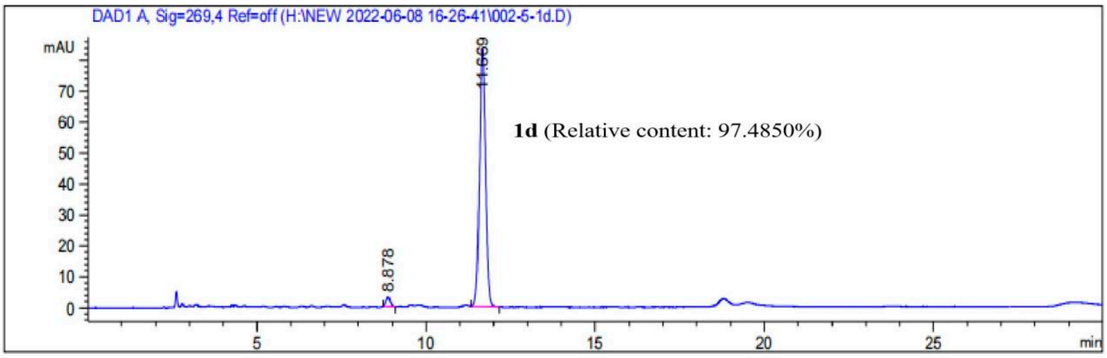

Figure S109. HPLC chromatogram of **1d.**

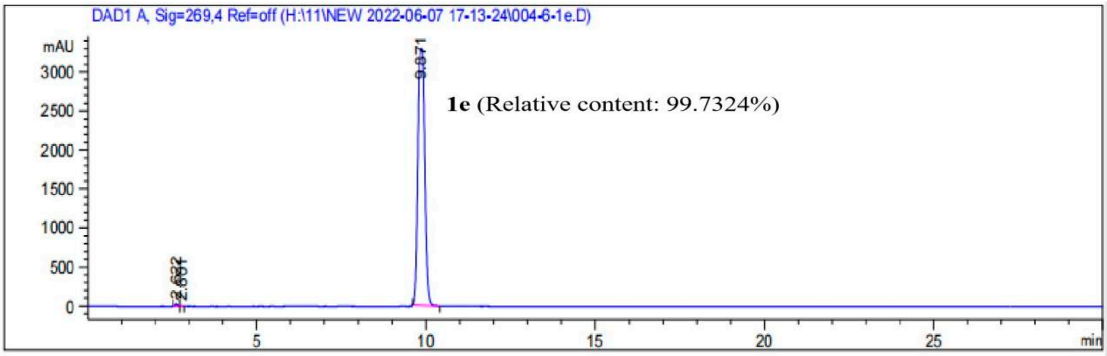

Figure S110. HPLC chromatogram of **1e.**

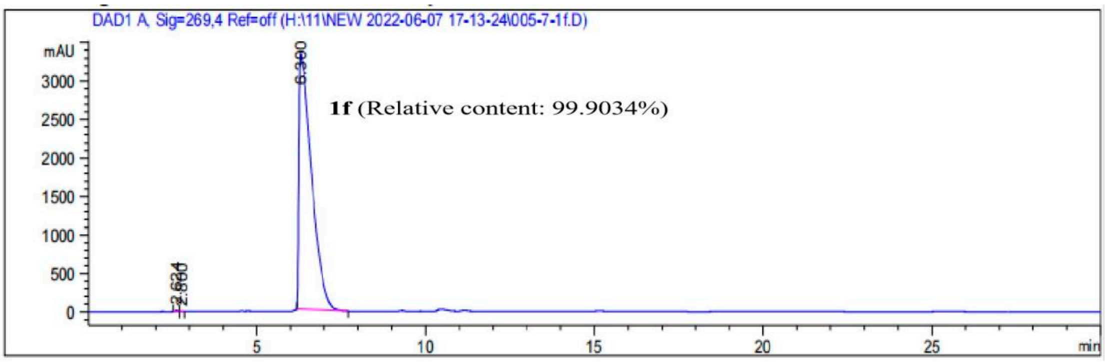

Figure S111. HPLC chromatogram of **1f.**

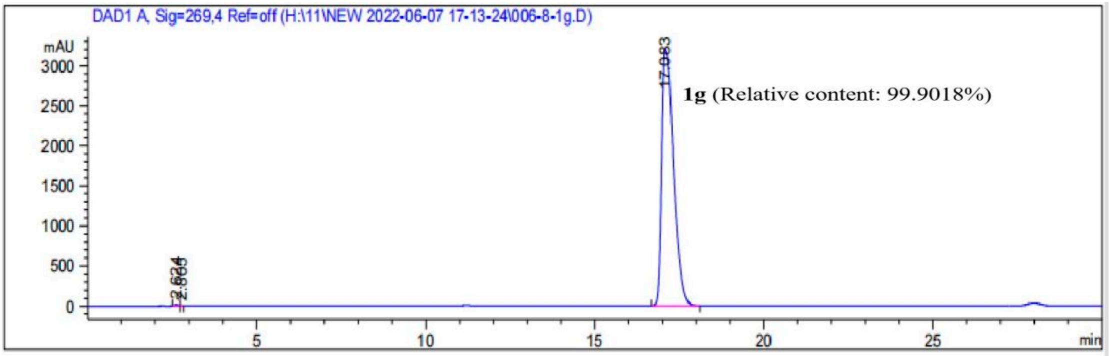

Figure S112. HPLC chromatogram of **1g.**

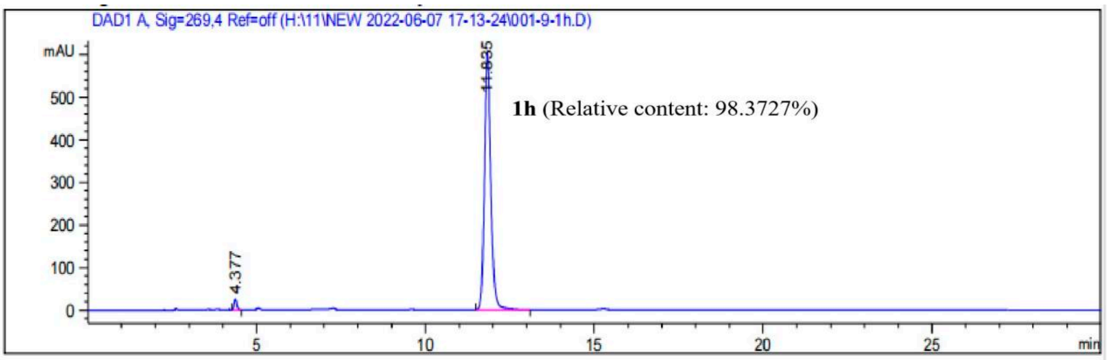

Figure S113. HPLC chromatogram of **1h.**

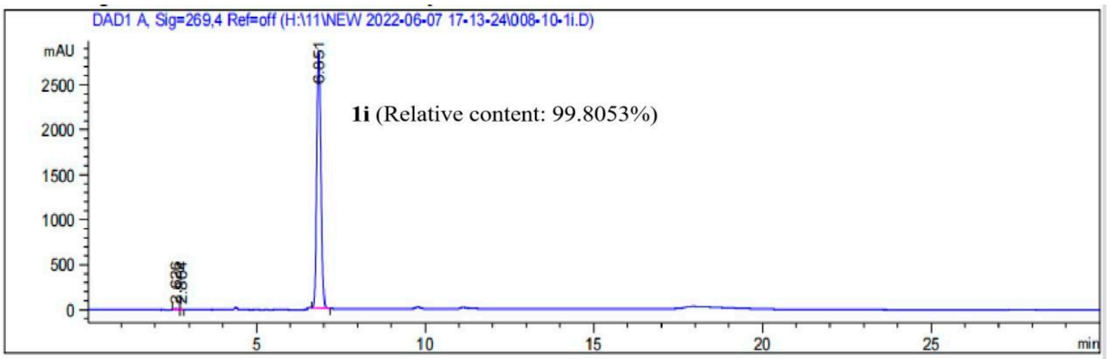

Figure S114. HPLC chromatogram of **1i.**

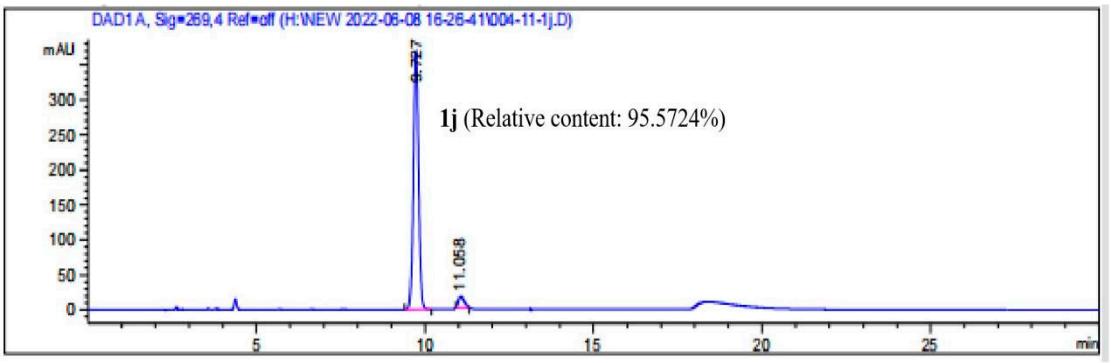

Figure S115. HPLC chromatogram of **1j.**

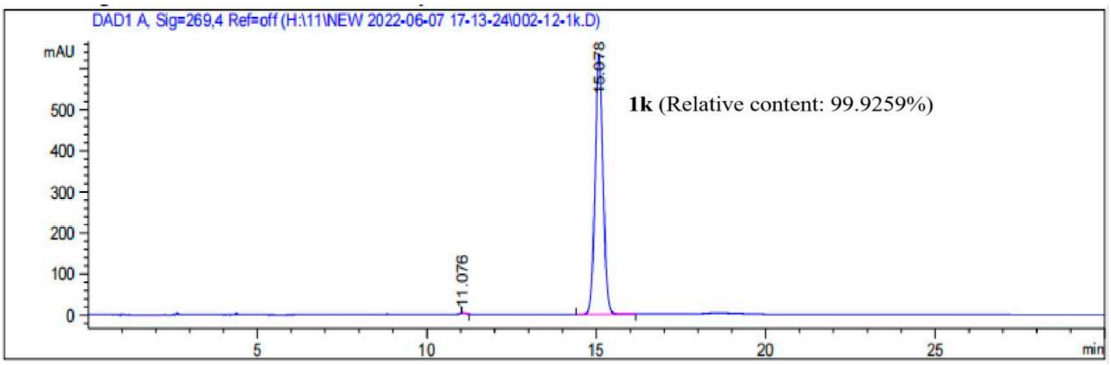

Figure S116. HPLC chromatogram of **1k.**

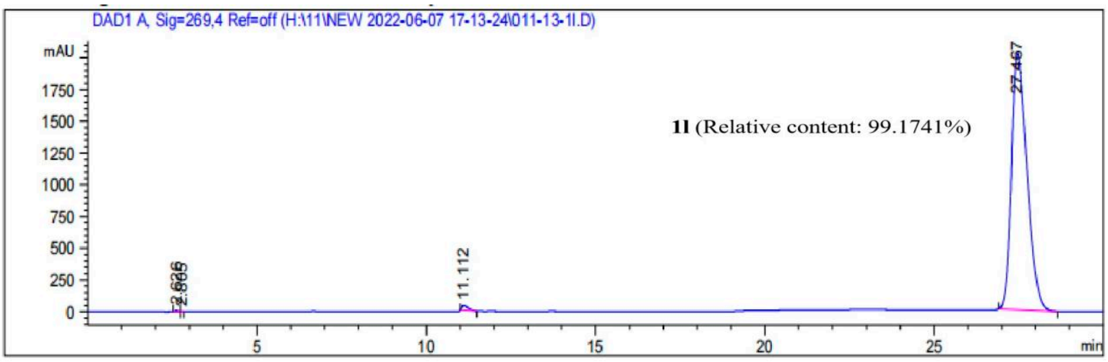

Figure S117. HPLC chromatogram of **1l.**

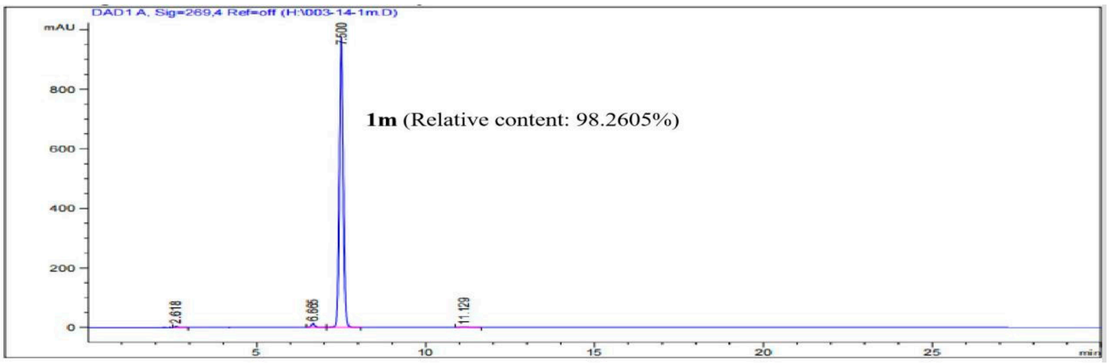

Figure S118. HPLC chromatogram of **1m.**

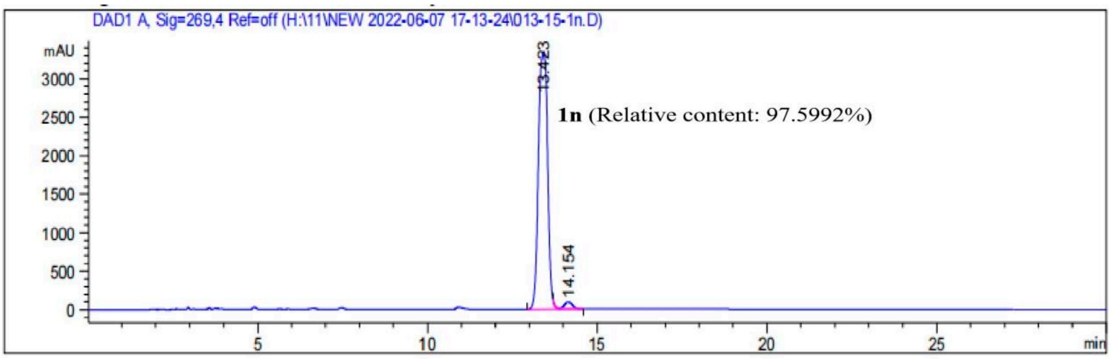

Figure S119. HPLC chromatogram of **1n.**

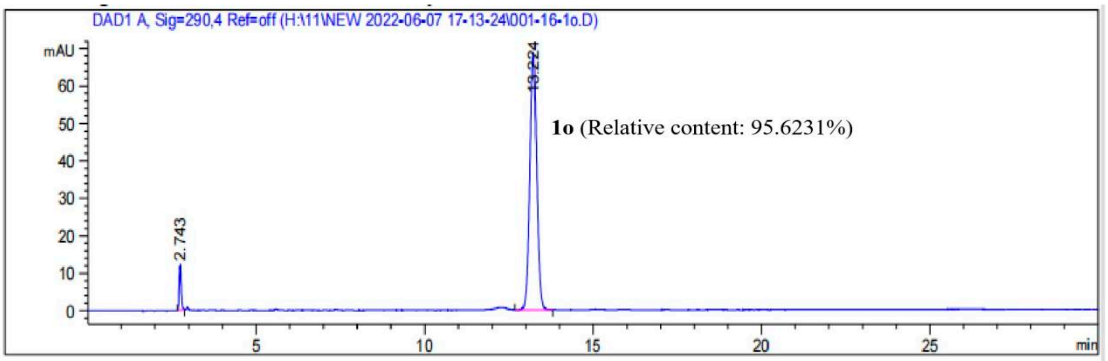

Figure S120. HPLC chromatogram of **1o.**

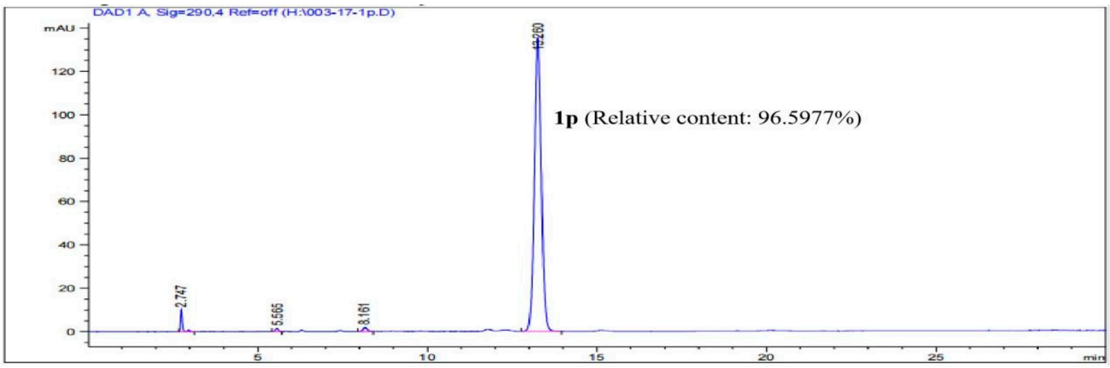

Figure S121. HPLC chromatogram of **1p.**

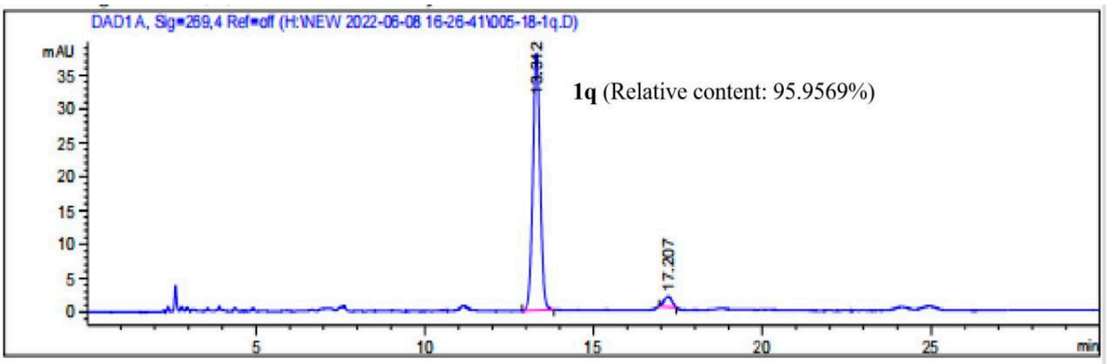

Figure S122. HPLC chromatogram of **1q.**

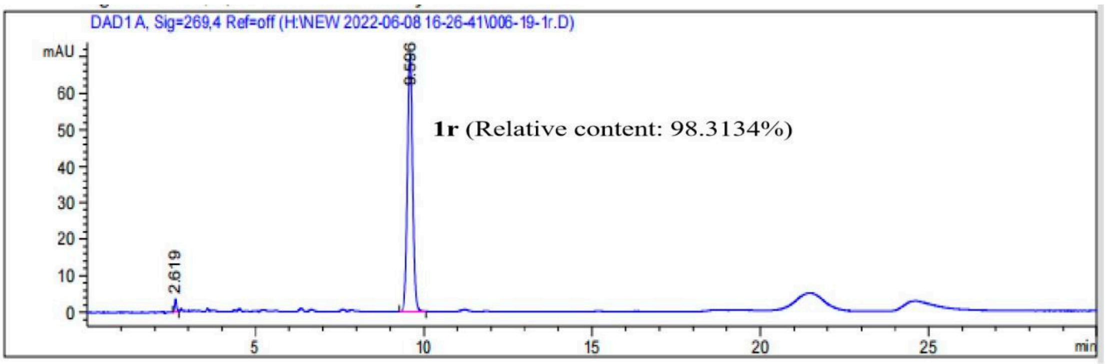

Figure S123. HPLC chromatogram of **1r.**

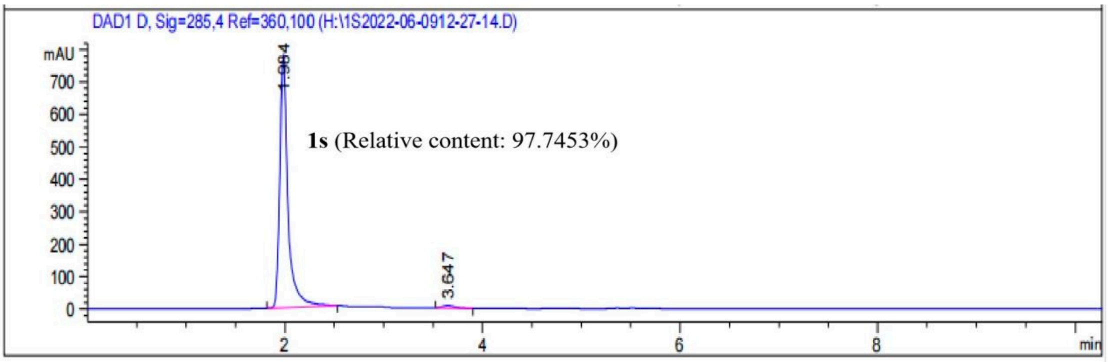

Figure S124. HPLC chromatogram of **1s.**

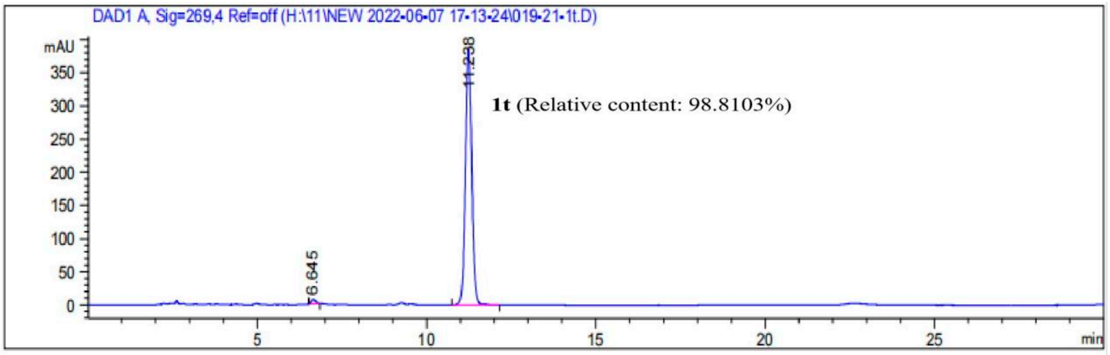

Figure S125. HPLC chromatogram of **1t.**

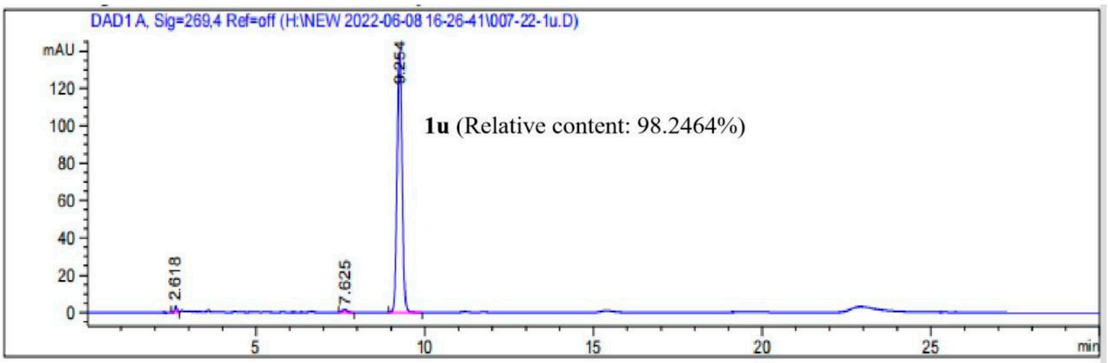

Figure S126. HPLC chromatogram of **1u.**

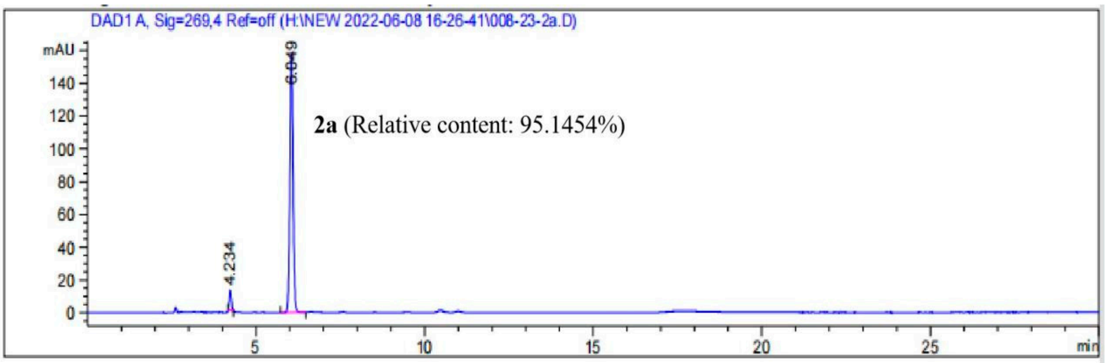

Figure S127. HPLC chromatogram of **2a.**

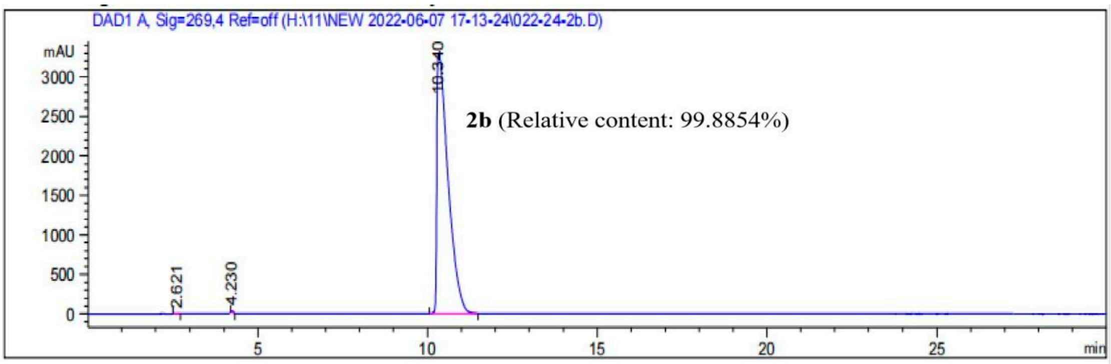

Figure S128. HPLC chromatogram of **2b.**

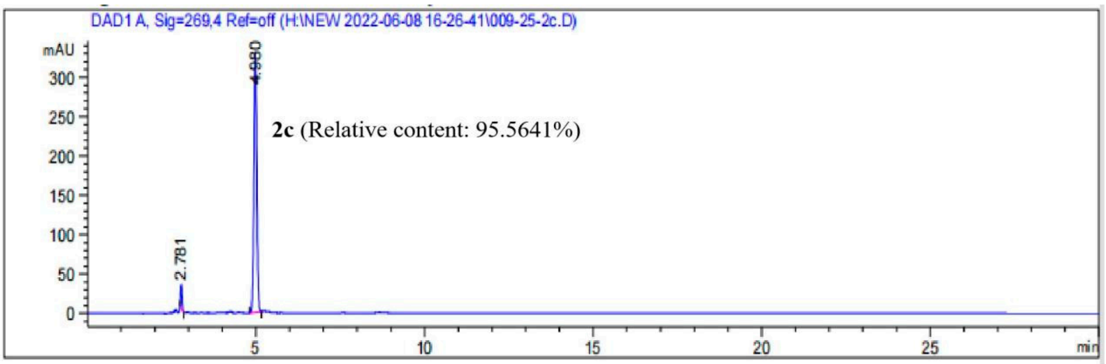

Figure S129. HPLC chromatogram of **2c.**

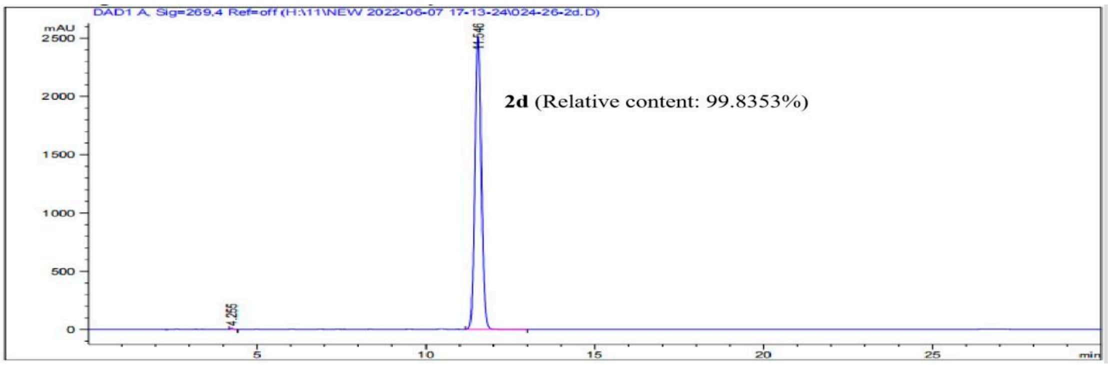

Figure S130. HPLC chromatogram of **2d.**

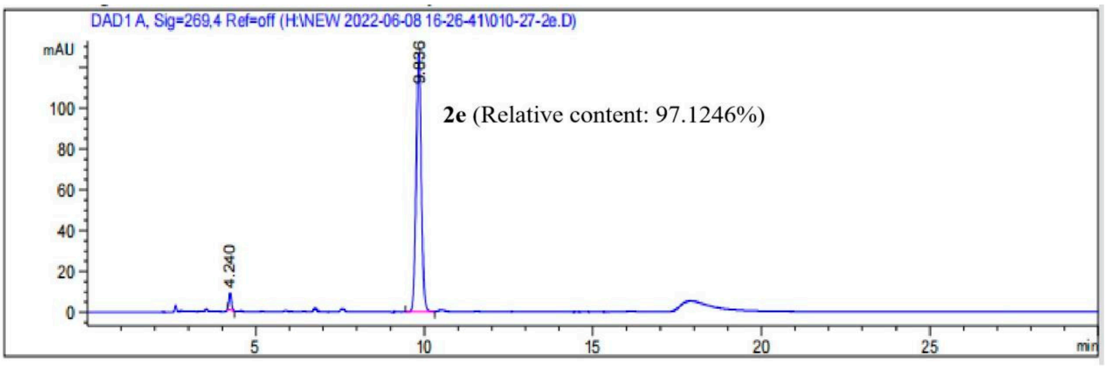

Figure S131. HPLC chromatogram of **2e.**

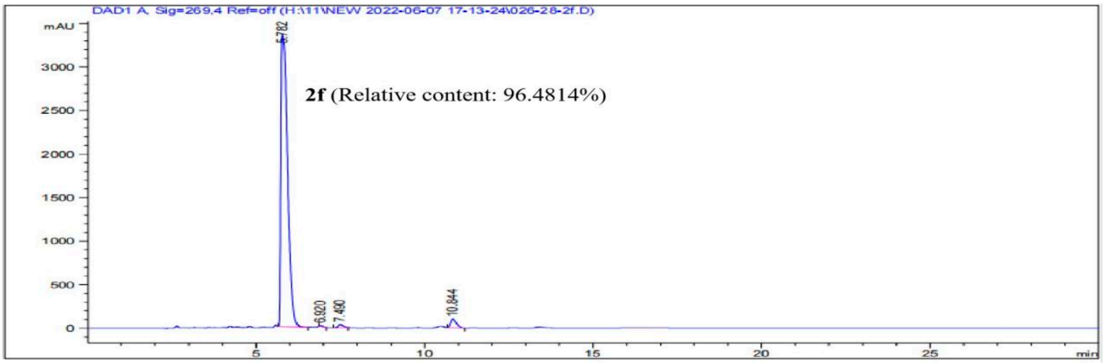

Figure S132. HPLC chromatogram of **2f.**

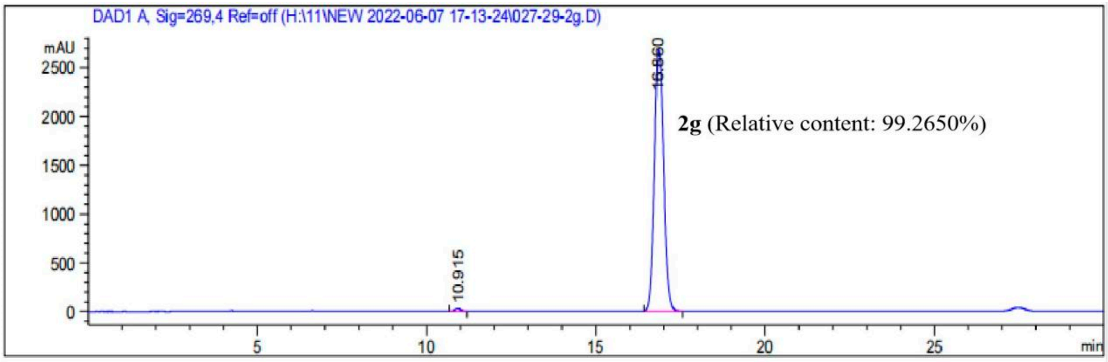

Figure S133. HPLC chromatogram of **2g.**

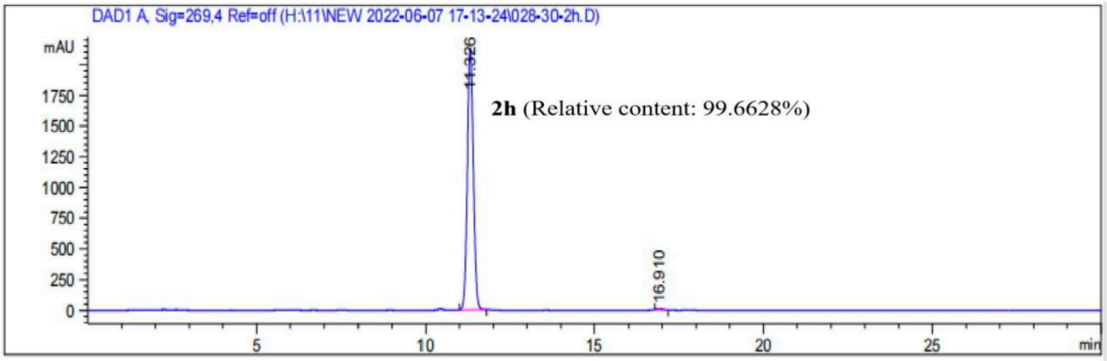

Figure S134. HPLC chromatogram of **2h.**

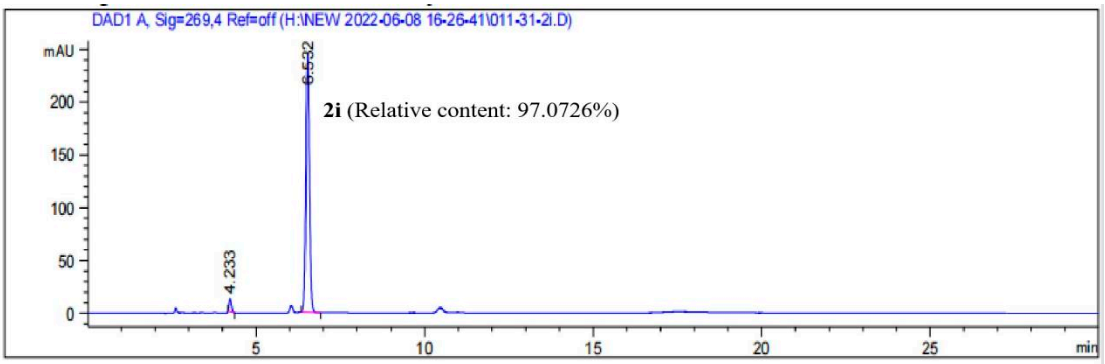

Figure S135. HPLC chromatogram of **2i.**

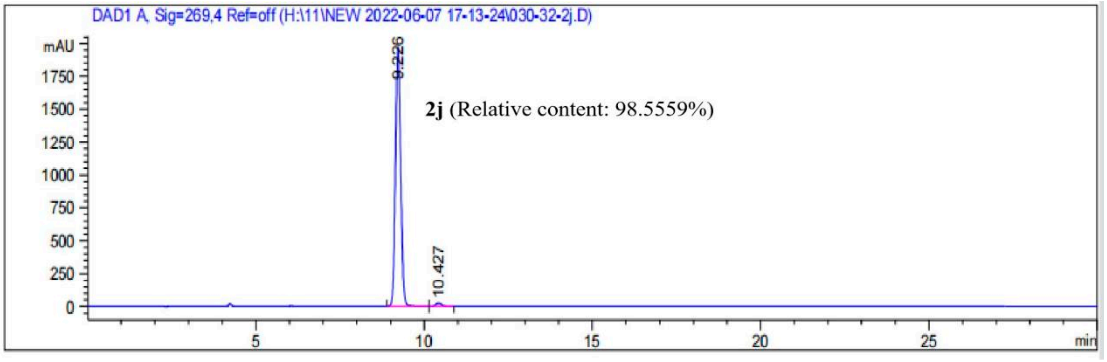

Figure S136. HPLC chromatogram of **2j.**

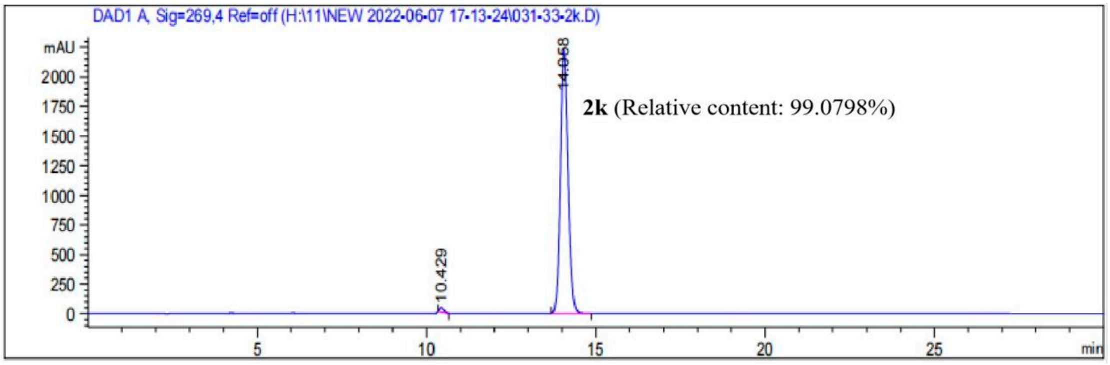

Figure S137. HPLC chromatogram of **2k.**

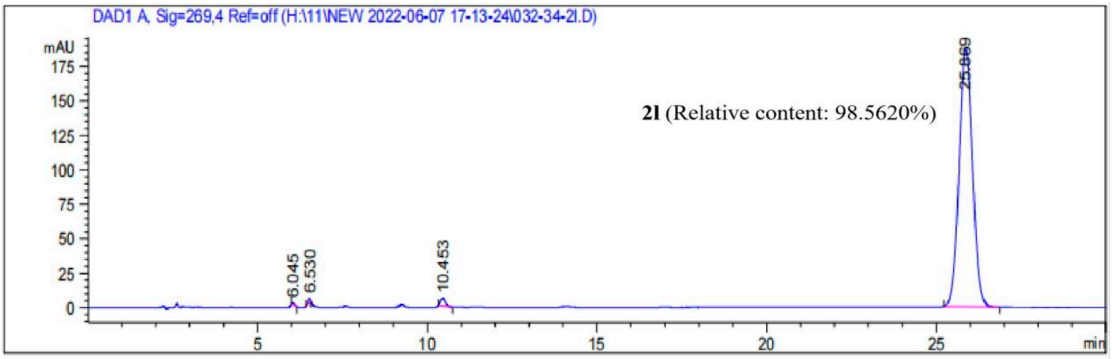

Figure S138. HPLC chromatogram of **2l.**

The preliminary screening results

Table S2. Preliminary screening test results.

Compd.	Concentration (μM)	Cell Inhibition（%）±SD				
		HL-60	K-562	MV-4-11	Jurkat, Clone E6-1	THP-1
**1**	20	93.25% ± 0.70%	21.44% ± 1.30%	87.01% ± 1.85%	96.77% ± 0.85%	93.36% ± 1.40%
**1a**	20	14.32% ± 5.75%	3.12% ± 2.16%	14.30% ± 2.69%	−0.61% ± 2.55%	7.53% ± 1.68%
**2**	20	97.28% ± 1.81%	90.59% ± 1.36%	89.27% ± 3.16%	97.79% ± 0.72%	95.46% ± 1.40%
**2a**	20	68.15% ± 4.39%	17.96% ± 5.52%	89.03% ± 2.46%	97.82% ± 0.70%	93.56% ± 1.57%

Trop. J. Pharm. Res. **2015**, *14*, p1803.
